# Analysis of 6.4 million SARS-CoV-2 genomes identifies mutations associated with fitness

**DOI:** 10.1126/science.abm1208

**Published:** 2022-05-24

**Authors:** Fritz Obermeyer, Martin Jankowiak, Nikolaos Barkas, Stephen F. Schaffner, Jesse D. Pyle, Leonid Yurkovetskiy, Matteo Bosso, Daniel J. Park, Mehrtash Babadi, Bronwyn L. MacInnis, Jeremy Luban, Pardis C. Sabeti, Jacob E. Lemieux

**Affiliations:** ^1^ Broad Institute of MIT and Harvard; 415 Main Street, Cambridge, MA 02142, USA. ^2^ Pyro Committee, Linux AI & Data Foundation; 548 Market St San Francisco, California 94104. ^3^ Department of Organismic and Evolutionary Biology, Harvard University; Cambridge, MA 02138, USA. ^4^ Department of Immunology and Infectious Diseases, Harvard T. H. Chan School of Public Health, Harvard University; Boston, MA, USA. ^5^ Program in Molecular Medicine, University of Massachusetts Medical School; Worcester, MA 01605, USA. ^6^ Research Department, Molecular Enzymology Division, New England Biolabs, Ipswich, MA 01938, USA. ^7^ Massachusetts Consortium on Pathogen Readiness; Boston, MA 02115, USA. ^8^ Ragon Institute of MGH, MIT, and Harvard; 400 Technology Square, Cambridge, MA 02139, USA. ^9^ Howard Hughes Medical Institute; 4000 Jones Bridge Rd, Chevy Chase, MD 20815, USA. ^10^ Division of Infectious Diseases, Massachusetts General Hospital; Boston, MA, USA.

## Abstract

Repeated emergence of SARS-CoV-2 variants with increased fitness underscores the value of rapid detection and characterization of new lineages. We have developed PyR_0_, a hierarchical Bayesian multinomial logistic regression model that infers relative prevalence of all viral lineages across geographic regions, detects lineages increasing in prevalence, and identifies mutations relevant to fitness. Applying PyR_0_ to all publicly available SARS-CoV-2 genomes, we identify numerous substitutions that increase fitness, including previously identified spike mutations and many non-spike mutations within the nucleocapsid and nonstructural proteins. PyR_0_ forecasts growth of new lineages from their mutational profile, ranks the fitness of lineages as new sequences become available, and prioritizes mutations of biological and public health concern for functional characterization.

The SARS-CoV-2 pandemic has been characterized by repeated waves of cases driven by the emergence of new lineages with higher fitness, where fitness encompasses any trait that affects the lineage’s growth, including its basic reproduction number (R_0_), ability to evade existing immunity, and generation time. Rapidly identifying such lineages as they emerge, and accurately forecasting their dynamics, is critical for guiding outbreak response. Doing so effectively would benefit from the ability to interrogate the entirety of the global SARS-CoV-2 genomic dataset. The large size (currently over 7.5 million virus genomes) and geographic and temporal variability of the available data present significant challenges that will become greater as more viruses are sequenced. Current phylogenetic approaches are computationally inefficient on datasets with more than ~5000 samples and take days to run at that scale. Ad hoc methods to estimate the relative fitness of particular SARS-CoV-2 lineages are a computationally efficient alternative (*1*–*3*), but have typically relied on models in which one or two lineages of interest are compared to all others and do not capture the complex dynamics of multiple co-circulating lineages.

Furthermore, estimates of relative fitness based on lineage frequency data alone (*2*, *4*, *5*) do not take advantage of additional statistical power that can be gained from analyzing the independent appearance and growth of the same mutation in multiple lineages. Performing a mutation-based analysis of lineage prevalence has the additional advantage of identifying specific genetic determinants of a lineage’s phenotype, which is critically important both for understanding the biology of transmission and pathogenesis and for predicting the phenotype of new lineages. The SARS-CoV-2 pandemic has already been dominated by several genetic changes of functional and epidemiological importance, including the spike (S) D614G mutation that is associated with higher SARS-CoV-2 loads (*6*, *7*). Mutations found in Variants of Concern (VoC), such as S:N439R, S:N501Y, and S:E484K, have been linked, respectively, to increased transmissibility (*8*), enhanced binding to ACE2 (*9*), and antibody escape (*10*, *11*). Despite these successes, identifying functionally important mutations in the context of a large background of genetic variants of little or no phenotypic consequence remains challenging.

In modeling the relative fitness of SARS-CoV-2 lineages, we estimated their growth as a linear combination of the effects of individual mutations. To this end, we developed PyR_0_, a hierarchical Bayesian regression model that enables scalable analysis of the complete set of publicly available SARS-CoV-2 genomes, that can be applied to any viral genomic dataset and to other viral phenotypes. The model, which is summarized in fig. S1, and described in detail in the supplementary materials, avoids the complexity of full phylogenetic inference by first clustering genomes by genetic similarity (refining PANGO lineages (*12*)), and estimating the incremental effect on growth rate of each of the most common amino acid changes on the lineages in which they appear. By regressing growth rate on genome sequence, the model shares statistical strength among genetically similar lineages without explicitly relying on phylogeny. By modeling only the multinomial proportion of different lineages rather than the absolute number of samples for each lineage (*13*, *14*), and by doing so within 14-day intervals in 1,560 globally-distributed geographic regions, the model achieves robustness to a number of sources of bias that affect all lineages, across regions and over time, including differences in data collection and changes in transmission due to such factors as social behavior, public health policy, and vaccination.

We fit PyR_0_ to 6,466,300 SARS-CoV-2 genomes available on GISAID (*15*) as of January 20, 2022, in a model that contained 3,000 clusters, derived from 1,544 PANGO lineages, and 2,904 nonsynonymous mutations. The output of the model is a posterior distribution for the relative fitness (exponential growth rate) of each lineage and for the contribution to the fitness from each mutation. Fitting this large model is computationally challenging, so we used stochastic variational inference, an approximate inference method that reduced our task to solving a 75-million-dimensional optimization problem on a GPU. Inference was implemented in the Pyro (*16*) probabilistic programming framework (see Supplemental Materials). The trained model can be used to infer lineage fitness, predict the fitness of completely new lineages, forecast future lineage proportions, and estimate the effects of individual mutations on fitness.

The model's lineage fitness estimates (Fig. 1B) show a modest upward trend over time among all lineages, interrupted by several lineages with much higher fitness. Sensitivity analyses revealed qualitative consistency of fitness estimates across spatial data subsets (fig. S2). The upward trend may in part reflect an upward bias caused by the lineage assignment process, as can be seen in simulation studies (fig. S3), but the high tail of the distribution exhibits elevated fitness values far in excess of this trend. The spread of the virus into human populations in late 2019 and early 2022 has been marked by periods of rapid evolution in fitness and waves of increase in case counts (Fig. 1). While PANGO lineages facilitate communication by providing a stable nomenclature, we observed some PANGO lineages with multiple successive peaks in some regions, suggesting that sublineages within them had differing fitnesses. We therefore algorithmically refined the 1,544 PANGO lineages into 3,000 finer clusters, and found that our model identified significant heterogeneity within some PANGO lineages (fig. S4). When we tested the model's predictive ability (fig. S5), we found that forecasts were reliable for 1-2 months into the future for variants of concern, but not necessarily other variants, when they tended to be disrupted by the emergence of a completely new strain (table S1, fig. S6). The accuracy of forecasts stabilized typically stabilized within two weeks after the emergence of a new competitive lineage in a region (fig. S6).

**Fig. 1.**
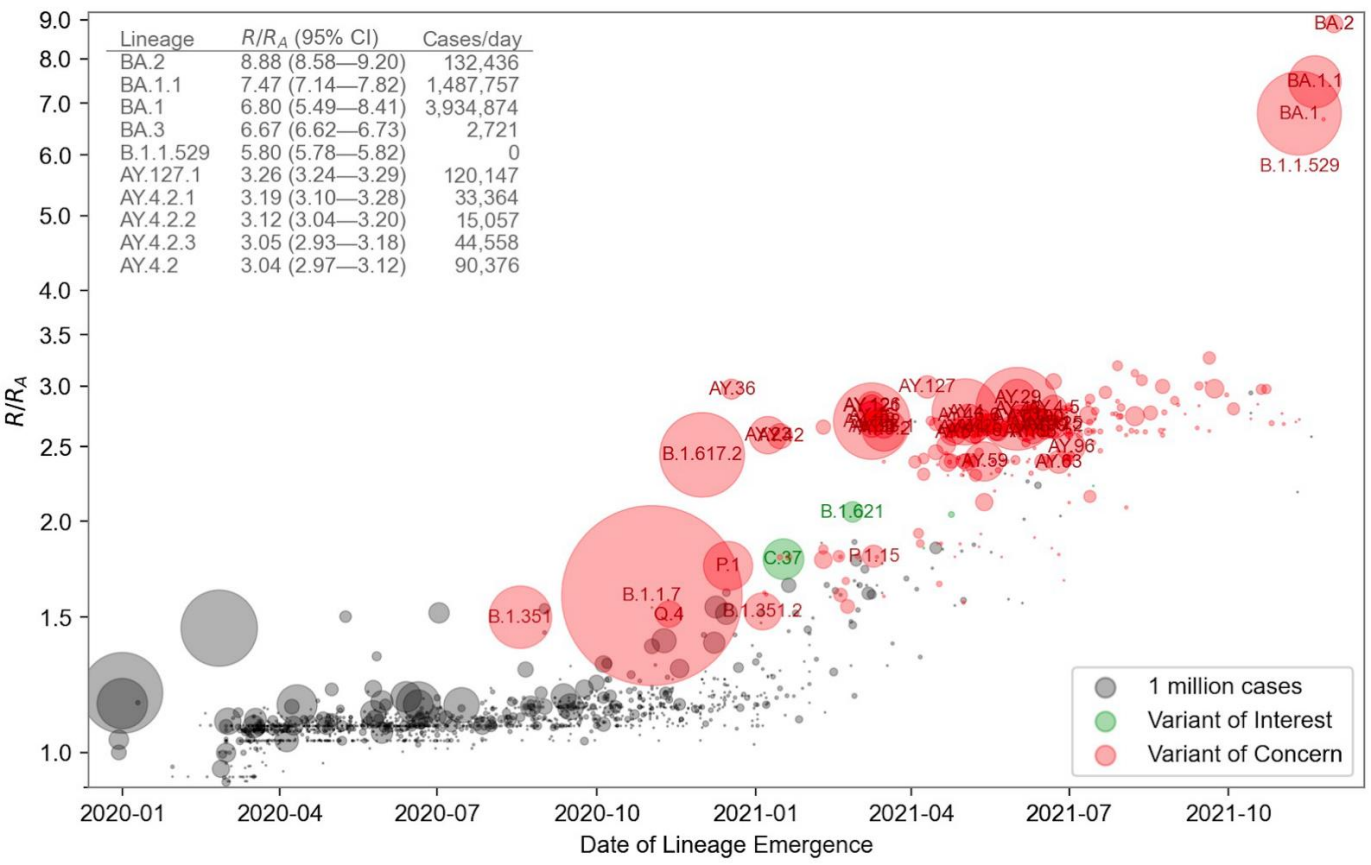
Relative fitness versus date of lineage emergence. Circle size is proportional to cumulative case count inferred from lineage proportion estimates and confirmed case counts. Inset table lists the 10 fittest lineages inferred by the model. R/R_A_ is the fold increase in relative fitness over the Wuhan (**A**) lineage, assuming a fixed generation time of 5.5 days.

The model correctly infers WHO classification variant Omicron (PANGO BA.2) to have the highest fitness to date, 8.9-fold (95% CI, 8.6-9.2) higher than the original A lineage (Fig. 1 inset), accurately foreshadowing its rise in regions where it is circulating (fig. S7). Through systematic backtesting, we found that the model would have provided early warning and aided in the identification of VoCs had it been routinely applied to SARS-CoV-2 samples, confirming the importance for public health of timely publication of genomic data. For example, the elevated fitness of BA.2 was identified by mid-December 2021 on the basis of 76 reported sequences (fig. S8); sharing statistical strength over mutations enabled an earlier and more confident prediction that BA.2 was the fittest lineage yet observed (fig. S10). Likewise, PyR_0_ would have forecast the dominance of B.1.1.7 in late November 2020 (fig. S9), AY.4 by May 2021 (fig. S10), and BA.1 by early December 2021 (fig. S8). While variant-specific models were accurate and useful in predicting the rise of these lineages (*2*), each modeling effort was specific to a particular lineage and geographic region. PyR_0_’s global approach provides similar early detection while also offering automated, rapid, and standardized unbiased consideration of all variants and lineages, together with ranking based on relative fitness.

Compared to standard multinomial regression models, PyR_0_ estimates of lineage fitness were similar (Pearson’s R = 0.95, S11-S12), but including mutations in the model enables PyR_0_ to infer elevated fitness of Omicron lineages BA.1 and BA.2 faster than the model without mutations (fig. S14). In contrast to non-hierarchical binomial logistic regression (fig. S13), PyR_0_ estimates displayed less variability as data accumulated, benefitting from the sharing of information across regions and the regularizing effect of the priors. Lineage fitness estimates were also stable between our initial analysis of 2.1 million genomes in August 2021 (*17*), shortly after the emergence of Delta lineages, and before the emergence of Omicron (Spearman’s rho = 0.78, fig. S15C). The correlation between individual amino acids in the two models was weaker than that for lineages (fig. S15D-E, rho = 0.48) but still significant (test of no association for rho, p < 2 x 10^−16^), reflecting both the inherent difficulty of estimating high-dimensional mutational coefficients observed indirectly through lineage counts (Supplementary Note 1), as well as the addition of 4.3 million sequences, including highly fit Omicron lineages distinguished by their enhanced immune escape.

By jointly modeling fitness estimates using lineage counts and individual mutations, PyR_0_ harnesses convergent evolution (Table 1 and fig. S16) to infer the fitness of new constellations of mutations based on the trajectories of other lineages in which they have previously emerged. This predictive capability has the potential to aid public health efforts because the model has the potential to learn faster by incorporating mutations than it would by relying on lineage counts alone (fig. S14). To test the reliability of this kind of estimate, we fit leave-one-out estimators for PANGO lineages on subsets of the dataset with that entire lineage removed, based solely on the mutational content of the omitted lineage (fig. S17). These estimators showed excellent agreement with estimators based on the observed behavior of the lineages, and they were also more accurate than naive phylogenetic estimators that assume the fitness of each new strain is equal to its parent lineage's fitness (Pearson's R = 0.983, after correcting for parent fitness, fig. S17). Together, these analyses suggest that PyR_0_ has the potential to aid genomic surveillance efforts by providing an automated early warning system on a similar time scale as sophisticated regional surveillance efforts (*18*, *19*).

**Table 1.** Amino acid substitutions most significantly associated with increased fitness. Significance is defined as posterior mean / posterior standard deviation. Fitness is per 5.5 days (estimated generation time of the Wuhan (A) lineage (*1*, *23*)). Final c​​olumn: number of PANGO lineages in which each substitution emerged independently.

**Rank**	**Gene**	**Substitution**	**Fold Increase in Fitness**	**Number of** **Lineages**
1	S	H655Y	1.051	33
2	S	T95I	1.046	30
3	ORF1a	P3395H	1.039	5
4	S	N764K	1.04	6
5	ORF1a	K856R	1.039	2
6	S	S371L	1.041	3
7	E	T9I	1.04	5
8	S	Q954H	1.04	5
9	ORF9b	P10S	1.039	25
10	S	L981F	1.04	2
11	N	P13L	1.04	25
12	S	G339D	1.039	4
13	S	S375F	1.04	5
14	S	S477N	1.039	47
15	S	N679K	1.04	11
16	S	S373P	1.04	5
17	M	Q19E	1.039	5
18	S	D796Y	1.038	11
19	S	N969K	1.04	5
20	S	T547K	1.038	3

Genome-wide estimates of the effect of SARS-CoV-2 mutations on fitness also provide a powerful tool for better understanding the biology of fitness. Our model allowed us to estimate the contribution of 2,904 amino acid substitutions (Fig. 2A and Table 1) to lineage fitness and to rank them by inferred statistical significance (fig. S18). Cross-validation confirmed that these results replicate qualitatively across different geographic regions (fig. S19).

**Fig. 2.**
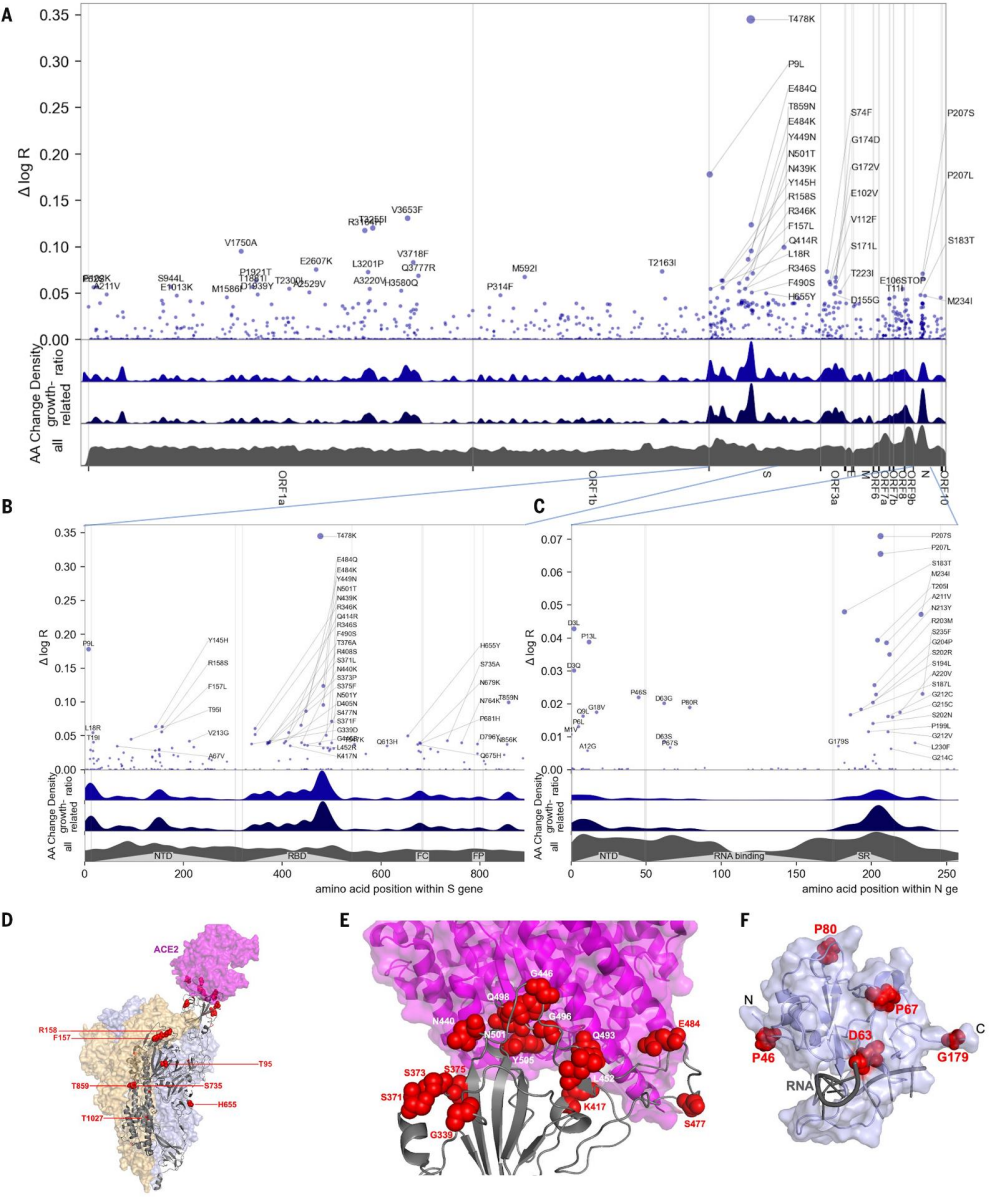
Manhattan plot of amino acid changes assessed in this study. (**A**) Changes across the entire genome. (**B**) Changes in the first 850 amino acids of S. In each of (A) to (C) the y axis shows effect size Δ log R, the estimated change in log relative fitness due to each amino acid change. The bottom three axes show the background density of all observed amino acid changes, the density of those associated with growth (weighted by |Δ log R|), and the ratio of the two. The top 55 amino acid changes are labeled. See fig. S13 for detailed views of S, N, ORF1a, and ORF1b. **C.** Changes in the first 250 amino acids of N. (**D**) Structure of the spike-ACE2 complex (PDB: 7KNB). Spike subunits colored light blue, light orange, and gray. Top-ranked mutations are shown as red spheres. ACE2 is shown in magenta. (**E**) Close-up view of the RBD interface. (**F**) Top-ranked mutations in the N-terminal RNA-binding domain of N. Residues 44-180 of N (PDB: 7ACT) are shown in light blue. Amino acid positions corresponding to top mutations in this region are shown as red spheres. A 10-nt bound RNA is shown in gray.

The highest concentrations of fitness-associated mutations were found in the S, N, and the ORF1 polyprotein genes (ORF1a and ORF1b, Fig. 2, A and B, and figs. S20 and S21). Using spatial autocorrelation as a measure of spatial structure, we found evidence of functional hotspots in the S, N, ORF7a, ORF3a, and ORF1a genes (table S2). Within S, we confirmed three hotspots of fitness-enhancing mutations, each within a defined functional region: the N-terminal domain, the receptor-binding domain (RBD), and the furin-cleavage site (Fig. 2B). We assessed mutational enrichment in the top-ranked set of mutations and identified an enrichment for lysine to asparagine mutations in the S gene (fig. S22C). We visualized top scoring mutations within atomic structures for the spike protein (Fig. 2, D to E), the nucleocapsid's N-terminal domain (Fig. 2F), the polymerase (fig. S23), and two proteases (fig. S24). Many of the top mutations in the S gene occurred in the receptor binding domain (RBD) making direct contacts with the ACE2 receptor, including K417N/T and E484K (Fig. 2, D to E). Two top-ranked mutations, T478K and S477N, occur in a flexible loop adjacent to the S-ACE2 interface (Fig. 3E), suggesting that these mutations may affect the kinetics of receptor engagement or the Spike conformational changes that follow. Other mutations occurred in regions proximal to essential enzymatic active sites of the viral replication (fig. S15) or protein processing (fig. S16) machinery.

**Fig. 3.**
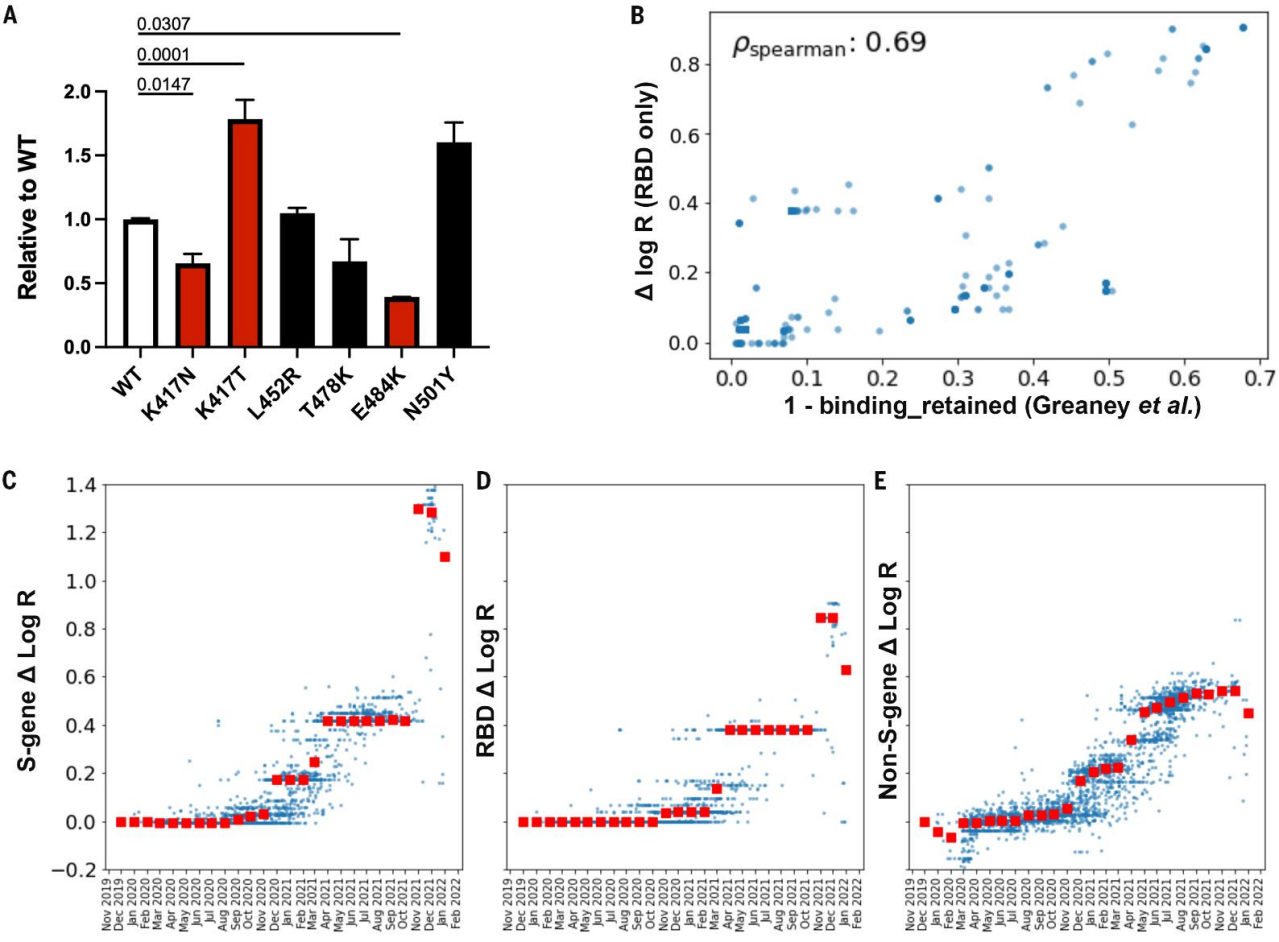
(A) Infectivity relative to WT of lentiviral vectors pseudotyped with the indicated Spike mutants. Target cells were HEK293T cells expressing ACE2 and TMPRSS2 transgenes. The genetic background of the Spike was Wuhan-Hu-1 bearing D614G. Red bars were significantly different from WT (adjusted p values shown). Black bars were not significantly different from WT. (**B**) For the 1701 SARS-CoV-2 clusters with at least one amino acid substitution in the RBD domain we compare: i) the PyR_0_ prediction for the contribution to Δ log R from RBD substitutions only; to ii) antibody binding computed using the antibody-escape calculator in (*20*). The escape calculator is based on an intuitive non-linear model parameterized using deep mutational scanning data for 33 neutralizing antibodies elicited by SARS-CoV-2. PyR_0_ predictions exhibit high (Spearman) correlation with predictions from Greaney et al. (*20*) (**C **to** E**) We dissect PyR_0_ Δ log R estimates into S-gene (C), RBD (D), and non-S-gene (E) contributions for 3000 SARS-CoV-2 clusters (blue dots). The horizontal axis corresponds to the date at which each cluster first emerged. Red squares denote the median Δ log R within each monthly bin. The increased importance of S-gene mutations (notably in the RBD) over non-S-gene mutations starting around November 2021 is apparent.

We tested several of the high-scoring mutations in single-cycle infectivity assays as done previously (*7*), focusing on the RBD (Fig. 3A). We found that while some individual mutations increased infectivity, on average, high-scoring RBD mutations did not promote infectivity per se. We considered an alternate possibility that fitness of Spike mutations is driven by immune escape. Using RBD-aggregated mutations as a proxy for immune escape, we found that the fitness effect of these Spike mutations correlates well with antibody escape estimates from Greaney et al. (*20*) (Fig. 3B). Together with the observed jump in fitness beginning in late 2021 (Fig. 3C) associated with Spike mutations, but not mutations elsewhere in the genome (Fig. 3E), these results suggest that immune escape is the dominant driver of current fitness increases. BA.1 and BA.2 had similar estimated fitness from Spike mutations, potentially consistent with similar Spike antibody neutralization of these variants (*21*), whereas PyR_0_ inferred that the elevated fitness of BA.2 is attributed to non-Spike mutations (fig. S25). In contrast to mutations in Spike, those in the serine-arginine rich region of N were linked to increased efficiency of SARS-CoV-2 genomic RNA packaging (*22*). Within ORF1, we found fitness-associated mutations across all viral enzymes, and clusters within additional non-structural proteins (nsps). The highest concentration of fitness-associated mutations is found in nsp4, nsp6, and nsp12–14 (fig. S12B,S13C-D), suggesting unexplored function at those sites. For example, nsp4 and nsp6 have roles in assembly of replication compartments, and substitutions in these regions may influence the kinetics of replication (see Supplemental Note 3). We caution that while convergent evolution makes it possible to identify candidate functional mutations, observational data alone is insufficient to declare mutations as causal rather than merely correlated. Our uncertainty-ranked list of important mutations can be used to prioritize hits identified by our study for functional follow-up.

Some lineages increased in fitness more than others over the course of the pandemic (fig. S4). Notably, B.1.1 displayed the greatest variability among sublineages, followed by B.1. Fitness appeared to reach a plateau over time for most lineages (Fig. 1 and fig. S4). In contrast to Omicron sublineages, Alpha and Delta showed little variability in Spike-attributable fitness (fig. S25), suggesting that the propensity to acquire new Spike mutations depends on the constellation of mutations that comprise a lineage, consistent with epistasis. A limitation of PyR_0_ is that it does not incorporate epistatic interactions between mutations (Supplemental Note 1); however, our results demonstrate the feasibility of inferring genetic determinants and lineage fitness using the simplest possible linear-additive model and provide a foundation for future research for more complex modeling that includes epistatic effects between mutations and migration across geographic regions.

In summary, PyR_0_ provides a genome-wide, automated approach for detecting viral lineages with increased fitness. By combining a model-based assessment of lineage fitness with absolute case counts, our model provides a global picture of the events of the first two years of the pandemic. Because it assesses the contribution of individual mutations and aggregates across all lineages and geographic regions, it can identify mutations and gene regions that likely increase fitness, and mutation-level information may help detect fitter lineages earlier than case counts alone. Applied to the full set of publicly available SARS-CoV-2 genomes, it provides a genomic view of the mutations driving increased fitness of the virus, identifying experimentally established driver mutations in S and highlighting the key role of non-S mutations, particularly in N, ORF1b, and ORF1a, which have received relatively less research attention. By modeling millions of viral sequences across thousands of regions, PyR_0_ yields mechanistic insight into viral fitness and offers a panoramic view of viral evolution, revealing a pattern whereby major circulating lineages fragment into sublineages with modest differences in fitness before they are collectively displaced by the sudden emergence of markedly fitter variants.

## Supplementary Materials

### This PDF file includes:

Materials and Methods Figures S1 to S34 Tables S1 to S5 References (*25* *–* *56*) MDAR Reproducibility Checklist Data S1 to S5 GISAID Acknowledgments table

### Other Supplementary Material for this manuscript includes the following:

MDAR Reproducibility Checklist
