# Supplementary material for: Analysis of 6.4 million SARS-CoV-2 genomes identifies mutations associated with fitness
Source: Science. 2022 May 24:abm1208. doi: 10.1126/science.abm1208 (PMC9161372; doi:10.1126/science.abm1208)
Supplement: Supplementary file 1 — Materials and Methods Figures S1 to S34 Tables S1 to S5 References ( 25 – 56 ) MDAR Reproducibility Checklist Data S1 to S5 GISAID Acknowledgments table [file science.abm1208_sm.pdf]

Science
AAAS

Supplementary Materials for

Analysis of 6.4 million SARS-CoV-2 genomes identifies mutations associated with fitness

F. Obermeyer *et al.*

Corresponding authors: F. Obermeyer, fritz.obermeyer@gmail.com; Jacob E. Lemieux, lemieux@broadinstitute.org

DOI: 10.1126/science.abm1208

The PDF file includes:

Materials and Methods
Supplementary Text
Figures S1 to S34
Tables S1 to S5
References

Other Supplementary Material for this manuscript includes the following:

MDAR Reproducibility Checklist
Data S1 to S5

Supplementary Materials

Materials and methods

Data and Code Availability

Source code for data preprocessing and modeling and available at https://github.com/broadinstitute/pyro-cov. GISAID sequence data is publicly available at https://gisaid.org. PANGO lineage aliases are available at https://cov-lineages.org with source code at https://github.com/cov-lineages/lineages-website and lineage aliases available at https://github.com/cov-lineages/pango-designation. UShER phylogenies of public data are available at http://hgdownload.soe.ucsc.edu/goldenPath/wuhCor1/UShER_SARS-CoV-2. The whole genome map is available as part of NextClade at https://github.com/nextstrain/ncov/blob/50ceffa/defaults/annotation.gff. Structures of ORFab regions are available at https://www.ncbi.nlm.nih.gov/protein.

Regression model of relative fitness (PyR$_0$ model)

We fit a Bayesian, hierarchical multinomial logistic regression model to data from GISAID using Pyro. Details are provided in the supplemental note below.

Simulation of lineages

We carried out a simulation study to determine whether the process of clustering genomes into named lineages could generate an artifactual increase in estimated fitness. The simulation was of a single neutrally evolving viral population with discrete generations and a stochastic population size generated by a highly overdispersed negative binomial distribution with the current fitness. (Overdispersion parameter = 0.11, which yields 10% of cases causing 80% of transmission.) The fitness is 2.5 for the first 10 generations; subsequently it drops to 1.5 until the viral population reaches 80,000 infections, whereupon it drops again to 0.8. When the population decreases to 10,000, the growth switches back to 1.5, and continues cycling when the high and low population thresholds are reached. (A model with a roughly constant-sized population yields similar results.) The population starts as a single named lineage. Each generation, the most successful nodes in that generation are determined by looking ahead four generations and counting descendants. New lineages are assigned to the nodes with the most descendants (minimum of 200 descendants), up to a maximum of 10 lineages per generation. 10% of all infections are randomly sampled and any lineage with fewer than 20 descendants is discarded. When all new lineages have been generated and all nodes assigned a lineage, a global multinomial logistic regression is performed, using the Python package sklearn.linear_model, yielding relative fitness estimates of all lineages.

Spatial analysis of mutation coefficients

To assess the spatial structure of the inferred amino acid coefficients β_f (described in Probabilistic Model below), we utilize the Moran I spatial autocorrelation statistic. We report (see Table S1) one-sided p-values for Moran I computed using a permutation test with 999,999 random permutations. We use a gaussian weighting function of the form exp(-distance^2/lengthscale^2), where distance is measured in units of nucleotides. We compute Moran I statistics both for individual genes and the entire genome. For larger genes whose extent is 1000+ nucleotides we use a length scale of 50 nucleotides. For smaller genes (e.g. ORF8) we set

the length scale to one twentieth of the extent of the gene. When considering the entire set of amino acid changes, i.e. all 2,904 coefficients that make up β_f, we compute the Moran I statistic for two different length scales. We note, however, that the Moran I statistic is somewhat simplistic, since it is designed to pick up spatial structure at a single length scale. In particular it can be insensitive to complex spatial structure that involves multiple overlapping substructures at different scales. Nevertheless it offers a simple quantitative metric for identifying spatial structure in the coefficients β_f.

Analysis of substitution statistics

To assess enrichment of amino acid changes we compared the event frequencies for the leading mutation sets (as determined by posterior mean/std ranking) against a background of all mutations used as features in the model using multiple testing corrected binomial tests. We performed this analysis for both the asymmetric case (where A->V and V->A are different events) and for the symmetric case.

Comparison to other regression models

We fit non-hierarchical logistic regression models in R version 4.0.3. The stats::glm() was used to fit binomial logistic regression models and the nnet::multinom function was used to fit multinomial logistic regression models. For multinomial logistic regressions, the data were filtered to contain sequences between January 1 2021 and December 31 2021 from the most common 25 PANGO lineages in the 10 countries with the most sequences available. The resulting dataset was downsampled to 10% of its initial size.

We compared PyR_0 to PyR_0-NoMut, a hierarchical, multinomial, Bayesian model identical to PyR_0 except that the mutations were removed from the model as follows:

$\sigma_5 \sim \text{LogNormal}(-4, 2)$ [new scalar latent variable]
$\text{log\_rate\_c} \sim \text{Normal}(0, \sigma_5)$ [lineage-level log growth rate]
$\boldsymbol{\beta}_{pc} \sim \text{Normal}(\text{log\_rate\_c}, \sigma_4)$ [region-local lineage-specific log growth rate]

We also compare PyR_0 as described below to an earlier version of the model run on 2.1 million genomes. This earlier version was similar to the present version, but contained some minor differences, detailed in *(17)*, that were updated as we improved and simplified the model. These differences include the former use of PANGO lineages which have been replaced by fine-grained phylogenetic clustering, the inclusion of deletions (which we now exclude because they cannot be placed on the mutation-annotated tree created by USHER), a logistic prior rather than a simpler Laplace prior, and a more factorized variational posterior distribution which has been replaced by a simpler distribution.

Supplemental Note 1: Detailed description of PyR_0 model

Data Preparation

We downloaded 6,466,300 samples from GISAID *(15)* on January 20, 2021. Each sample record includes labels for time, location, PANGO lineage annotation *(12)*, and genetic sequence. We discard records with missing time, location, or lineage. We use UShER*(25)* to build a mutation-annotated phylogenetic tree, discarding sequences whose alignment quality is not reported as "good". We bin time intervals into 14-day segments, choosing a multiple of 7 to minimize

weekly seasonality, but binning coarser than a week so as to reduce memory requirements; this results in 56 time bins.

Because sample counts vary widely across GISAID geographic region (by as much as five orders of magnitude), we aggregate regions into the following coarse partitions: each country counts as a region, and any first level subregion of a country counts as a region if it has at least 50 samples; otherwise it is aggregated into a whole-country bin. Note this means that e.g. a country may be split up into its larger regions, with smaller regions being subsumed into an aggregate country level bin. We then drop regions without samples in at least two different time intervals, resulting in 1560 regions in total. Figure S26 shows the distribution of samples among countries and GISAID regions.

After preprocessing, the model input data are a $T \times P \times C = 56 \times 1560 \times 3000$ shaped array $y_{tpc} \in \mathbb{N}$ of counts (this array is sparse and our inference code uses a sparse representation), and an $C \times F = 3000 \times 2904$ shaped array $X_{sf} \in \{0, 1\}$ of mutation features.

Cases per day (see Figure 3 inset) were estimated by multiplying confirmed case count data from Johns Hopkins University by the estimated proportion of each lineage within each (time, region) bin. We manually matched each GISAID region to the finest enclosing JHU region.

Lineage Clustering

Our method relies on a partitioning of genetic samples into clusters, where we estimate the fitness of each cluster. We initially tried to use the 1544 PANGO lineages as clusters, but found that some PANGO lineages appeared to include multiple distinct viruses of different fitness, e.g. B.1.1. exhibits two peaks in relative abundance in England, contrary to our multivariate logistic growth model. We therefore refined the 1544 PANGO lineages into 3000 finer clusters, with rates estimated individually for each cluster. Indeed Figure S4 shows that some PANGO lineages contain multiple distinct clusters of fitness estimates differing by more than a factor of two.

To create genetic clusters finer than PANGO lineages we began with a complete 4,833,238 node phylogeny of all GISAID samples maintained by Angie Hinrichs *(26)* (this phylogeny was created using UShER*(25)*, excluding private mutations, masking difficult-to-sequence regions, eliding deletions, parsimoniously imputing missing sequence data). To coarsen the 4,833,238-node phylogenetic tree down to 3000 nodes (treated as clusters) we greedily collapsed parent-child edges, minimizing the the following distance function *TreeDistance*(-,-) between two mutation annotated trees

$$TreeDistance(T,T') = \sum_{u} \sum_{v} EditDistance(mrca(T,u,v), mrca(T',u,v)))$$

where T is the true mutation annotated tree, T' is the collapsed tree whose nodes we treat as clusters, u and v are sample sequences, *mrca*(T,u,v) is the sequence of the most recent common ancestor of u,v in the mutation annotated tree T, and *EditDistance*(-,-) counts amino acid substitutions between two sequences. This objective function minimizes the mean edit distance between the true mrca sequence and its cluster's sequence, for each pair of sequences. Changes in the objective function can be computed cheaply, and the O(n log(n)) time greedy algorithm can process the entire n=4,833,238 node phylogeny in under 5 minutes. Empirically this heuristic clustering produces trees that are approximately balanced in both cluster size and cluster-cluster

edit distance, on both the true data and on synthetic datasets. Figure S26 shows the distribution of samples among both coarse PANGO lineages and the finer clusters. Figure S28 shows small example trees produced by clustering large synthetic trees.

Probabilistic Model

We model relative lineage growth with a hierarchical Bayesian regression model with a multinomial likelihood. Arrays in the model index over one or more indices: T=56 time steps (increments of 14 days) t; C=3000 clusters c; P=1560 regions ("places") p; and F=2904 amino acid substitutions ("features") f. The model, shown below, regresses lineage counts $y_{tpc} \in \mathbb{N}$ in each time-region-lineage bin against amino acid mutation covariates $X_{cf} \in \{0,1\}$. The variables y and X are observed and all other variables in the model are latent. Each latent variable is governed by a prior distribution. The full model is specified as follows (visualized in Figure S29), where the observed counts y_{tpc} are underlined:

$$\alpha_c \sim \text{Normal}(0, \sigma_1) \qquad \sigma_1 \sim \text{LogNormal}(0, 2)$$

$$\alpha_{pc} \sim \text{Normal}(\alpha_c, \sigma_2) \qquad \sigma_2 \sim \text{LogNormal}(0, 2)$$

$$\beta_f \sim \text{Laplace}(0,\ \sigma_3) \qquad \sigma_3 = \frac{1}{2000}$$

$$\beta_{pc} \sim \text{Normal}\Big(\sum_f \beta_f X_{cf},\ \sigma_4\Big) \qquad \sigma_4 \sim \text{LogNormal}(-4, 2)$$

$$\underline{y_{tp.}} \sim \text{Multinomial}\Big(\sum_c y_{tpc},\ \text{softmax}(\alpha_{p.} + t\beta_{p.}/\tau)\Big)$$

The proportion of lineages in a single time-region bin is modeled as a Multinomial distribution whose probability parameter is a multivariate logistic growth function softmax($\alpha_{p\cdot} + t\beta_{p\cdot}/\tau$) with intercept α_{pc} and slope β_{pc} in units of generation time τ = 5.5 days (these units are for interpretability only; the model does not use the notion of generation, and thus is robust to changes in generation time). Here the dot subscripts $\alpha_{p\cdot} \in \mathbb{R}^C$, $\beta_{p\cdot} \in \mathbb{R}^C$, and $y_{tp.} \in \mathbb{N}^C$ denote vectors over cluster ids. The softmax function implements the multivariate generalization of logistic growth, inputting and outputting vectors, and is defined as

$$\text{softmax}(x)_i = \frac{\exp(x_i)}{\sum_j \exp(x_j)},$$

For a simple model of two lineages, each of the two components of the softmax function are sigmoid curves; however for more lineages, the functional forms may be more complex. Early iterations of the model used overdispersed likelihoods such as Dirichlet-Multinomial to account for additional variability not directly encoded in the generative process. However, we found that we can obtain much more accurate model predictions by using a Multinomial likelihood and accounting for model misfit by adding hierarchical structure elsewhere. The intercepts α_{pc} denote initial relative log prevalence of cluster c in region p; these are modeled hierarchically around the global relative log prevalence α_c of each cluster. The slopes β_{pc} are modeled hierarchically around global per-cluster fitness $\Sigma_f\ \beta_f\ X_{cf}$ that are linearly regressed against amino acid substitution features X_{cf} . These linear coefficients β_f can be directly interpreted as the effect of a mutation on a lineage's fitness, all other variation being equal. In figures we plot posterior means $\mathbb{E}[\beta_f|data]\ =: \Delta\ log\ R$ as an estimate of effect size and plot the posterior z-score $|\mathbb{E}[\beta_f|data]|\ /\ \mathbb{V}\ [\beta_f|data]^{1/2} =:\ |\mu|/\sigma$ as a proxy for statistical significance.

Note that by regressing against amino acid changes we obviate the need to directly incorporate phylogenetic information into the model: if two lineages are close together in a phylogeny, then their amino acid features are likely also similar, so their regressed fitness values will likely be similar. By sharing statistical strength in this way we are also able to make accurate predictions for emergent lineages with few observations. (Note phylogenetic information is still used in preprocessing, since our clustering is created from an UShER phylogenetic tree.) Both of the hierarchies in α and β empirically improve model fit in the presence of heavily skewed observations (e.g. most samples are from the UK, and there is a long tail of sparsely sampled regions). We chose these model structures based on extensive cross-validation and forecasting experiments.

We place weak priors on scale parameters σ_1, σ_2, and σ_4 (these denote standard deviations, the square roots of prior variance). The σ_1 and σ_2 priors are centered at large values to allow for wide variation in initial infection proportions across regions. The σ_4 prior is centered around the smaller value $e^{-4} \approx 0.018$ because we expect little variation of relative fitness across geographic regions a priori (some variation is expected, due to geographic variations in e.g. age distribution, behavior, or genetics as in binding affinity due HLA complex genotypes *(27)*). We fix the linear regression scale parameter σ_3 to a small value, forcing the regression problem towards a sparse solution (i.e. we assume a priori that most observed mutations have little effect on fitness). We choose a Laplace prior on regression coefficients because it is heavier-tailed than a Normal prior, but not so heavy-tailed that the regression problem becomes multimodal (as it would for e.g. a Cauchy or Student's t prior).

We conducted a systematic analysis to evaluate the level of L1 regularization (S30). Stronger regularization ($\sigma_3 = 0.0001$) resulted in better mutation cross-validation but resulted in a Manhattan plot that was implausibly sparse and failed to capture the known and experimentally-validated hits in the N gene. For example, the Nucleocapsid peak in the flexible linker region was truncated to positions 203-204, whereas Syed et al. *(22)* showed experimentally that mutations in positions 199 - 204 enhanced packaging (mutations above 204 were not tested). Conversely, regularization that was too weak ($\sigma_3 >= 0.001$) resulted in excess noise and poor cross-validation. An intermediate level of regularization ($\sigma_3 = 0.005$) resulted in strong cross-validation of mutation coefficients (S30A-B) and captured signals in the Manhattan plot that included experimentally-validated mutations in Nucleocapsid and Spike (S30D-E).

This proportional growth model differs from many forecasting models in the literature that are formulated in terms of absolute sample counts. Whereas our Multinomial likelihood allows us to model only the relative portions of lineages in each (time,region) bin, a Poisson likelihood would force us to additionally model the total number of genome samples in each (time,place) bin, a task which is less related to viral dynamics and more related to local lab capacity, political dynamics, and local calendars. We choose to model relative proportions rather than absolute counts because the relative model is robust to a number of sources of bias, including: sampling bias across regions (e.g. one region samples 1000x more than another); sampling bias over time (e.g. change in sampling rate over time); and change in absolute fitness of all lineages, in any (time, region) bin (e.g. due to changes in local policies or weather, as long as those changes affect all lineages equally). However the model is susceptible to the following sources of bias: biased sampling in any (time, region) cell (e.g. sequencing only in case of S-gene target failure); and changes in sampling bias within a single region over time (e.g. a country has a lab in only one city, then spins up a second lab in another distant city with different lineage proportions).

Some clusters of mutations occur together in only one or a small set of lineages, such as Omicron. If mutations are perfectly correlated across lineages, the model will score them identically, i.e. the effect size will be apportioned equally among the different mutations. However, in many cases, mutations occur independently in different numbers of lineages. In these cases PyR_0 will use this additional information to “break ties” and disentangle the effects of co-occurring mutations so that mutations that consistently emerge in multiple lineages that are highly fit will be inferred to have large effects, whereas mutations that are found in some highly fit lineages and some less-fit lineages will be inferred to have small or negligible effect sizes. The lineages in which independent mutations have emerged is listed in Supplemental Data File 2.

We interpret the regression coefficients as the relative fitness based on a well-known result in population genetics *(28)* that the change in genotype frequency in a large haploid population under selection follows a logistic curve, where the logistic growth rate parameter defines the relative fitness of genotypes.

Advantages and Limitations

This model has several advantages over existing approaches. First, it provides a principled, agnostic approach that can be applied to a large dataset to identify lineages that demonstrate concerning epidemiological features. Second, by modeling the relative fitness of lineages separately across 1560 geographic regions, the model is robust to region-specific differences in non-pharmaceutical interventions and vaccination rates. Third, the hierarchical nature of the model which represents lineages as collections of mutations draws on the underlying biology and yields both strain- and lineage-specific coefficients from a single inferential approach. Fourth, the model is fully Bayesian, and the use of regularizing priors enables us to estimate coefficients for mutations and infer estimates for all extant lineages. Finally, the model is open-source, builds on optimized numerical libraries, can be configured to run on CPUs or GPUs, and includes an option to be run without mutations.

The model also has several limitations. First, as a generalized linear regression model, the model does not explicitly incorporate higher-order (non-linear) interactions between mutations (epistasis). While the linear-additive model of mutation biology is a coarse approximation to true biology including epistasis, our hierarchical model serves as a framework to explore such models *(29, 30)* on SARS-CoV-2 genomic surveillance data. Second, the model is susceptible to sources of bias within the data. For example, preferential sequencing of SGTF lineages may bias the estimates. Third, because the model learns more from data-intensive regions, bias in such regions would have a corresponding greater impact on the resulting estimates. Fourth, the model does not incorporate migration. Thus, in order to be considered in future region-specific dynamics, a lineage must be present in a given region at the time the model is run, an effect that can be seen with BA.2 dynamics in Figure S7, i.e. BA.2 is forecast to rise only in regions where it has been detected. Inclusion of a migration model could improve long-term forecasting accuracy.

While a hierarchical model that includes mutation-level coefficients has several advantages over alternate modeling approaches, we do not believe that there is a “best” model that applies in all cases. As Supplemental Figures S6, S24, and S25 show, models at all levels of hierarchical organization and data pooling can be useful, and a systematic surveillance program should, we would argue, routinely run a diverse set of models. These might include binomial logistic regression models within a single well-sampled region, binomial logistic regression models that

jointly model several regions, multi-region multivariate logistic regression models without mutation-specific coefficients as well as PyR_0, which is a multi-region multivariate logistic regression model that incorporates mutation-specific coefficients.

Probabilistic Inference

The model is implemented in the Pyro probabilistic programming language *(16)* built on PyTorch *(31)*. To fit an approximate joint posterior distribution over all latent variables (a space of dimension 375,909), we train a flexible reparameterized variational distribution using stochastic variational inference. Our variational approach starts by reparameterizing the model via a sequence of learnable but distribution-preserving decentering transforms *(32)* on the α and β latent variables. Reparameterizing is particularly helpful in avoiding Neal's-funnel situations *(33)* by smoothing out the geometry of latent variables with Normal prior whose scale parameter is also a latent variable. After reparameterizing we model the posterior over all variables as a joint multivariate Normal distribution whose covariance matrix $\boldsymbol{\Sigma}$ is parametrized by a rank-200 matrix plus a diagonal matrix $\mathbf{D}$ with positive entries:

$$\boldsymbol{\Sigma}_{ij} = \sum_{\ell=1}^{200} \boldsymbol{\Lambda}_{i\ell} \boldsymbol{\Lambda}_{j\ell} + \boldsymbol{D}_{ij}$$

where $\boldsymbol{\Lambda}$ is an unconstrained matrix of size 375,909 x 200. This low-rank multivariate Normal distribution allows the approximate posterior to capture correlated uncertainty among competing mutations each of which might explain increased fitness. This variational distribution has 75,936,525 parameters to be optimized (much larger than the number 375,909 of latent variables, but much smaller than the 375,909 $\times$ (375,909 + 1) / 2 $\cong 7 \times 10^{10}$ parameters that would be required to represent a full-rank covariance matrix).

Variational inference is performed for 10,000 iterations with the Adam optimizer *(34)* with clipped gradients and an exponentially decreasing learning rate schedule and initial learning rates between 0.05 and 0.0025 for different parameter groups (see Figure S31). Optimization proceeds in batch-mode, i.e. without any data subsampling. We initialize model parameters to median prior values with a small amount of noise added to avoid scale parameters collapsing early in training. After inference we make predictions by drawing 1000 posterior samples. See source code for detailed optimizer and initialization configuration.

Inference and prediction on a single GPU (NVIDIA Tesla A100 with 48GB of RAM) takes about 10 minutes (compared to 14.5 hours on an 8-core CPU), which is less than the amount of time required to download and preprocess each daily snapshot of data from GISAID. The cost of fitting the model is O((TP+F)C), dominated by pointwise mathematical operations, particularly computing the softmax function on a dense array of shape T×P×C. This cost does not depend directly on the number of genetic samples, since samples are aggregated into counts y of constant shape T×P×C.

We emphasize that inference in this model is very challenging due to the large dimension of the latent space (namely 375,909), itself a consequence of the large number of regions, lineages, and mutations considered by the model *(35)*. While variational inference has a number of attractive features, especially computationally, like any approximate inference scheme it comes with disadvantages. In our case the most notable disadvantage of variational inference is its tendency to yield biased posterior uncertainty estimates. Typically posterior uncertainty is underestimated,

leading to credible intervals (CI) that in some cases can be unrealistically narrow. The primary parameters of interest in the PyR_0 model are the mutation-level coefficients β_f and the per-lineage fitness values $\Sigma_f\ \beta_f\ X_{cf}$. Since the latter quantity governs the prior over β_{pc}, which in turn directly feeds into the multinomial likelihood, the per-lineage fitness estimates are more-or-less tightly constrained by the observed counts y_{tpc}. Consequently the posterior uncertainty of per-lineage fitness is comparatively easy to estimate and we expect variational inference to yield reasonable credible intervals for these quantities. In contrast the mutation-level coefficients β_f interact with correlated features X_{cf} (leading to a multi-modal posterior) and are less directly constrained by the observed counts y_{tpc}. Consequently it is significantly more challenging to estimate the corresponding posterior uncertainty. In practice we obtain implausibly narrow credible intervals for these quantities and the posterior uncertainty must be interpreted with caution. Importantly, while the uncertainty estimates for β_f should not be taken at face value, we believe that they are still very useful for interpreting inferred model parameters, since they *can be used to rank/prioritize different hits* β_f. In particular, while the absolute magnitudes of β_f uncertainty estimates are implausible, their *relative magnitudes* are representative of the amount of supporting evidence, and thus are useful for ranking. Since we consider a large number of mutations (F=2904) this information is invaluable for designing experiments for functional characterization.

Implementation

We implemented the PyR_0 model using the probabilistic programming language Pyro *(16)*. The model leverages PyTorch and Pyro to scale efficiently to large data sets and can therefore be applied continuously as datasets grow, completing model training and prediction with millions of viral genomes in minutes on a single GPU. We chose the Pyro framework because it cleanly separates model specification from inference customization, and scales to large models and datasets by leveraging GPUs. This flexible modeling framework allowed us to experiment with different hierarchical structures. Additionally by relying on an open source and well-tested modeling and inference framework, we minimize the risk of introducing software bugs into our analysis. The speed of inference—which took about 10 minutes on a single GPU on the full dataset of >6 million genomes—allowed quick model iteration and thorough validation on subsets of the data, including both geographic cross-validation and temporal data truncation.

Prediction

In Figure 1, the 95% confidence intervals in parentheses were estimated by drawing 1000 samples from the variational posterior distribution. Confirmed cases per day were estimated at the end of the training period (Jan 20 2021) by combining our model's relative lineage portions with confirmed case count data from Johns Hopkins university. Quantities defined over our 3000 fine clusters were aggregated up to coarser PANGO lineages for reporting. To facilitate downstream use of model predictions we have provided complete tables of lineage fitness estimates (Data S1) and mutation coefficients (Data S2). These predictions have been used e.g. by Nextstrain.org to visualize our predicted mutational fitness along a phylogenetic tree (Figure S32).

Validation

We considered the possibility of biased submission to the GISAID database and compared results obtained from the full dataset with results obtained from disjoint subsets. For this purpose we divided the data into samples from the most heavily sampled region (Europe, with 3.3M

samples) and those from the rest of the world (with 3.1M samples) (Figures S1,S11). This split is motivated by most samples originating from the UK: we widened the region around the UK until the region and its complement both had roughly equivalent statistical strength and narrow posterior estimates. We conducted two-fold cross-validation experiments for both lineages (Figure S2) and mutations (Figure S19). Additionally, in Figure S33, we show that PyR_0 lineage-level Δ log R estimates are largely driven by regions with the largest numbers of samples and are thus robust to the manner in which under-sampled regions are organized into spatial units.

We found the full GISAID dataset to be invaluable to making accurate predictions. Using data up to July 2021, we tried restricting to either all CDC data or CDC's randomly sampled NS3 dataset and found those subsets to result in insufficient diversity and lead to unclear results (Pearson correlation 0.49, 0.28, respectively). Using data snapshots from mid January 2022, we tried restricting to open data available in GENBANK, but found the model made implausible estimates of Omicron fitness, due to a combination of lack of geographic diversity (GENBANK has only about 1/10 as many geographic regions as we were able to extract from GISAID data, and particularly has very few samples from South Africa) and data upload latency (GISAID appeared to have ~1 week upload latency, versus ~1 month for GENBANK).

To evaluate predictions based on sequence alone, we conducted a leave-one-out (LOO) analysis in which each lineage's fitness was estimated from its mutational profile after: i) training on the full dataset; and ii) training on the full dataset with each lineage and its subclade (all descendents) removed (Figure S17). We compared these LOO estimates to a baseline estimator in which each child lineage's fitness is the same as its parent.

Our model assumes each single point mutation independently linearly contributes to change in fitness. A natural generalization is to search for groups of mutations that affect fitness. To explore this we fit a similar model of both single and pair mutations, considering only pairs that lie within the same gene. Fitting this model on data up to July 2021, we discovered no pairwise mutations stronger than the top 100 single mutations. While these experiments did not discover pairwise mutations, we believe that more sophisticated models would be able to measure epistasis, but sophistication in that area is beyond the scope of the present work.

We also compared our inference of lineages and mutations from the current model, run on 6.4 million genomes, downloaded on January 20, 2022, to an earlier version of the model (see methods) run on 2.1 million genomes, downloaded on July 6 2021*(17)*. The lineage-level estimates replicate well (Spearman's rho = 0.78, $p < 2.2 \times 10^{-16}$). Mutation-level estimates are also significantly correlated (rho = 0.48, $p < 2.2 \times 10^{-16}$) but the global correlation is weaker, as expected given the difficulty of the inference task and the incorporation of millions of new genomes in the post-Omicron model. We note that the mutation-level correlation is strongest in Spike (rho = 0.62, $p < 2.2 \times 10^{-16}$) and N (rho = 0.62, $p < 2.2 \times 10^{-16}$) (S15E), where the signals appear strongest and the greatest amount of experimental validation exists.

Finally, to compare our assessments of lineage fitness to other logistic regression approaches, we compared lineage fitness estimates for PyR_0 without mutations (Figure S11), multinomial logistic regression (Figure S12), and binomial logistic growth curves (S13), showing good agreement across all approaches.

Supplemental Note 2:

Cell culture
Cells were cultured in humidified incubators with 5% CO_2 at 37º C, and monitored for mycoplasma contamination using the Mycoplasma Detection kit (Lonza LT07-318). HEK293 *Homo sapiens*, female, embryonic kidney cells (ATCC CRL-1573) were cultured in DMEM supplemented with 10% heat-inactivated FBS, 1 mM sodium pyruvate, 20 mM GlutaMAX, 1× MEM non-essential amino acids, and 25 mM HEPES, pH 7.2.

Virus production
24 hrs prior to transfection, 6×10^5 HEK-293 cells were plated per well in 6 well plates. All transfections used 2.49 µg plasmid DNA with 6.25 µL TransIT LT1 transfection reagent (Mirus, Madison, WI) in 250 µL Opti-MEM (Gibco). Single-cycle HIV-1 vectors pseudotyped with SARS-CoV-2 Spike protein, either D614 or D614G, were produced by transfection of either HIV-1 pNL4-3 Δenv Δvpr luciferase reporter plasmid (pNL4-3.Luc.R-E-), or pUC57mini NL4-3 Δenv eGFP reporter plasmid, in combination with the indicated Spike expression plasmid, at a ratio of 4:1. ACE2 expression vectors were produced by transfecting cells with one of the pscALPSpuro-ACE2 plasmids, along with the HIV-1 *gag-pol* expression plasmid psPAX2, and the VSV glycoprotein expression plasmid pMD2.G (4:3:1 ratio of plasmids). 16 hrs post-transfection, culture media was changed. Viral supernatant was harvested 48 hours after media change, passed through a 0.45 µm filter, and stored at 4ºC. TMPRSS2 expression transfer vector was produced similarly but with pscALPSblasti-TMPRSS2.

Generation of cell lines expressing ACE2 and TMPRSS2
2.5×10^5 HEK-293 cells were plated per well in a 12 well plate. The next day cells were transduced with 250 uL of supernatant containing TMPRSS2-encoding lentivirus for 16 hr at 37°C, after which fresh media was added to cells. 48 hrs after transduction cells were replated and selected with blasticidin (InvivoGen, catalogue #ant-bl-1) at 10 ug/ml. After selection, cells were transduced similarly with supernatant containing ACE2-encoding lentivirus and selected with 1 ug/mL of puromycin (InvivoGen, San Diego, CA, catalogue #ant-pr-1).

Virus Infectivity Assays
16 hours prior to transduction, adherent cells were seeded in 96 well plates. HEK-293 cells were plated at 5×10^4 cells per well. Cells were incubated in virus-containing media for 16 hrs at 37°C when fresh medium was added to cells. 48 to 72 hours after transduction cells were assessed for luciferase activity. Cells transduced with luciferase expressing virus were assessed using Promega Steady-Glo system (Promega Madison, WI). GraphPad Prism 8.4.3 was used to analyze the infectivity data using a ratio paired t test. In these experiments, all values shown are the mean with standard deviation, with the actual calculated two-tailed P value indicated.

Supplemental Note 3:

We include here an extended discussion of high-scoring mutations.

Spike

Many of the high-scoring mutations in our model were from Spike. As discussed in the main text, fitness-associated Spike mutations tended to cluster in hotspots in NTD, RBD, and furin-cleave domain. For Omicron, antibody escape appears to be the dominant mechanism as predicted by our model (Figure 3B, S25B) and shown experimentally *(36, 37)*, across all variants the mechanisms by which these mutations enhance growth likely include enhancing infectivity, promoting cell-cell fusion, and increasing immune escape beyond humoral immunity, and other mechanisms. The mutational fitness of individual mutations was strongly between the 2.1 million genomes model and the 6.4 million genomes model (rho = 0.62, $p < 2 \times 10^{-16}$, Figure S15F), and includes many mutations that have been experimentally characterized including those at positions E484, L452, T478, and N501. Some of these (e.g. E484K) have been shown to promote escape from neutralizing antibodies (*(38)*) whereas others (N501Y, N501T) have been shown to enhance binding to ACE2 *(9)*. While T478K is the among top-scoring Spike mutations in our dataset, we are not yet aware of any definitive experimental results that would explain a potential fitness for this mutation.

Spike D614G was highly ranked by our initial model but appeared to have a neutral effect in the 6.4 million genomes model, a situation that also affected ORF1b P314L, a mutation that is almost perfectly linked to Spike D614G. We attribute this to effective fixation during the follow-up period, as nearly all variants of concern and all genomes sequenced since July 2020 contain these two mutations (Figure S34).

Nucleocapsid

The concentration of putative transmission-promoting substitutions in N at positions 160-210 is remarkable, but is supported by a similar observation in Ebola virus*(39)*, and recent data for SARS-CoV-2 showing mutations in that region increase the efficiency of viral packaging*(40)*, validating some of the model's most unexpected predictions and supporting its ability to identify novel biology.

ORF1

Our model highlighted mutations within the ORF1 non-structural proteins (nsps) whose functions are not fully understood (e.g. Table S3). We found two predominant clusters within ORF1a: one in the C-terminal ~120 amino acids of nsp4 and the other within the N-terminal ~160 amino acids of nsp6 (Figure S13C). Nsp4 and nsp6 are both membrane-anchored proteins with roles in assembly and concentration of the viral replication and transcription complex (RTC) machinery within double-membrane vesicles *(41)*. Amino acid substitutions in these regions, combined with transmission-associated mutations identified within additional RTC-associated nsps (e.g., nsp12-16, Figure S13D), may therefore affect the kinetics of replication and gene expression, resulting in higher virus yields from infected cells. Nsp2, a rapidly evolving accessory protein *(42)(43)(44)* whose proposed function in disrupting host cell signaling *(45)* and viral mRNA translation initiation *(46)* remains obscure, harbored many additional mutations associated with higher fitness (Figure S13C).

The ORF1a-ORF1b polyprotein is processed into 16 non-structural proteins by two viral proteases: a papain-like protease (nsp3) and 3C-like protease (nsp5). Multiple transmission-associated mutations were found within the protease coding regions (e.g., ORF1a:V1750A, ORF1a:P3395H). Most of the amino acid substitutions identified by our model were outside of the domains containing catalytic residues for nsp3 (C1674, H1835, D1849) or nsp5 (H3304, C3408) *(47)(48)*. However, the potential effects of these mutations on protease architecture and activity warrant further experimentation. A few of the top mutations from our model (e.g., ORF1a:T3255I, ORF1a:A3571V) are positioned adjacent to nsp cleavage sites, potentially influencing local structures and kinetics of polyprotein processing by nsp3 and nsp5 (Figure S13C-D).

Multiple highly-ranked mutations are distributed across the replication and transcription-associated nsps in ORF1b (Figure S13D). The P314L mutation in nsp12 – the viral RNA-dependent RNA polymerase (RdRP) – emerged early during the pandemic and became established in circulating lineages alongside S D614G *(7)*. And like D614G, P314L was highly ranked by our initial model but had an approximately neutral effect in the 6.4 million genomes model, presumably due to fixation of this mutation during the follow-up period. A later variant at this site (P314F) was also highly ranked in our list. Additional mutations in nsp12 can be found within the canonical fingers (D445A, V631I, D514N, G662S), palm (M592I, H604Y, T701I, C721R, S763F), and thumb (L820F, L829I, D870N) subdomains of the RdRP conserved catalytic fold (Figure S15). The functional effects of these mutations on polymerase processivity and fidelity remain to be investigated. A structural model of the SARS-CoV-2 polymerase complex has been resolved *(49)(50)*, and contains a single subunit of nsp12, two subunits of the nsp13 helicase, and additional RdRP cofactor proteins (nsp7, 8, and 9). The ORF1b P314 residue is located at the interaction interface between nsp12 and a single subunit of nsp8. Moreover, several of the top mutations from our dataset ORF1b (e.g., P1000L, P1001S, Q1011H) are harbored within the nsp13 N-terminal zinc-binding domain that directly interacts with nsp8 *(51)*. These findings implicate transmission-associated mutations within the SARS-CoV-2 RNA synthesis machinery in altering the stability of the replication complex, possibly via interactions with nsp8.

Nsp14 is a dual-functional enzyme with N-terminal 3’-to-5’ exonuclease (ExoN) and C-terminal guanine-N7 methyltransferase (N7-MTase) activities *(52)* and is a core component of the coronavirus RNA proofreading complex. Nsp14 is uniquely responsible for excision of mismatched bases from the nascent RNA and methylation of the viral mRNA cap structure. Two mutational hotspots in nsp14 map to discrete regions in the ExoN (e.g., T1540I, I1566V) and N7-MTase (e.g., D1848Y, P1936H) domains. The functional consequences of these clusters of transmission-associated mutations on mRNA synthesis and genome replication remain unknown.

Supplementary Figures

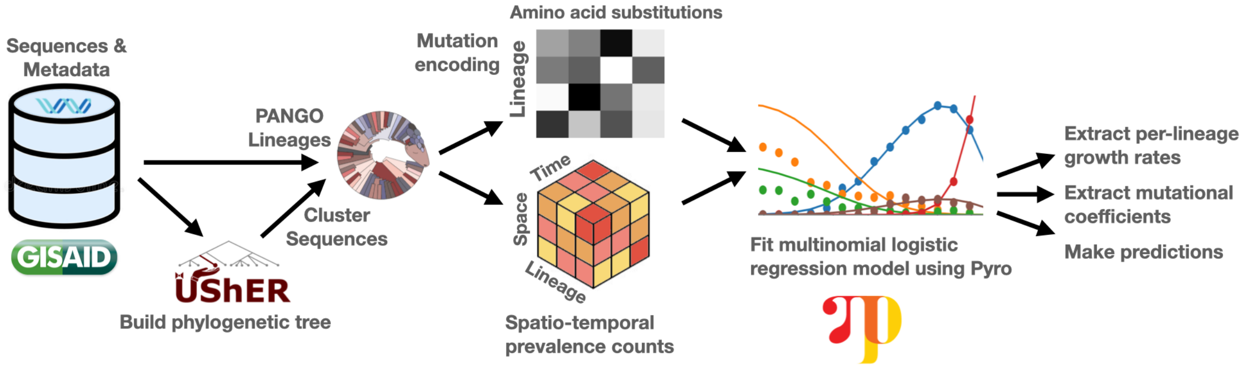

Figure S1. Overview of the PyR$_0$ analysis pipeline. After clustering UShER's mutation annotated tree, sequence data are used to construct spatio-temporal lineage prevalence counts y_{tpc} and amino acid substitution covariates X_{cf}. Pyro *(16)* is used to fit a Bayesian multivariate logistic multinomial regression model to y_{tpc} and X_{cf}.

A.

B.

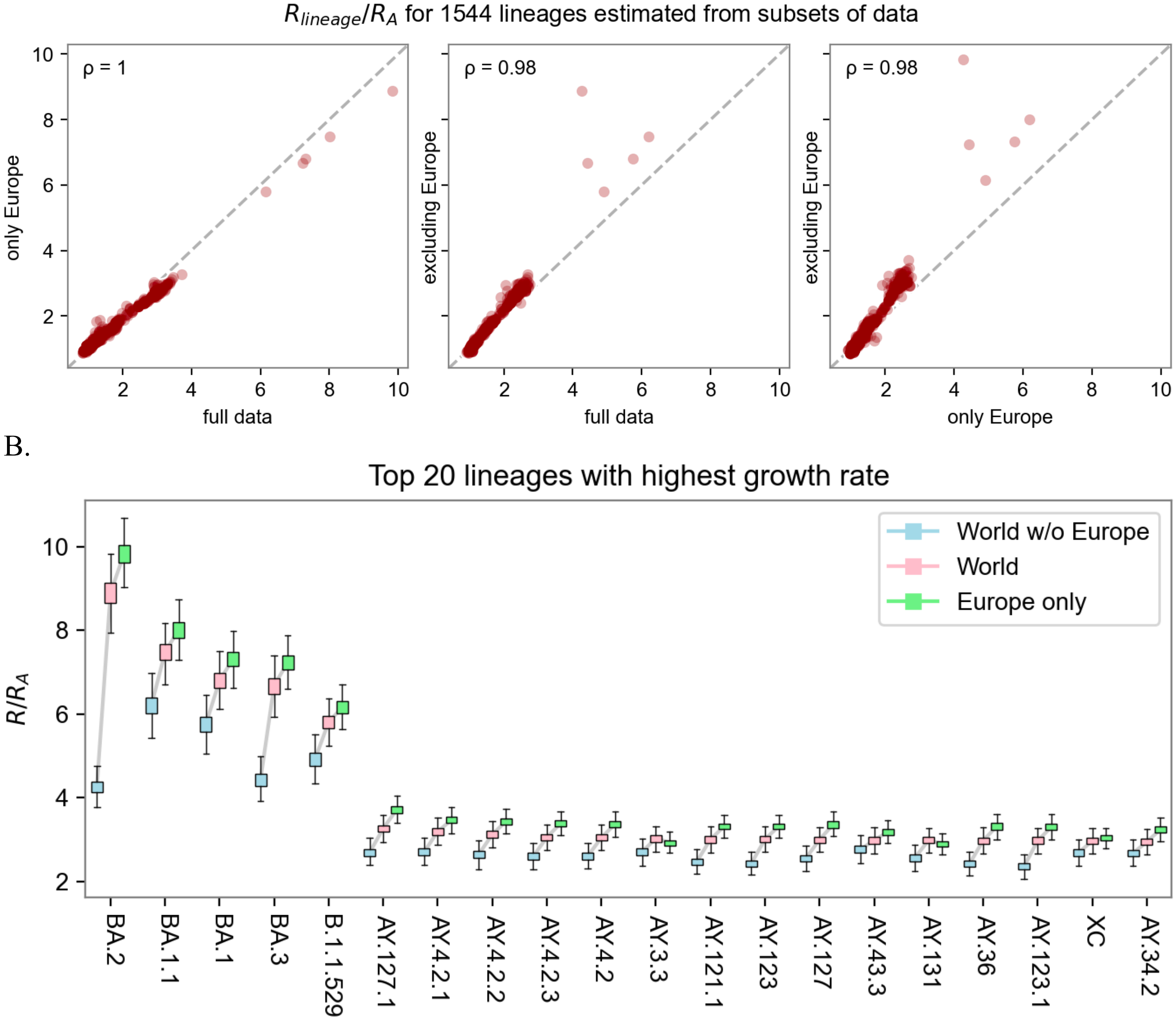

Figure S2. A. Sensitivity of lineage fitness estimates to data subset. We depict the relative fitness of all lineages as estimated by either the full data or two disjoint geographic subsets (within Europe and outside Europe). High Pearson correlation (ρ) suggests estimates are largely insensitive to data subset. **B**. Estimates of fold increases in fitness for the top 20 lineages. Sensitivity analysis shows consistency across estimates from subsets of the data in different geographic regions.

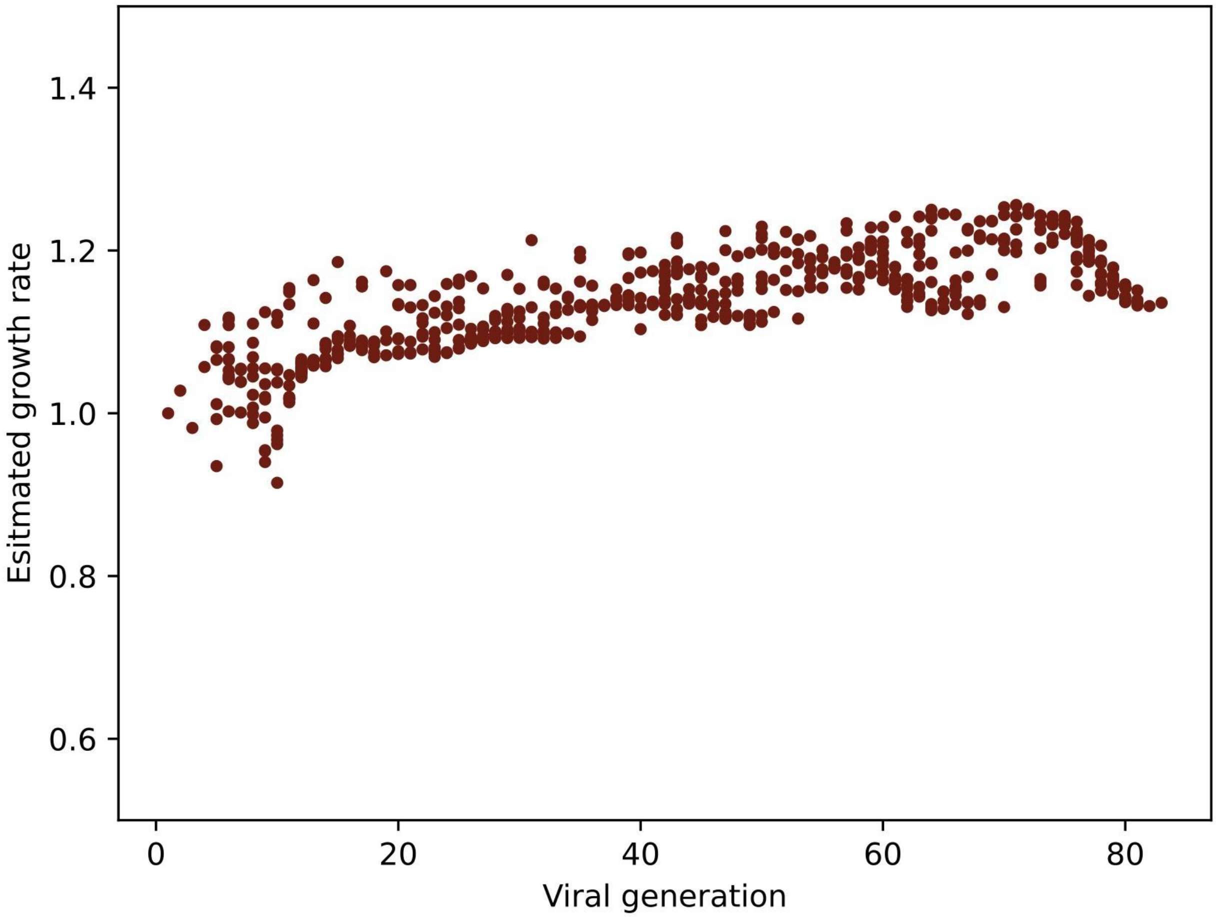

Figure S3. Simulation study assessing bias. Distribution of inferred fitness of new lineages as a function of time, for a simulated neutrally evolving viral population. The most successful subclades of each generation are designated as new lineages, leading to a trend toward higher estimated fitness even though all lineages are equally transmissible.

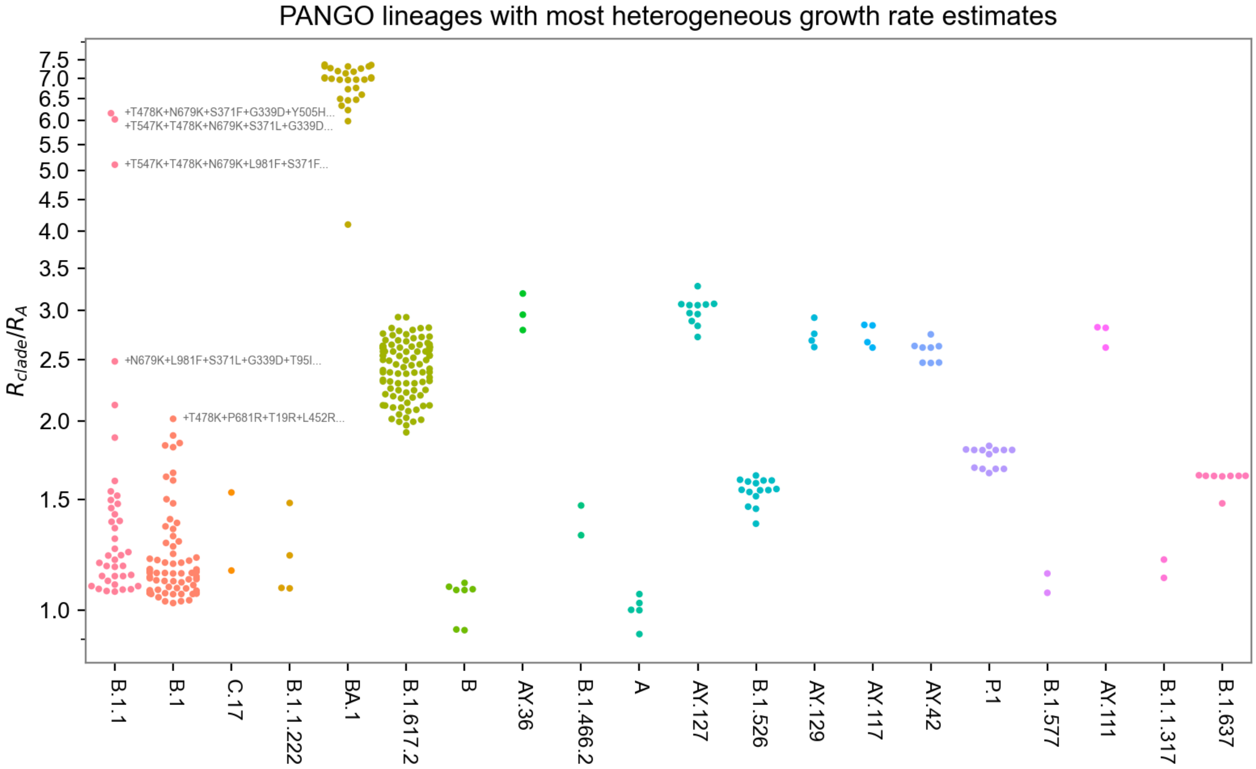

Figure S4. Heterogeneity of PANGO lineages. We hypothesized that the PANGO lineage clustering conflated viruses with distinct growth rate, e.g. B.1.1 exhibits two peaks in relative abundance in England, contrary to our multivariate logistic model. To test this hypothesis we refined 1544 PANGO lineages into 3000 finer clusters and estimated each cluster's growth rate. As shown in the figure some PANGO lineages include clusters with estimated fitness differing by more than a factor of 6, including the B.1.1 lineage. This heterogeneity is also reflected in the temporal structure: for example, the three B.1.1 clusters with the largest growth rate emerged in December 2021 and January 2022, whereas the majority of B.1.1 clusters emerged in the twelve months leading up to April 2021. The top four clusters in B.1.1 and the top cluster in B.1 are labeled by their top 5 fitness-increasing mutations to the S gene, relative to the PANGO lineage's basal sequence. PANGO lineages represented by only a single cluster (e.g. B.1.1.529, BA.2) do not have a within-lineage heterogeneity we can assess and are not shown.

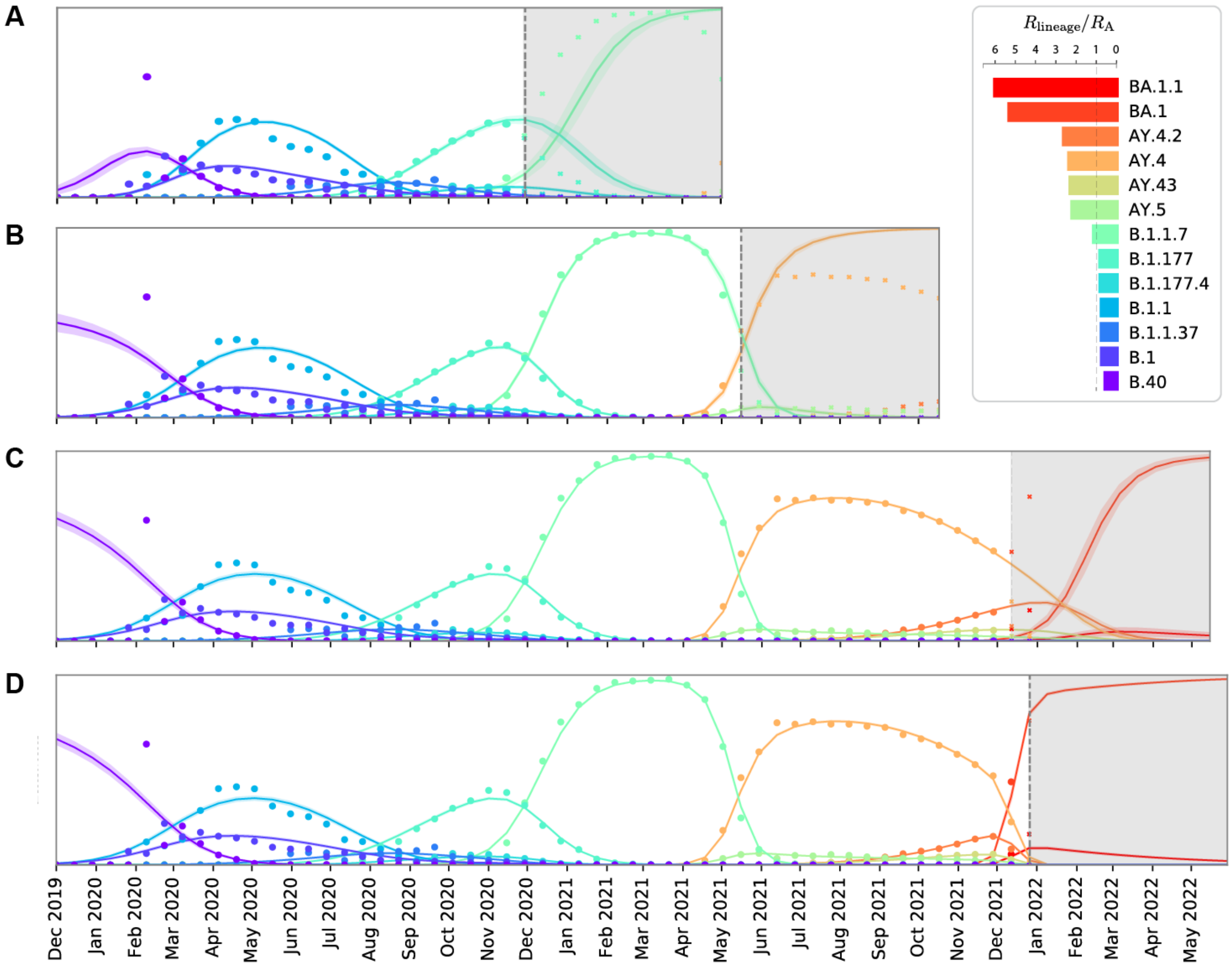

Figure S5. Forecasts in England with time-truncated input data. (A) Prediction for rise of B.1.1.7 using data through late November 2020 (solid circles at the beginning of each two-week time interval). (B) Prediction for rise of AY.4 using data through early May 2021. (C) Prediction for rise of BA.1 using data through mid December 2021, and (D) late December 2021. Future data points, not used during the model training, are shown in crosses. The legend reports lineage fitness estimates based on all available data.

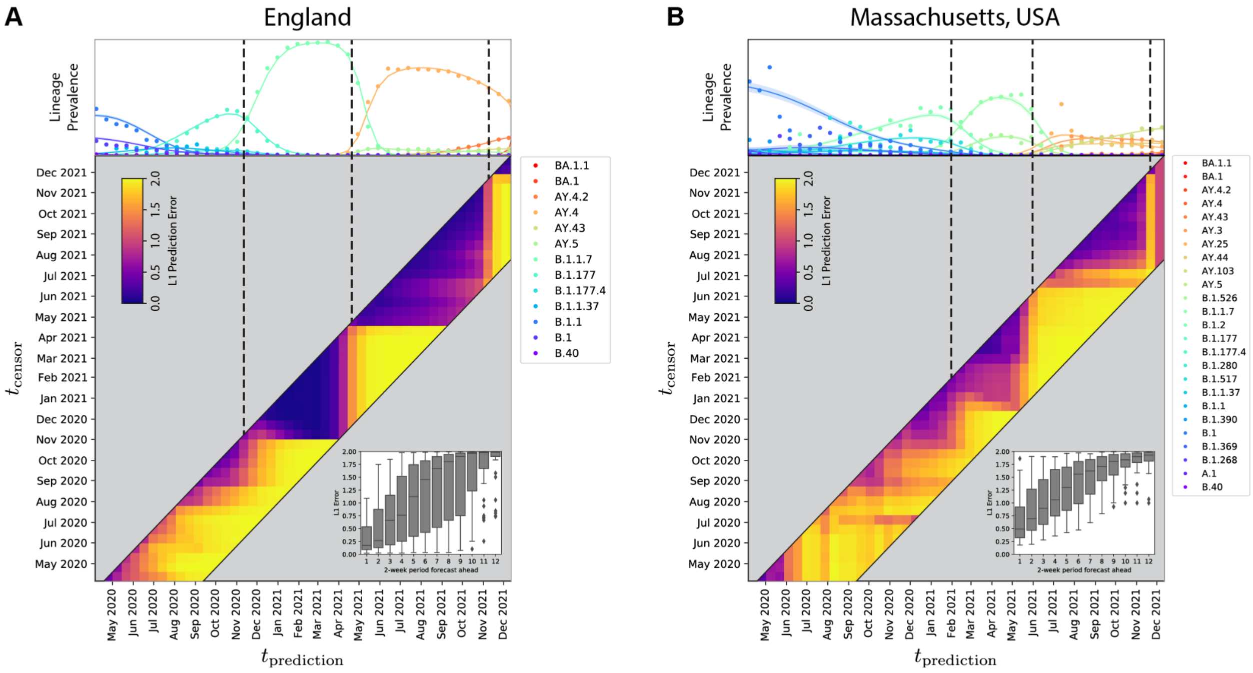

Figure S6: Forecasting evaluation based on independently trained models at 45 time points during the pandemic, t_{censor}, and predicting at time $t_{predicted}$ up to 12 two-week periods into the future. The results are shown for (A) England and (B) Massachusetts, USA. The top panels are as in Figure S3, heatmaps depict the prediction L1 error, and the inset bar plots depict the aggregated prediction errors over all periods. Note the rapid increase in error as new fit lineages emerge in a region (vertical dashed lines provided as a guide to the eye), followed by rapid recovery and stabilization of forecasting accuracy within only a single period, highlighting the predictive value of PyR_0 for detecting variants of concern. Refer to Table S1 for tabulated forecasting accuracy figures in several other regions.

A.

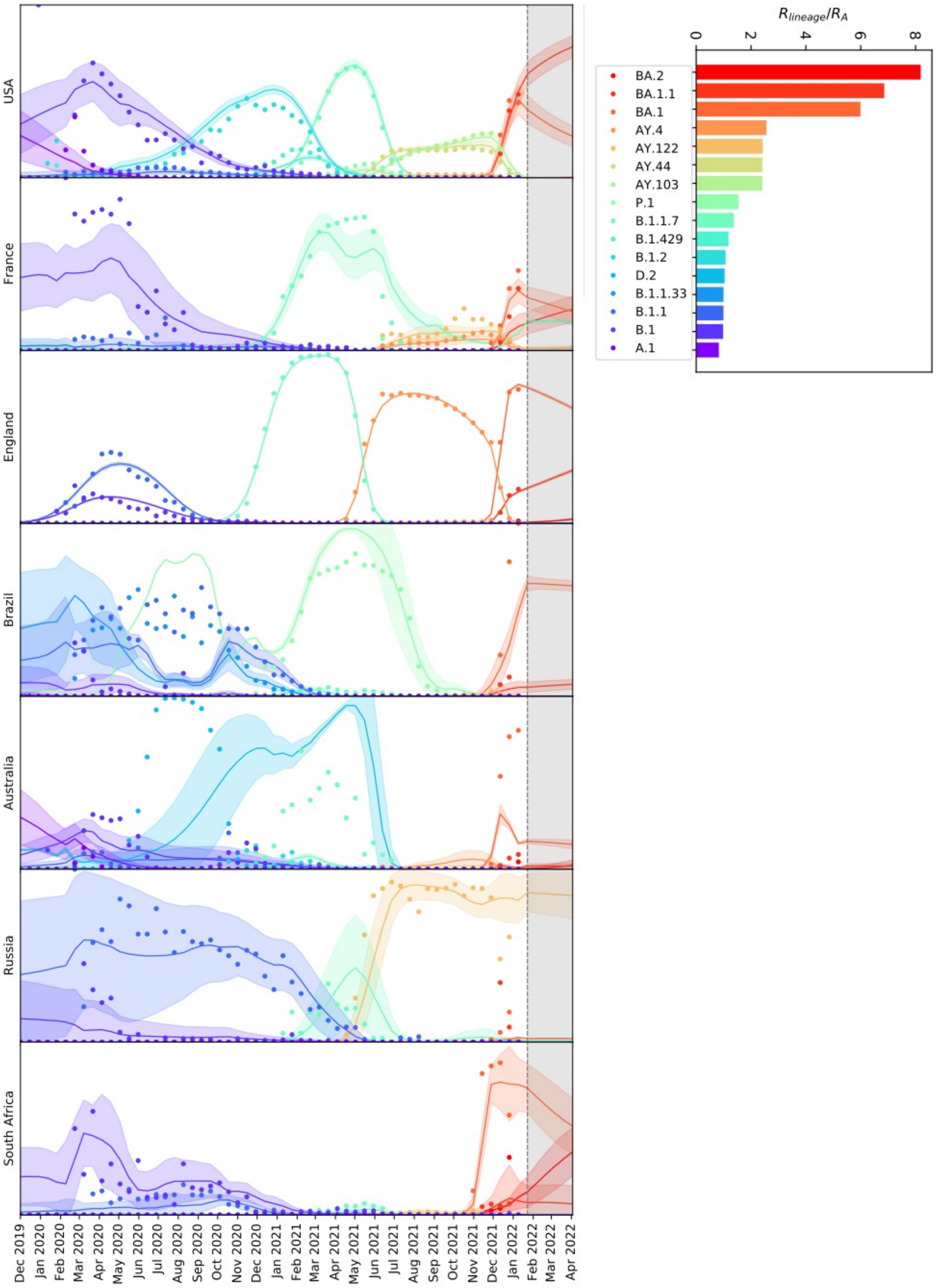
USA
France
England
Brazil
Australia
Russia
South Africa
$R_{lineage}/R_A$
0
2
4
6
8
BA.2
BA.1.1
BA.1
AY.4
AY.122
AY.44
AY.103
P.1
B.1.1.7
B.1.429
B.1.2
D.2
B.1.1.33
B.1.1
B.1
A.1
Dec 2019
Jan 2020
Feb 2020
Mar 2020
Apr 2020
May 2020
Jun 2020
Jul 2020
Aug 2020
Sep 2020
Oct 2020
Nov 2020
Dec 2020
Jan 2021
Feb 2021
Mar 2021
Apr 2021
May 2021
Jun 2021
Jul 2021
Aug 2021
Sep 2021
Oct 2021
Nov 2021
Dec 2021
Jan 2022
Feb 2022
Mar 2022
Apr 2022

B.

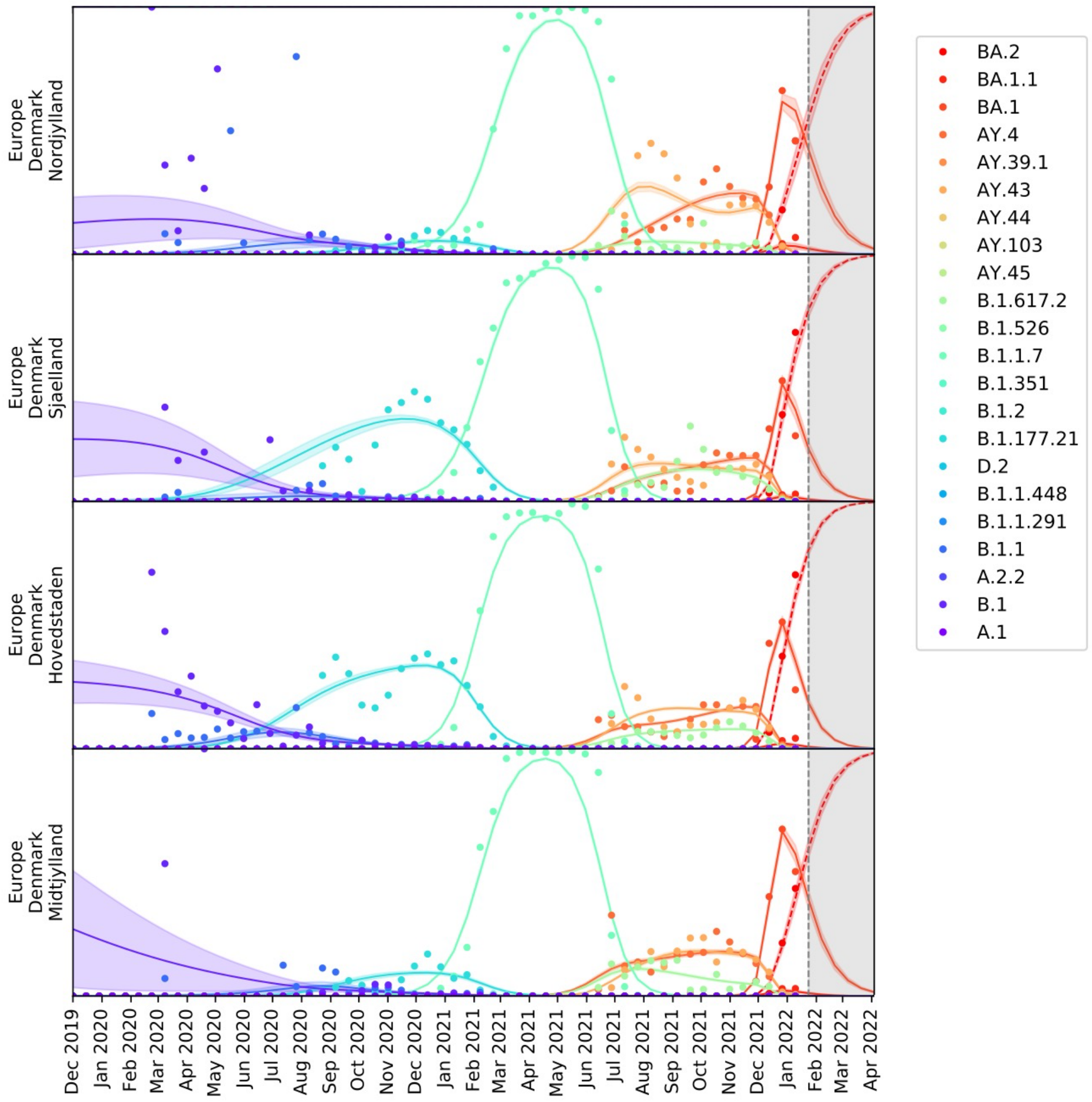
Europe
Denmark
Nordjylland
Europe
Denmark
Sjaelland
Europe
Denmark
Hovedstaden
Europe
Denmark
Midtjylland
Dec 2019
Jan 2020
Feb 2020
Mar 2020
Apr 2020
May 2020
Jun 2020
Jul 2020
Aug 2020
Sep 2020
Oct 2020
Nov 2020
Dec 2020
Jan 2021
Feb 2021
Mar 2021
Apr 2021
May 2021
Jun 2021
Jul 2021
Aug 2021
Sep 2021
Oct 2021
Nov 2021
Dec 2021
Jan 2022
Feb 2022
Mar 2022
Apr 2022
BA.2
BA.1.1
BA.1
AY.4
AY.39.1
AY.43
AY.44
AY.103
AY.45
B.1.617.2
B.1.526
B.1.1.7
B.1.351
B.1.2
B.1.177.21
D.2
B.1.1.448
B.1.1.291
B.1.1
A.2.2
B.1
A.1

C.

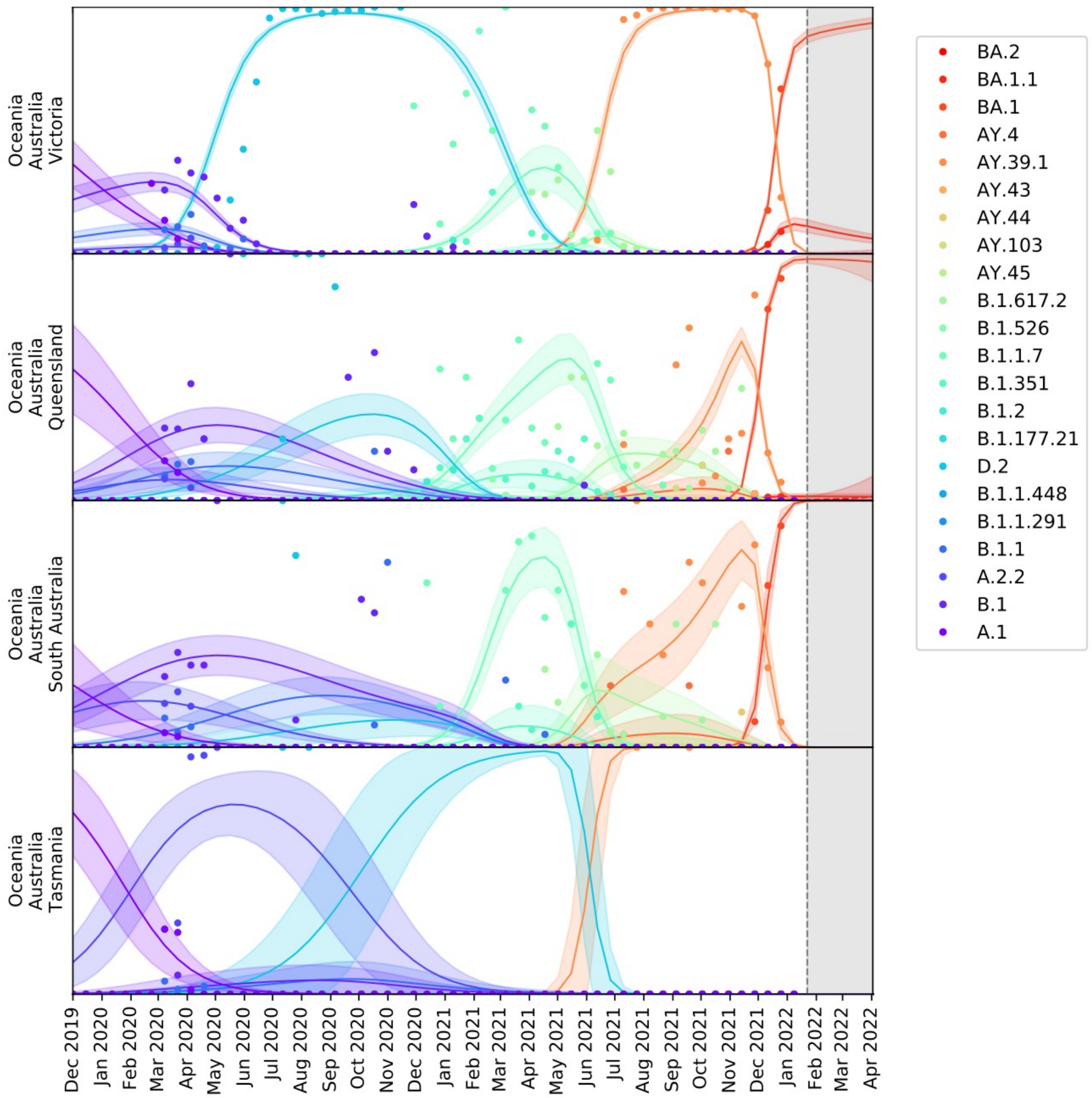
Oceania
Australia
Victoria
Oceania
Australia
Queensland
Oceania
Australia
South Australia
Oceania
Australia
Tasmania
Dec 2019
Jan 2020
Feb 2020
Mar 2020
Apr 2020
May 2020
Jun 2020
Jul 2020
Aug 2020
Sep 2020
Oct 2020
Nov 2020
Dec 2020
Jan 2021
Feb 2021
Mar 2021
Apr 2021
May 2021
Jun 2021
Jul 2021
Aug 2021
Sep 2021
Oct 2021
Nov 2021
Dec 2021
Jan 2022
Feb 2022
Mar 2022
Apr 2022
BA.2
BA.1.1
BA.1
AY.4
AY.39.1
AY.43
AY.44
AY.103
AY.45
B.1.617.2
B.1.526
B.1.1.7
B.1.351
B.1.2
B.1.177.21
D.2
B.1.1.448
B.1.1.291
B.1.1
A.2.2
B.1
A.1

D.

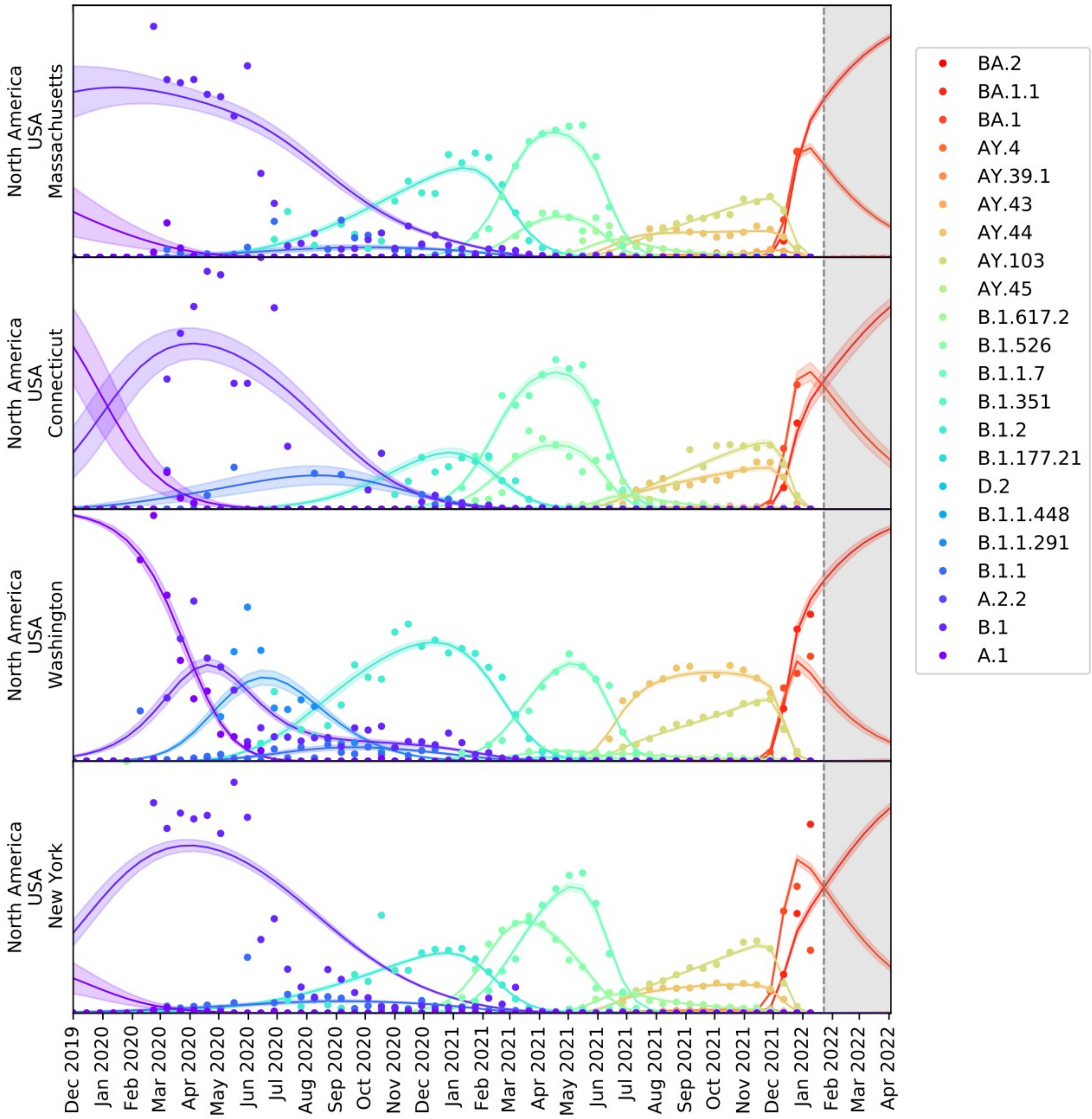

North America
USA
Massachusetts
North America
USA
Connecticut
North America
USA
Washington
North America
USA
New York
Dec 2019
Jan 2020
Feb 2020
Mar 2020
Apr 2020
May 2020
Jun 2020
Jul 2020
Aug 2020
Sep 2020
Oct 2020
Nov 2020
Dec 2020
Jan 2021
Feb 2021
Mar 2021
Apr 2021
May 2021
Jun 2021
Jul 2021
Aug 2021
Sep 2021
Oct 2021
Nov 2021
Dec 2021
Jan 2022
Feb 2022
Mar 2022
Apr 2022
BA.2
BA.1.1
BA.1
AY.4
AY.39.1
AY.43
AY.44
AY.103
AY.45
B.1.617.2
B.1.526
B.1.1.7
B.1.351
B.1.2
B.1.177.21
D.2
B.1.1.448
B.1.1.291
B.1.1
A.2.2
B.1
A.1

E.

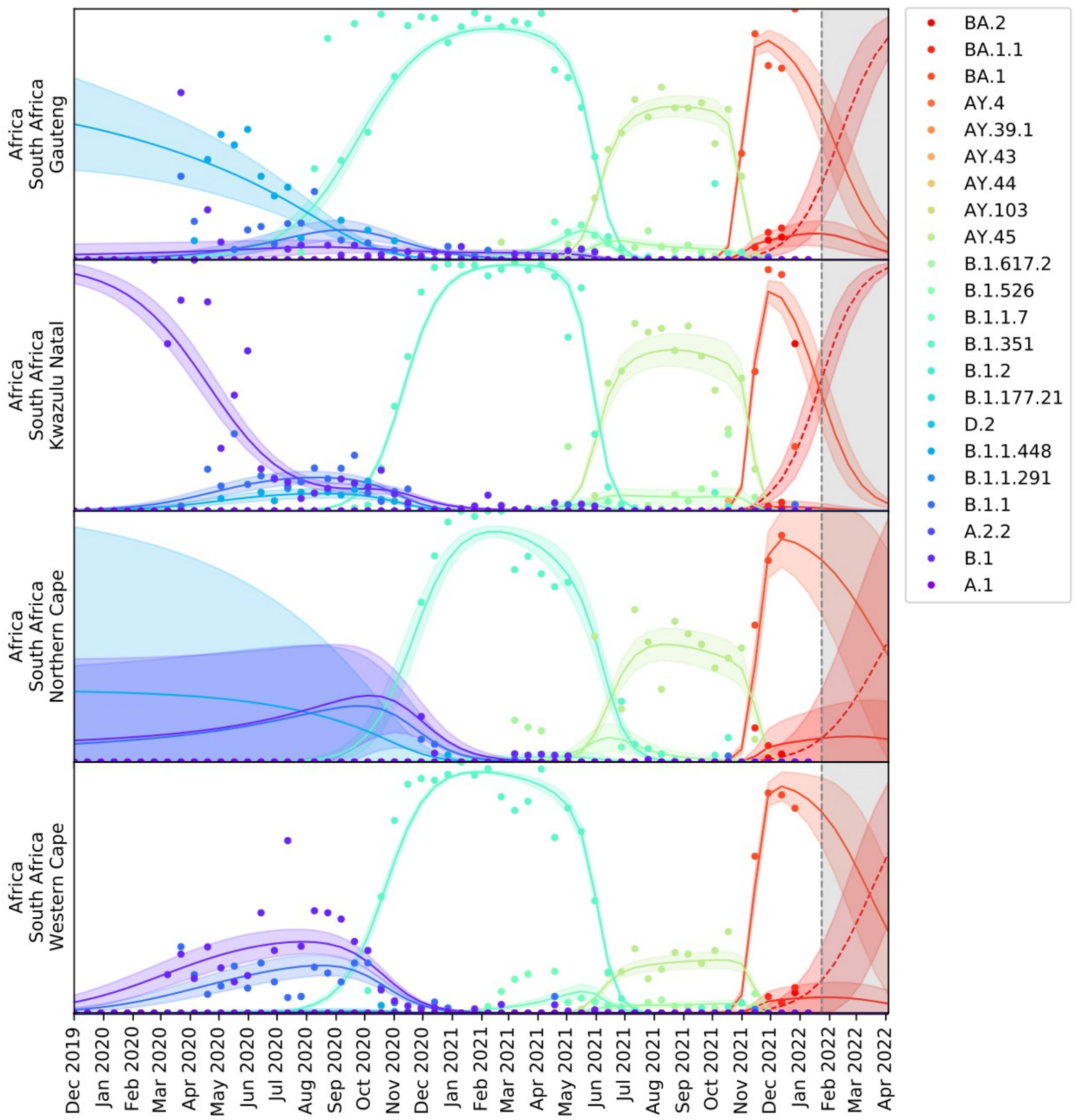

Africa
South Africa
Gauteng
Africa
South Africa
Kwazulu Natal
Africa
South Africa
Northern Cape
Africa
South Africa
Western Cape
Dec 2019
Jan 2020
Feb 2020
Mar 2020
Apr 2020
May 2020
Jun 2020
Jul 2020
Aug 2020
Sep 2020
Oct 2020
Nov 2020
Dec 2020
Jan 2021
Feb 2021
Mar 2021
Apr 2021
May 2021
Jun 2021
Jul 2021
Aug 2021
Sep 2021
Oct 2021
Nov 2021
Dec 2021
Jan 2022
Feb 2022
Mar 2022
Apr 2022
BA.2
BA.1.1
BA.1
AY.4
AY.39.1
AY.43
AY.44
AY.103
AY.45
B.1.617.2
B.1.526
B.1.1.7
B.1.351
B.1.2
B.1.177.21
D.2
B.1.1.448
B.1.1.291
B.1.1
A.2.2
B.1
A.1

Figure S7. **A.** Regional fits and forecasts for USA, France, England, Brazil, Australia, ~~and~~ Russia, and South Africa. Solid circles at the beginning of each two week time interval denote observed lineage proportions on a [0, 1] scale for the top 20 lineages. Solid curves and 95% confidence bands denote model predictions and three-month forecasts. Each of the seven (aggregate) regions is made up of multiple subregions. The behavior of each SARS-Cov-2 cluster in each subregion is represented by only two numbers in the model: a slope and an intercept. The complex model fit results from the multivariate logistic function applied jointly to multiple competing trends, which are then aggregated over subregions and multiple clusters per lineage. England shows clear waves of dominance: B.1.1, B.1.177, B.1.1.7 (Alpha), AY.4 (Delta), and finally BA.1 (Omicron), with the latter currently being overtaken by BA.1.1 (also Omicron). Massachusetts and Brazil both start with very low sampling rates early in the pandemic. The legend reports the estimated fitness for the top 16 lineages. Aggregation across regions involves forecasting probabilities to counts of given lineages (merging case data and modeled probabilities), introducing additional uncertainty that is outside the model. Thus subregion-specific fits are valuable and reflect the fit of the model to the input data: **B-E.** Subregion-specific fits for several countries across the world, including Denmark (**B**), Australia (**C**), USA (**D**), and South Africa (**E**), demonstrating better fits in regions with high sampling (USA, Denmark) and degraded fits in regions with low sampling (Australia / Tasmania). In panels **B-E**, BA.2 is annotated with a dashed line.

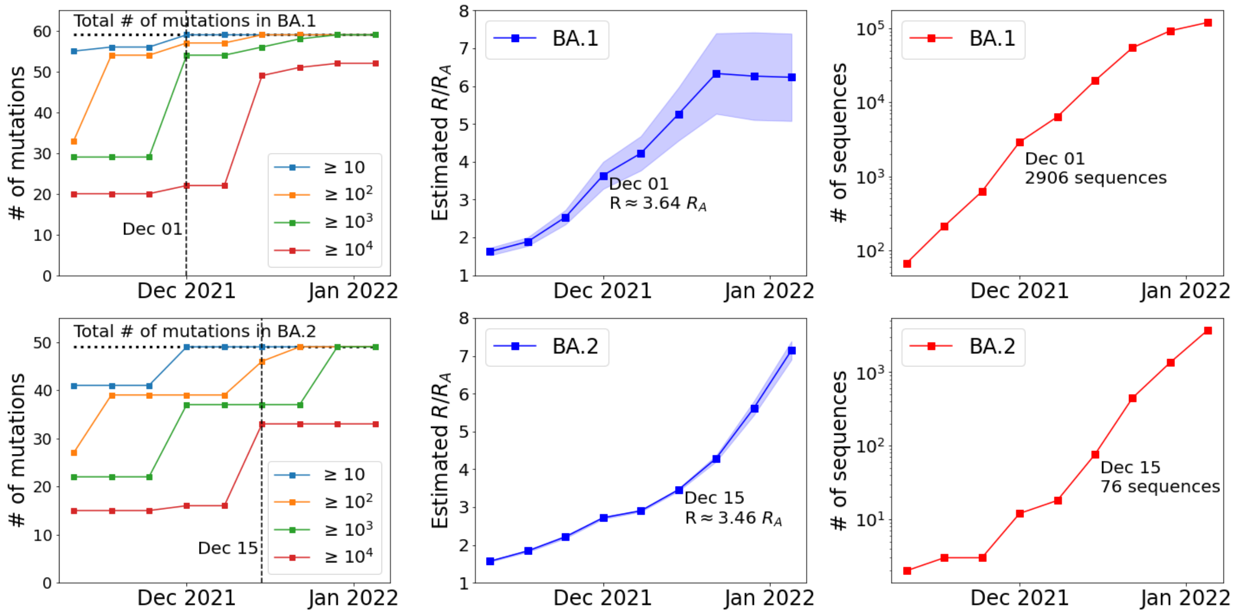

Figure S8. We depict the ability of PyR$_0$ to predict the fitness of Omicron sublineages BA.1 and BA.2 as the number of sequenced genomes increased throughout the last two months of 2021. The first time interval in which the estimated R/R$_A$ surpassed 3.29, the upper 95% CI of the fittest Delta lineage (AY.127.1), is marked. PyR$_0$ predicted that BA.1 (respectively, BA.2) was more fit than Delta by December 1st (15th) 2021, by which time 2906 (76) genomic sequences had been collected. The substantial heterogeneity of the BA.1 sublineage is reflected in the uncertainty in BA.1 R estimates; this heterogeneity also helps explain why PyR$_0$ required more sequences to identify the elevated fitness of BA.1 as compared to the case of BA.2. **Left:** The number of amino acid mutations that make up BA.1 and BA.2 that had been observed in at least 10/10^2/10^3/10^4 sequences by the given date. **Middle:** Estimates of R/R$_A$ using sequences collected by the given date. **Right:** The total number of BA.1 and BA.2 sequences collected by the given date.

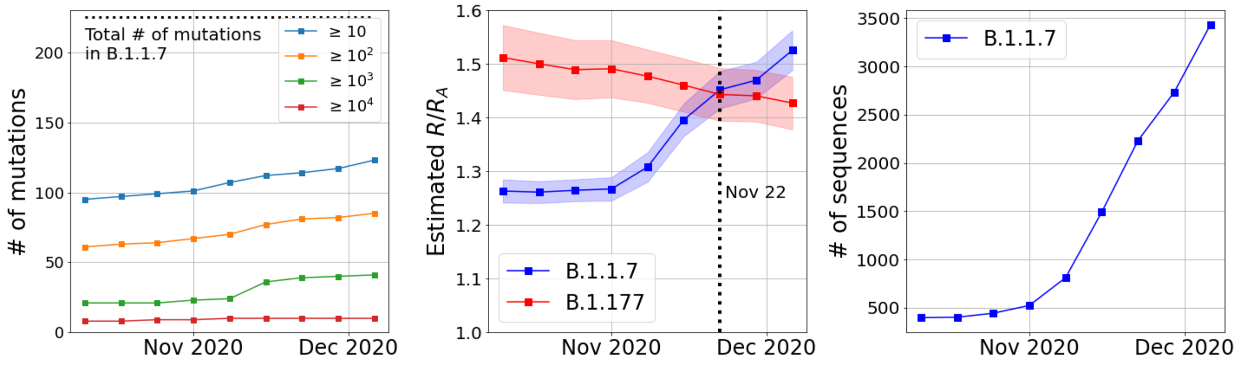

Figure S9. We depict the ability of PyR_0 to predict the fitness of Alpha lineage B.1.1.7 as the number of sequenced genomes increased throughout the last two months of 2020. PyR_0 predicted that B.1.1.7 was more fit than B.1.177—the dominant lineage in England before B.1.1.7 took over—by November 22nd 2020. Note the substantial number of mutations that appear in the various sublineages that constitute B.1.1.7. **Left:** The number of amino acid mutations that make up B.1.1.7 that had been observed in at least $10/10^2/10^3/10^4$ sequences by the given date. **Middle:** Estimates of R/R_A using sequences collected by the given date. **Right:** The total number of B.1.1.7 sequences collected by the given date.

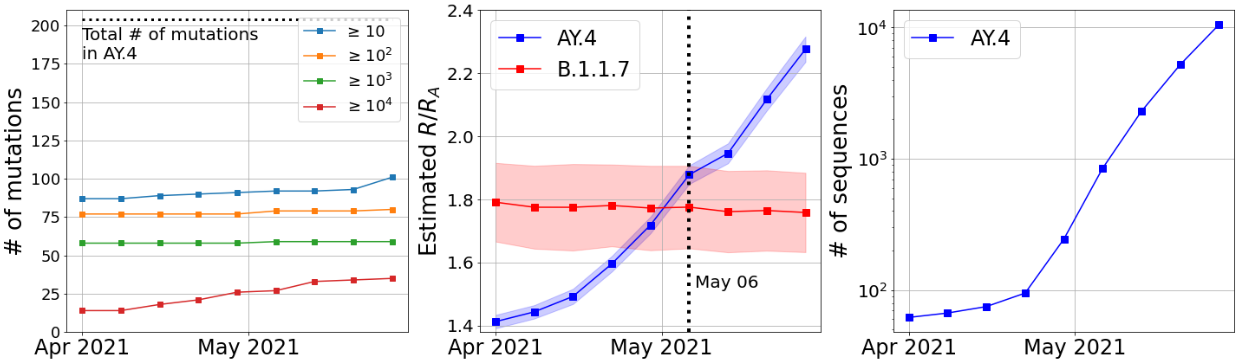

Figure S10. We depict the ability of PyR_0 to predict the fitness of Delta sublineage AY.4 as the number of sequenced genomes increased throughout Spring 2021, comparing to B.1.1.7, which was the dominant lineage in England before AY.4 took over in mid-May (see Figure S5). By April 29th PyR_0 predicted that AY.4 was more fit than the least fit B.1.1.7 sublineages, at which point 245 AY.4 sequences had been collected. By May 6th PyR_0 predicted that AY.4 was more fit than most B.1.1.7 sublineages, at which point 847 AY.4 sequences had been collected. Note that there is significant heterogeneity in the estimated fitness of B.1.1.7 sublineages. **Left:** The number of amino acid mutations that make up AY.4 that had been observed in at least $10/10^2/10^3/10^4$ sequences by the given date. **Middle:** Estimates of R/R_A using sequences collected by the given date. **Right:** The total number of AY.4 sequences collected by the given date.

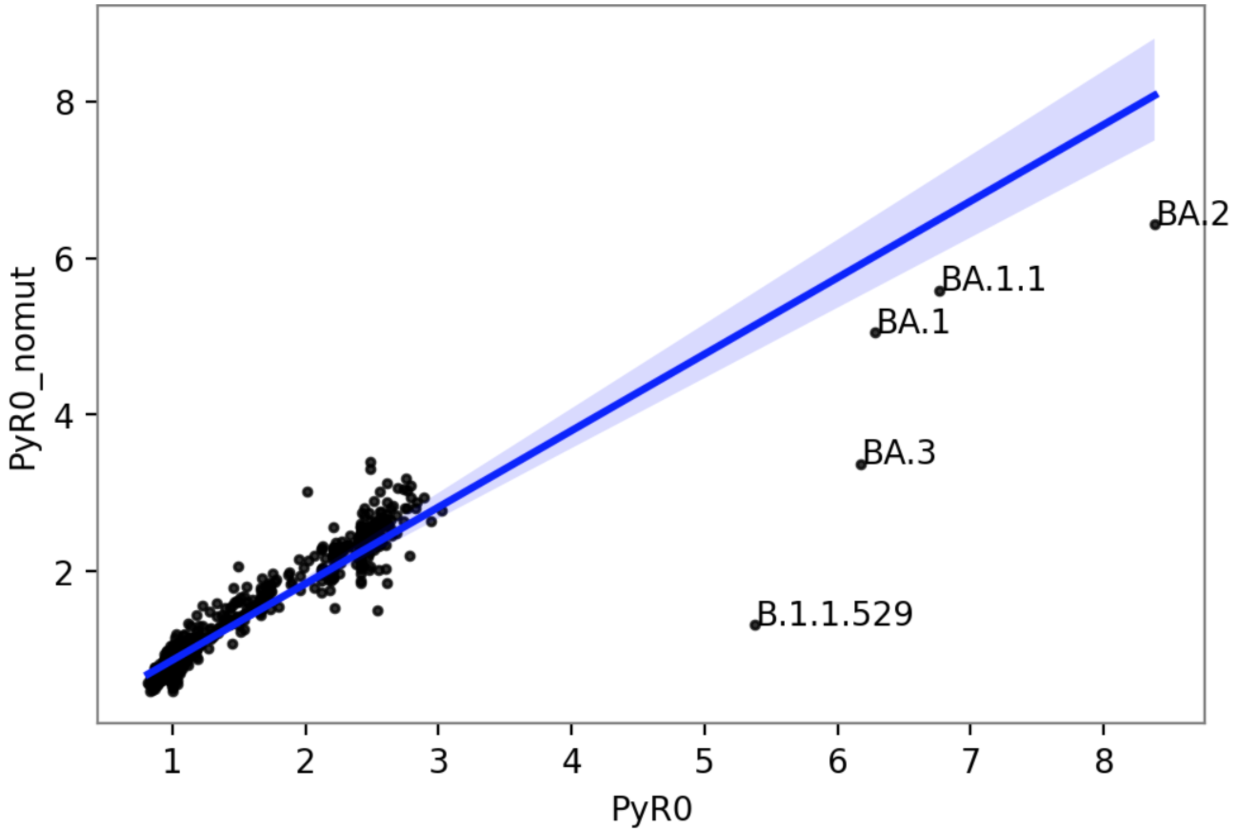

Figure S11: Comparison of strain-level fitness estimates for PyR_0 and PyR_0 with no mutations. A linear regression line with standard error is shown. Estimates between the two models were concordant (Pearson R = 0.95), but the mutation-aware model tended to assign higher fitness to Omicron lineages based on shared mutations, including lineages that were minimally sampled (B.1.1.529 and BA.3).

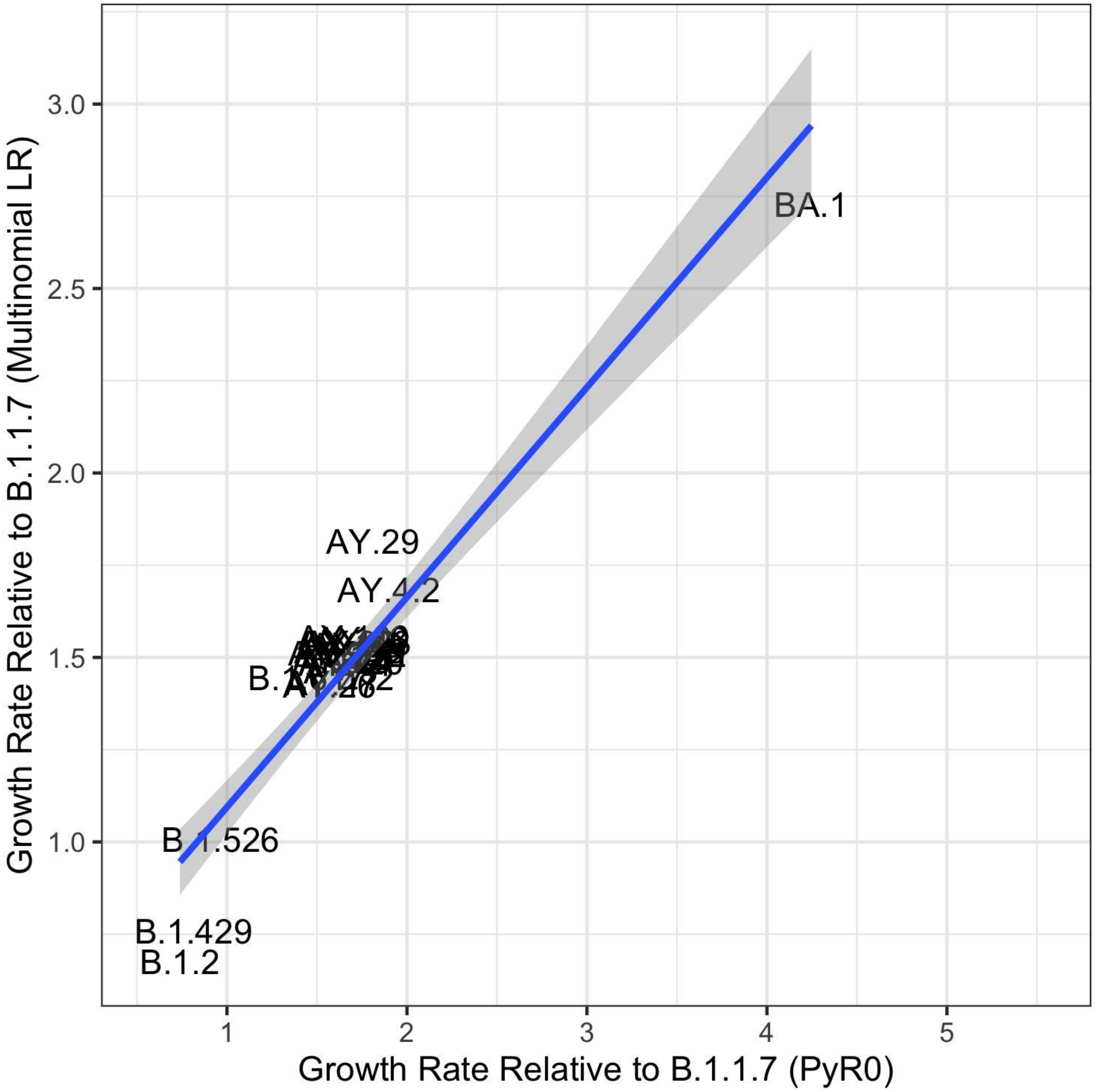

Figure S12. Comparison of mutation-level regression coefficients for growth rate among 50 most prevalent lineages using a standard multinomial logistic regression model with estimates of lineage growth rates from PyR_0. Pearson's R = 0.95.

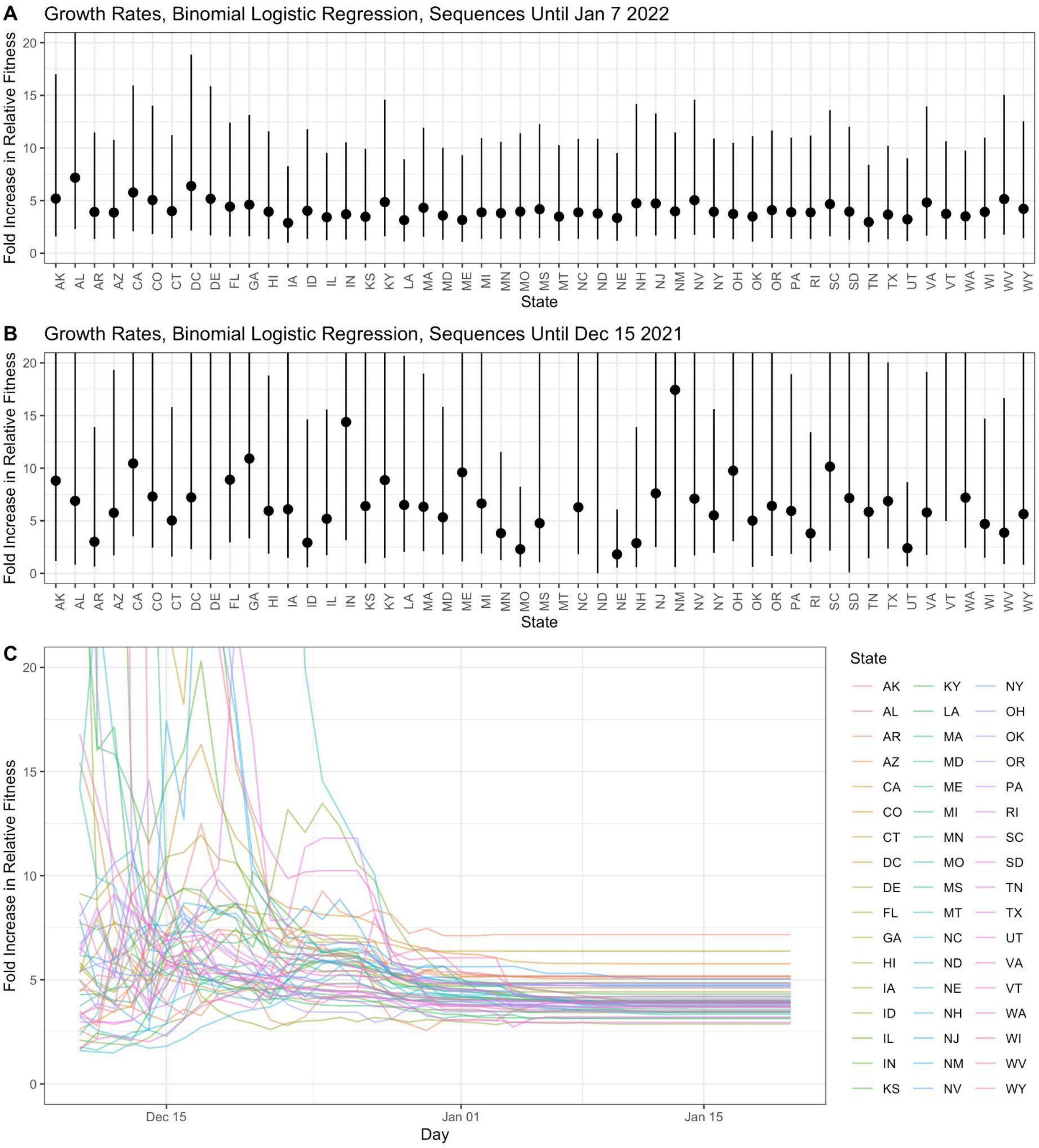
A
Growth Rates, Binomial Logistic Regression, Sequences Until Jan 7 2022
Fold Increase in Relative Fitness
State
B
Growth Rates, Binomial Logistic Regression, Sequences Until Dec 15 2021
Fold Increase in Relative Fitness
State
C
Fold Increase in Relative Fitness
Day
Dec 15
Jan 01
Jan 15
State
AK AL AR AZ CA CO CT DC DE FL GA HI IA ID IL IN KS KY LA MA MD ME MI MN MO MS MT NC ND NE NH NJ NM NV NY OH OK OR PA RI SC SD TN TX UT VA VT WA WI WV WY

D

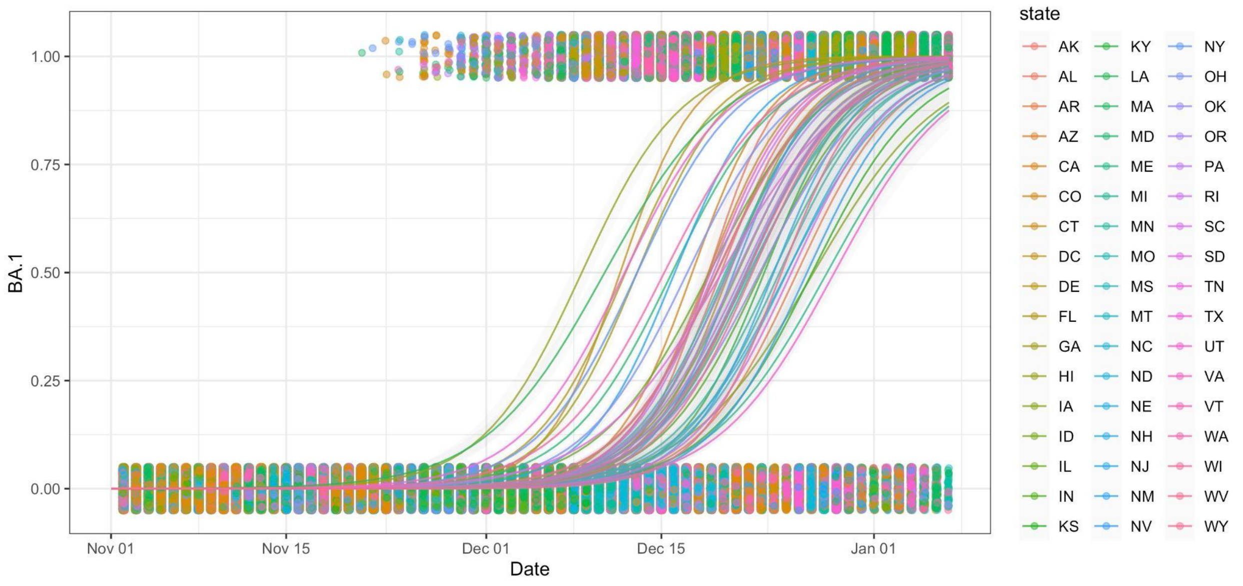

Figure S13. **A.** Estimated growth rate per viral generation (5.5 days) in each state using binomial logistic regressions for the emergence of BA.1 against a background consisting of Delta (B.1.617.2 and sublineages) viruses in all 50 US states between November 1 2021 and January 7 2022. Fold increase in relative fitness is expressed as exp($\boldsymbol{\beta}_1$), where time is measured in viral generations. Error bars show exp($\boldsymbol{\beta}_1$ +/- SE($\boldsymbol{\beta}_1$)). For all 50 US states, the median growth rate per viral generation of Omicron over Delta was 3.9. For all states, the confidence interval for the binomial logistic regression coefficient contained the estimate for the ratio of Omicron to Delta from the PyR$_0$ model, which was 3.1 for BA.1.1 / B.1.617.2 and 2.8 for BA.1 / B.1.617.2. **B.** Estimated growth rate per viral generation for genomes censored through December 15 2021. **C.** Estimated growth rates per viral generation for genomes censored daily from December 10 2021 through January 7 2022. **D.** Estimated probability of BA.1 by state from the binomial logistic regression using data through January 7 2022; coefficients are summarized in panel **A**.

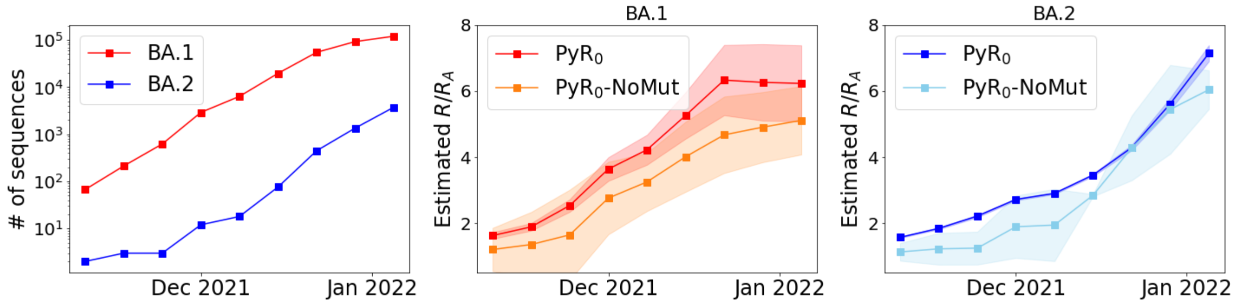

Figure S14. In this companion figure to Figure S8 we demonstrate that PyR_0's ability to estimate the fitness of newly emergent lineages degrades if mutational profiles are not part of the model (denoted PyR_0-NoMut). **Left:** The total number of BA.1/BA.2 sequences collected by the given date. **Middle/Right:** Estimates of R/R_A using sequences collected by the given date.

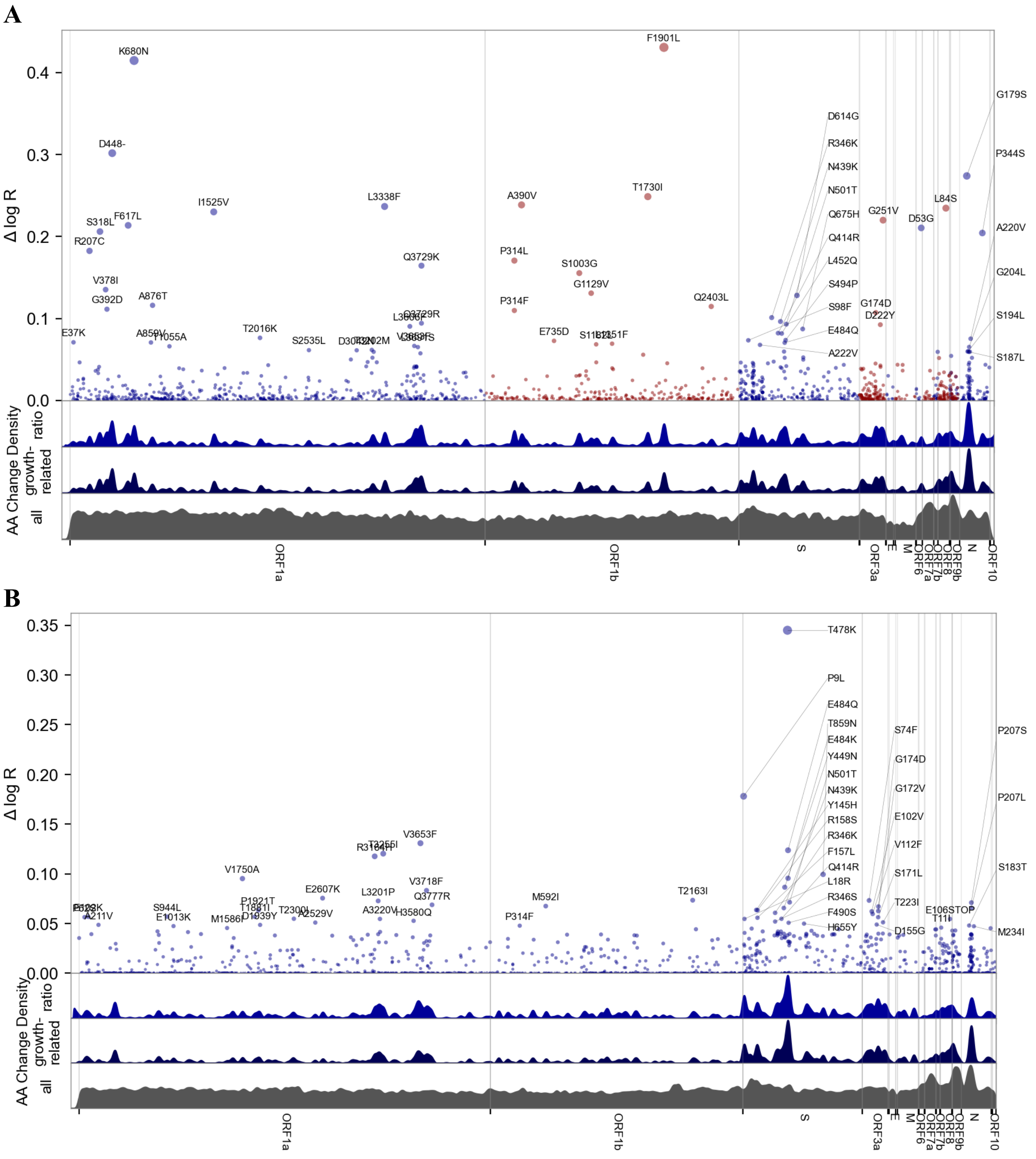
A
Δ log R
AA Change Density
ratio
growth-related
all
K680N
F1901L
D448-
G179S
P344S
D614G
R346K
N439K
N501T
Q675H
Q414R
L452Q
S494P
S98F
E484Q
A222V
I1525V
L3338F
A390V
T1730I
G251V
L84S
D53G
A220V
S318L
F617L
R207C
Q3729K
P314L
S1003G
G1129V
V378I
G392D
A876T
P314F
Q2403L
G174D
D222Y
G204L
S194L
S187L
E37K
A859V
T1055A
T2016K
S2535L
Q3729R
E735D
ORF1a
ORF1b
S
ORF3a
E
M
ORF6
ORF7a
ORF7b
ORF8
ORF9b
N
ORF10
B
T478K
P9L
E484Q
T859N
E484K
Y449N
N501T
N439K
Y145H
R158S
R346K
F157L
Q414R
L18R
R346S
F490S
H655Y
S74F
G174D
G172V
E102V
V112F
S171L
T223I
D155G
E106STOP
T11I
P207S
P207L
S183T
M234I
V3653F
V1750A
E2607K
V3718F
L3201P
Q3777R
M592I
T2163I
P314F
S944L
E1013K
A211V
P1921T
T2300I
A2529V
A3220V
H3580Q
M1586I
D1939Y
0.35
0.30
0.25
0.20
0.15
0.10
0.05
0.00
0.4
0.3
0.2
0.1
0.0

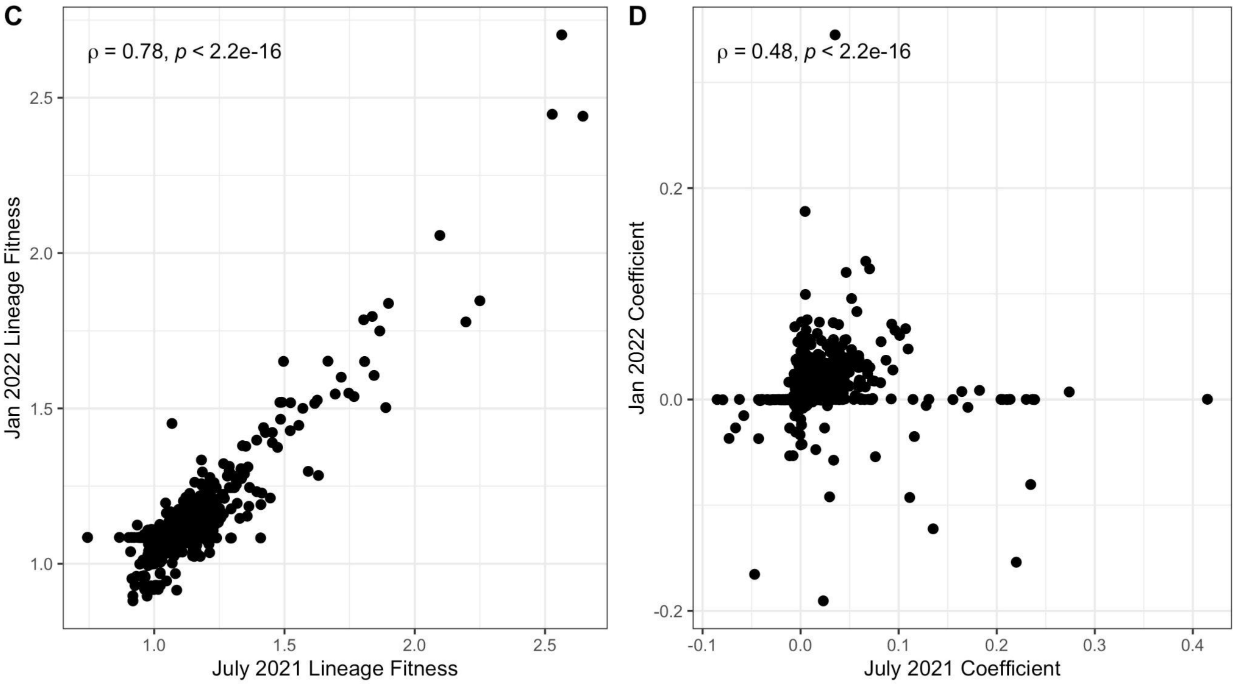
C
ρ = 0.78, p < 2.2e-16
Jan 2022 Lineage Fitness
July 2021 Lineage Fitness
1.0
1.5
2.0
2.5
D
ρ = 0.48, p < 2.2e-16
Jan 2022 Coefficient
July 2021 Coefficient
-0.2
0.0
0.2
-0.1
0.1
0.3
0.4

E

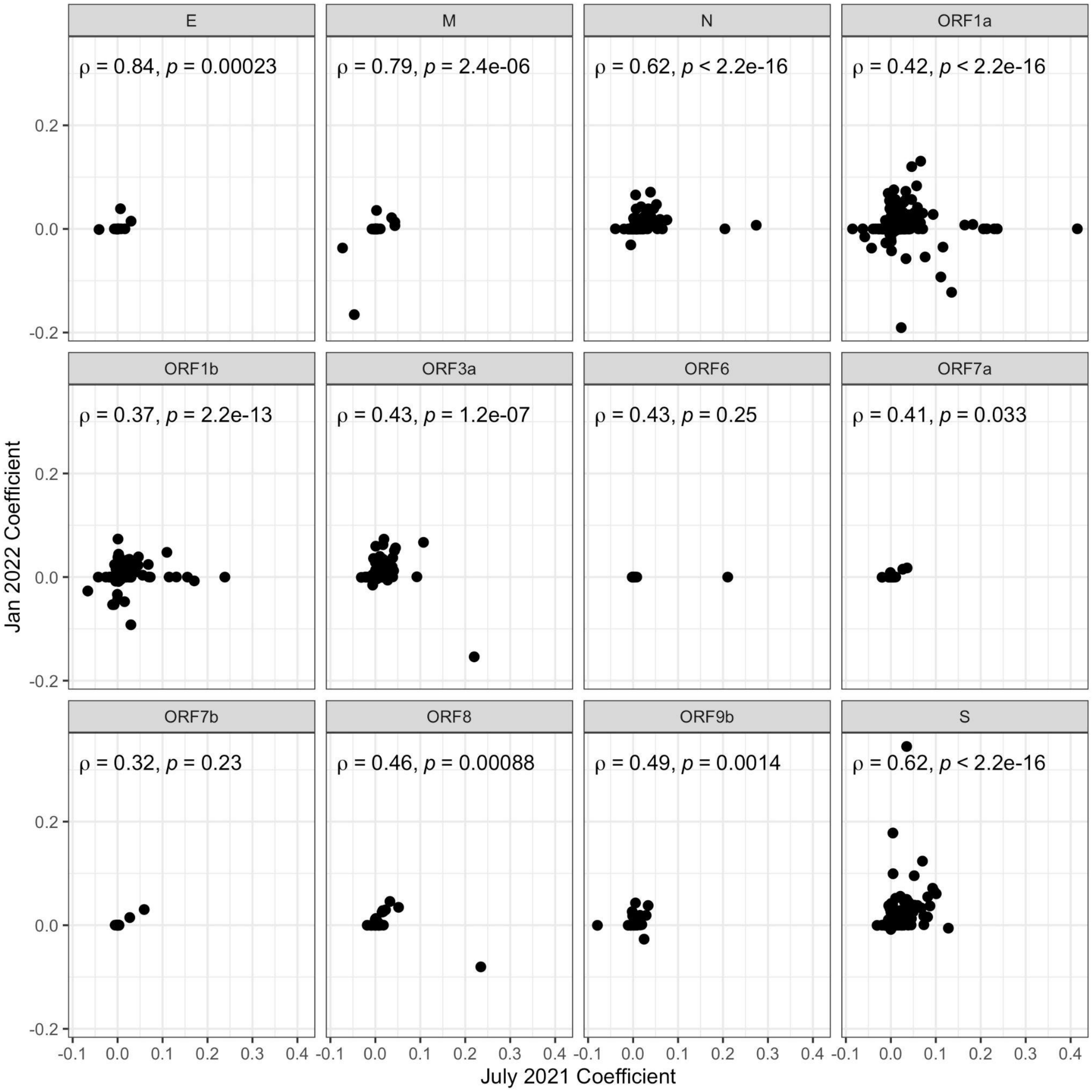

E
M
N
ORF1a
ρ = 0.84, p = 0.00023
ρ = 0.79, p = 2.4e-06
ρ = 0.62, p < 2.2e-16
ρ = 0.42, p < 2.2e-16
ORF1b
ORF3a
ORF6
ORF7a
ρ = 0.37, p = 2.2e-13
ρ = 0.43, p = 1.2e-07
ρ = 0.43, p = 0.25
ρ = 0.41, p = 0.033
ORF7b
ORF8
ORF9b
S
ρ = 0.32, p = 0.23
ρ = 0.46, p = 0.00088
ρ = 0.49, p = 0.0014
ρ = 0.62, p < 2.2e-16
Jan 2022 Coefficient
July 2021 Coefficient
0.2
0.0
-0.2
-0.1
0.0
0.1
0.2
0.3
0.4

F

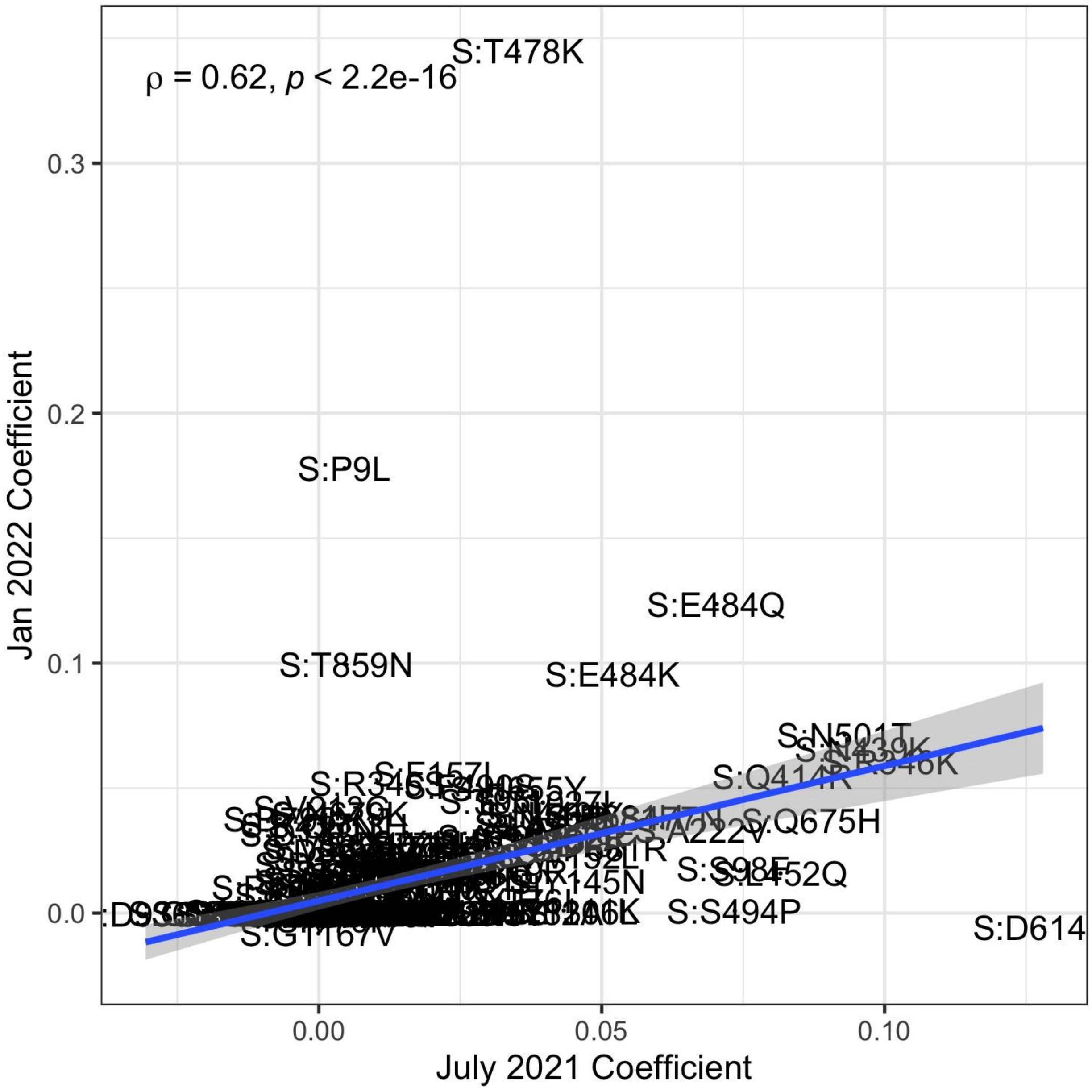

Figure S15. Comparison of estimates from 6.4 million genomes analyzed here to pre-Omicron model using 2.1 millions from July 6 2021*(17)* for strains and mutations included in both models. **A.** Manhattan plot from a model built using 2.1 million genomes downloaded July 6 2021. **B.** Manhattan plot from a model built using 6.4 million genomes downloaded January 20 2022. **C.** Comparison of fitness estimates (R/R_A) by lineage. **D.** Comparison of fitness estimates for mutations throughout the genome. **E.** Comparison of fitness estimates for mutations in each genomic region. **F.** Comparison of fitness estimates for mutations for Spike. A regression line with standard error is shown. In **C-F**, ρ denotes Spearman correlation coefficients. P values report a test of no association.

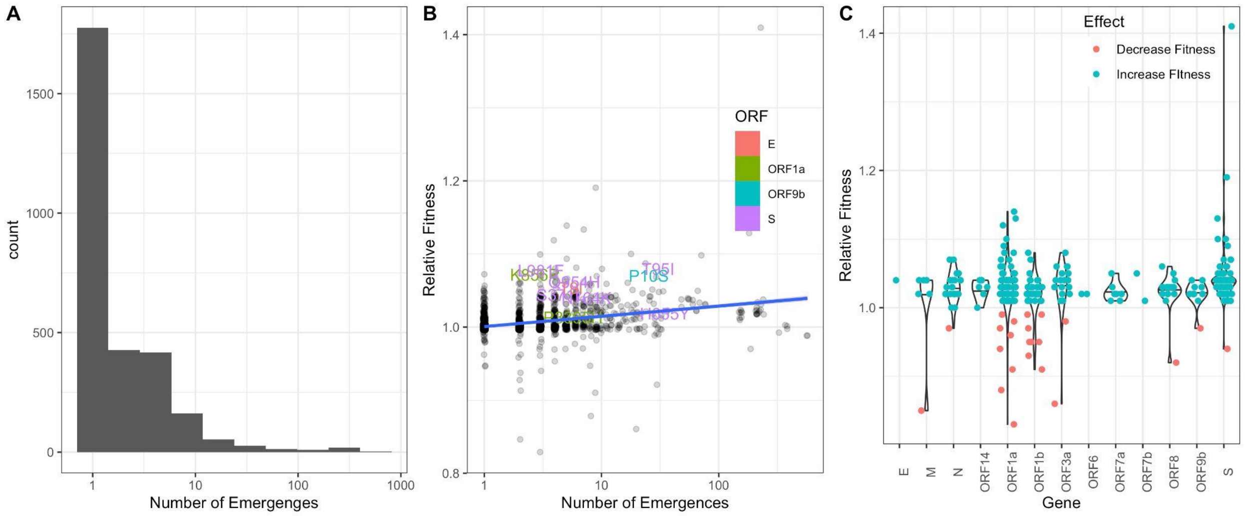

Figure S16. A. Histogram of the number of independent emergences across all observed mutations. A mutation was considered to have emerged independently if it was present in a lineage but not in its parent. **B.** Scatterplot of the fold-change in fitness versus the number of independent emergences. The top 10 ranked mutations are labeled, colored by ORF. Linear regression with standard error for the slope given as shaded area. **C.** Violin plots of fold-change in fitness for mutation, grouped by gene. The top 10% most statistically significant mutations are shown (where significance is determined by z-score of the approximate variational posterior).

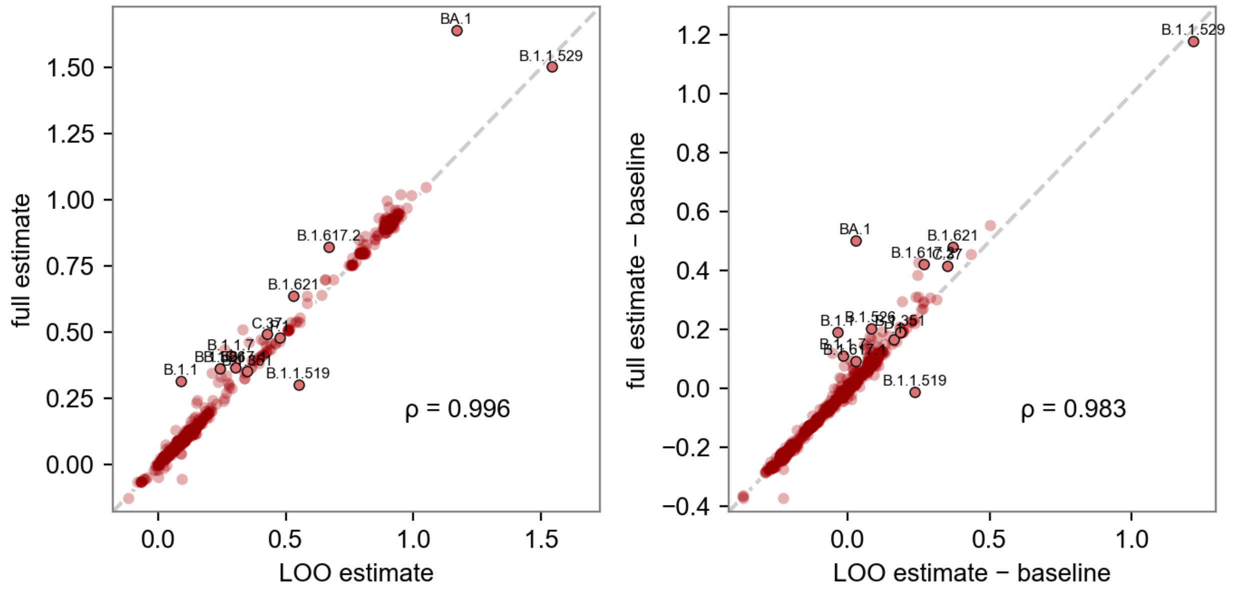

Figure S17. Accuracy of predicted fitness based solely on mutation content. **Left:** estimated log(R/R_A) for each lineage based on the full set of GISAID samples (y axis), and on the leave-one-out subset with each lineage's subclade removed and the fitness estimated from the mutations present in the lineage (x axis). **Right:** the same quantities but relative to a baseline estimator in which each child lineage's fitness is the same as that of its parent lineage. If a mutation is entirely removed from the LOO dataset, then the corresponding mutation coefficient is estimated as zero. The evaluation metrics are Pearson correlation (ρ) and mean absolute error (MAE). The MAE of the leave-one-out estimator is 0.001, more than 100x smaller than the MAE of the baseline estimator (0.129). Both panels highlight the CDC's variants of concern and variants of interest. The lineages selected for testing are those with at least 100 samples and with the largest deviations from their parent, i.e. where the baseline estimator performs worst. Note that the fitness of child lineages can deviate substantially from that of the parent, e.g. BA.1 is surprisingly higher fitness than its parent B.1.1.529.

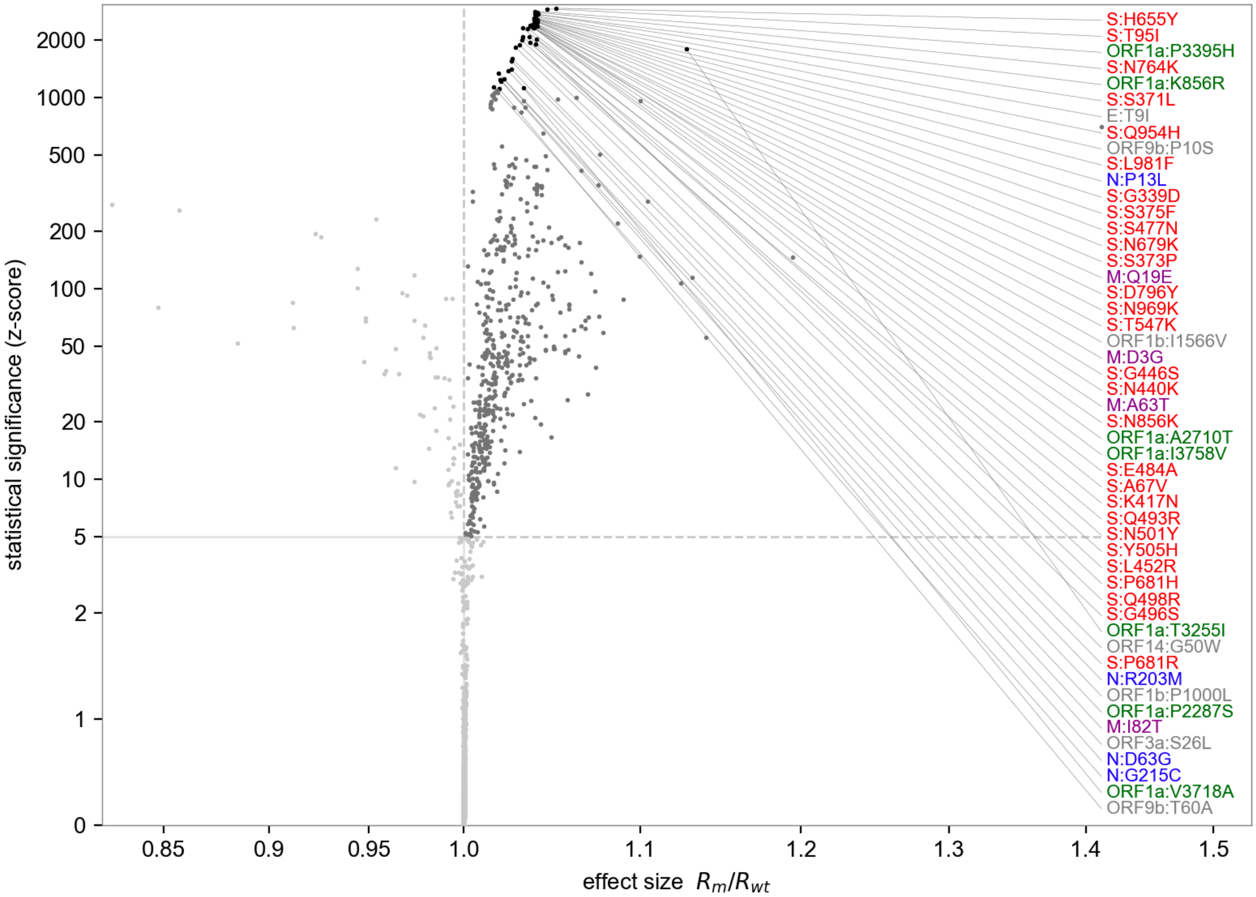

Figure S18. Volcano plot highlighting the most statistically significant mutations linked to increased fitness. The x-axis depicts the effect size as a ratio of estimated fitness of lineages with-versus-without each mutation. The y-axis depicts z-score from the approximate variational posterior as a proxy for statistical significance. The top 50 most statistically significant mutations are labeled, colored by gene. The 540 growth-associated mutations with z-score greater than 5 are shaded dark gray.

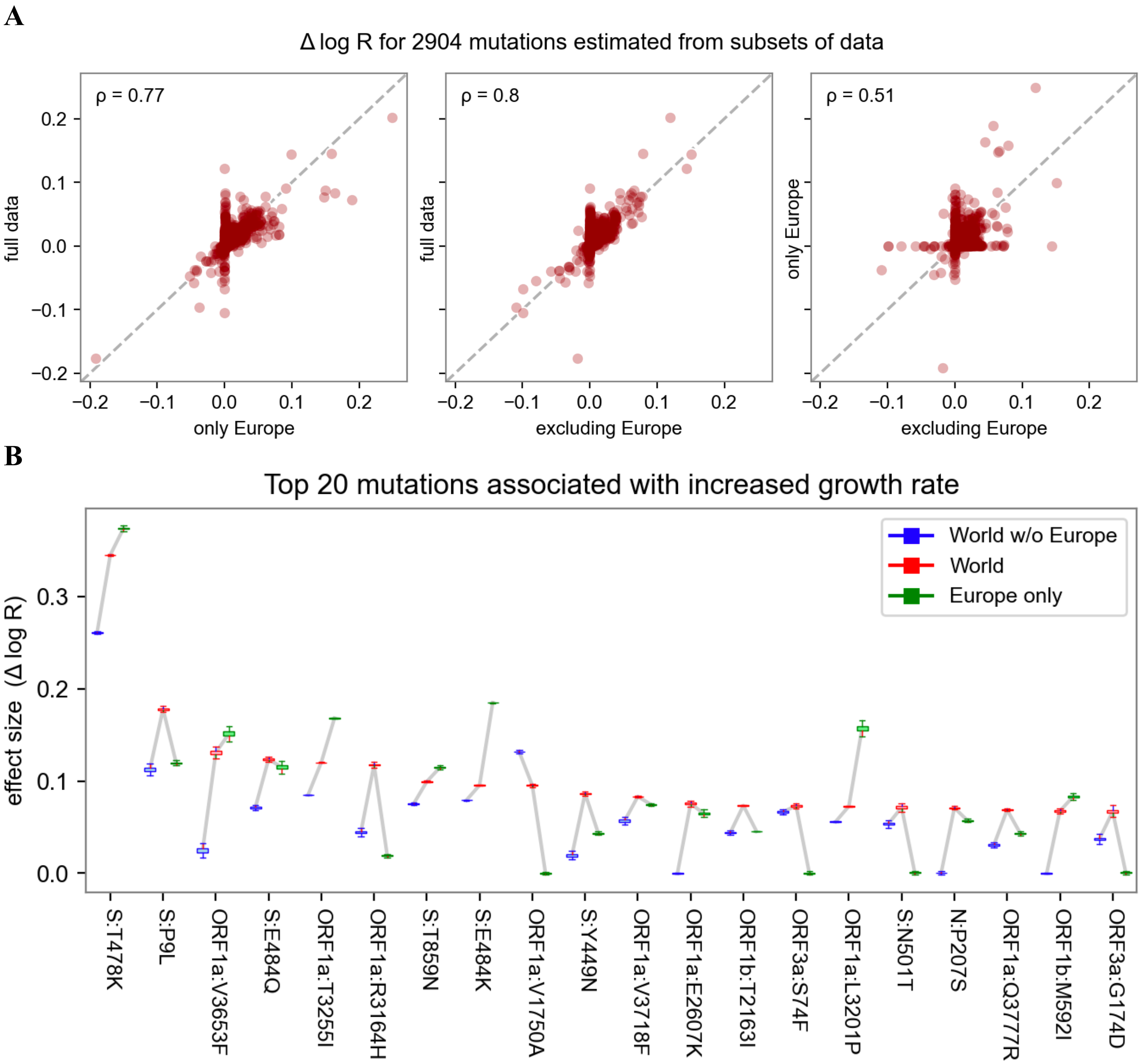
A
Δ log R for 2904 mutations estimated from subsets of data
ρ = 0.77
ρ = 0.8
ρ = 0.51
full data
full data
only Europe
only Europe
excluding Europe
excluding Europe
0.2
0.1
0.0
−0.1
−0.2
B
Top 20 mutations associated with increased growth rate
World w/o Europe
World
Europe only
effect size (Δ log R)
0.3
0.2
0.1
0.0
S:T478K
S:P9L
ORF1a:V3653F
S:E484Q
ORF1a:T3255I
ORF1a:R3164H
S:T859N
S:E484K
ORF1a:V1750A
S:Y449N
ORF1a:V3718F
ORF1a:E2607K
ORF1b:T2163I
ORF3a:S74F
ORF1a:L3201P
S:N501T
N:P207S
ORF1a:Q3777R
ORF1b:M592I
ORF3a:G174D

C

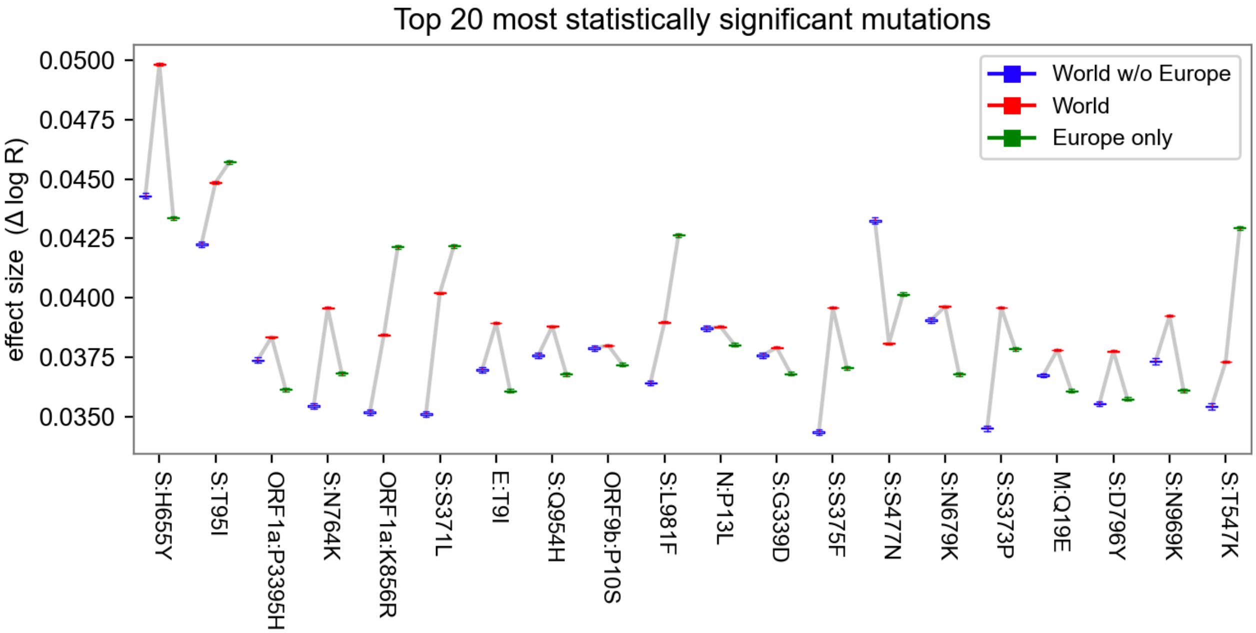

Figure S19. Sensitivity of mutation estimates. **A.** Scatterplot of the mutation coefficients on the full model and geographic subsets, with Pearson correlation (ρ) as shown. **B.** Box-and-whisker plot depicting estimated growth rates with corresponding uncertainties for the 20 lineages with highest growth rate (effect size) across geographic subsets. **C.** Same as B but with the top 20 lineages sorted by statistical significance (z-score). Note that in B, the World estimates (center) tend to be higher than subsets (left and right) only because the ranked selection is based on those estimates.

A

B

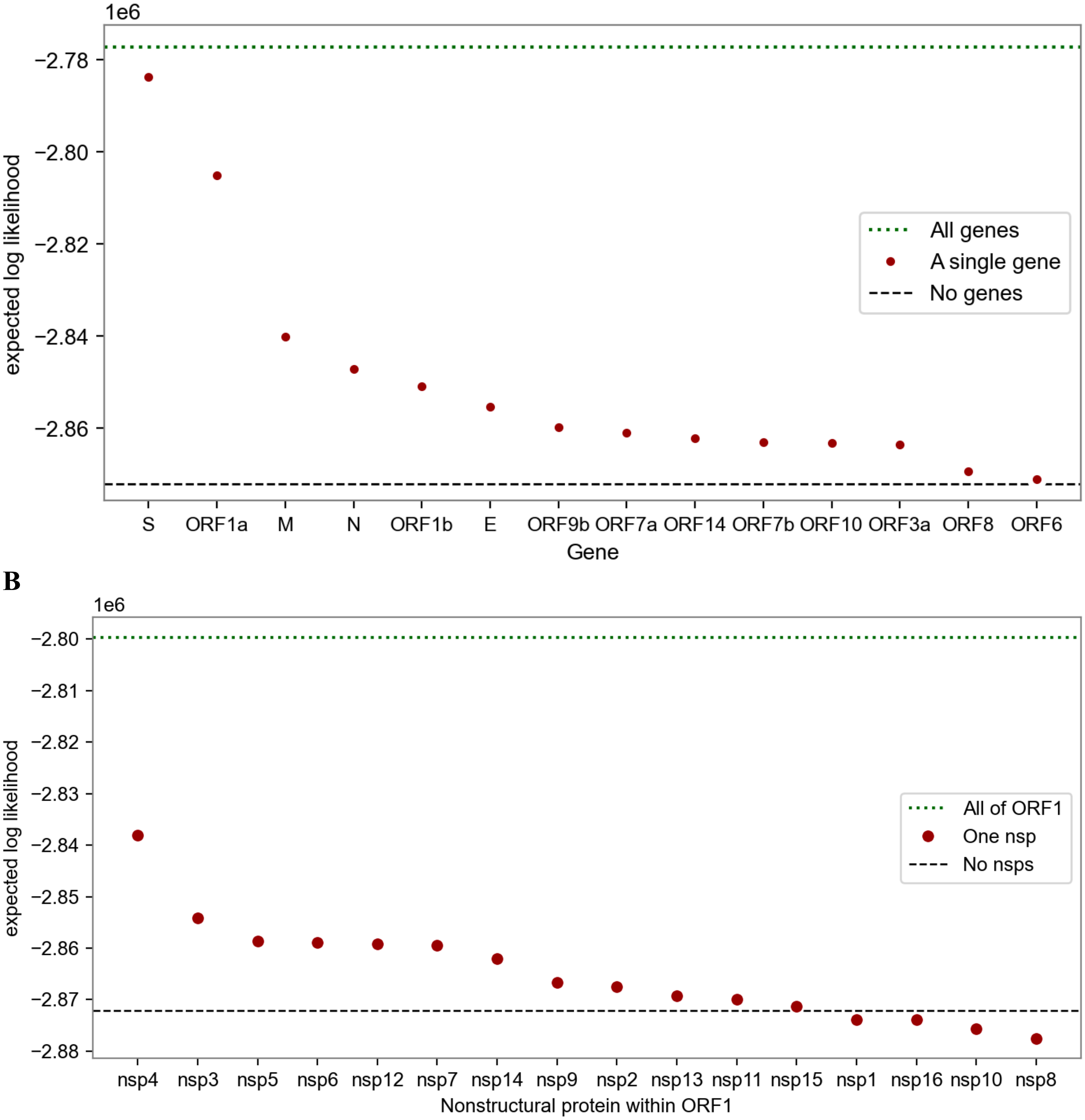

Figure S20. Information content of different subsets of the SARS-CoV-2 genome in explaining fitness. The metric is the expected log likelihood. The dotted line at the top shows the performance of the full model that regresses against all genes (A) or against all of ORF1 (B). The circles show estimators based on only single genes (A) or single nonstructural proteins (B). The most informative genes are S, ORF1a, M, N, and ORF1b; within ORF1 the most informative nsps are nsp4, 3, 5, 6, 12 and 7. The bottom dashed lines show the performance of a naive estimator that ignores genetic information, effectively estimating each lineage's growth rate in each region independent of growth rate estimates in other regions.

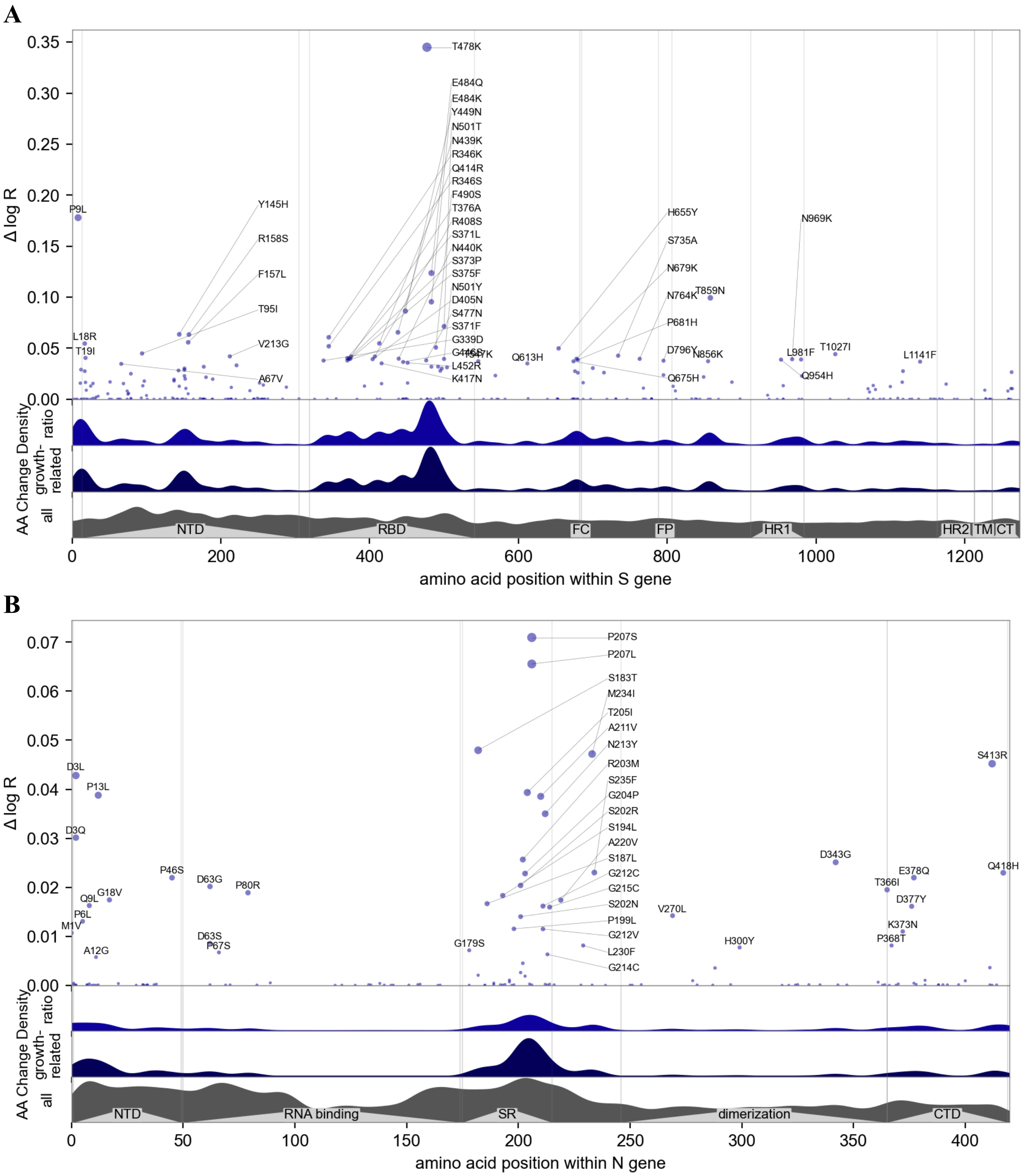
A
Δ log R
0.35
0.30
0.25
0.20
0.15
0.10
0.05
0.00
T478K
E484Q
E484K
Y449N
N501T
N439K
R346K
Q414R
R346S
F490S
T376A
R408S
S371L
N440K
S373P
S375F
N501Y
D405N
S477N
S371F
G339D
G446S
T547K
L452R
K417N
P9L
Y145H
R158S
F157L
T95I
V213G
A67V
L18R
T19I
Q613H
H655Y
S735A
N679K
N764K
T859N
P681H
D796Y
N856K
Q675H
N969K
L981F
T1027I
Q954H
L1141F
AA Change Density
ratio
growth-related
all
NTD
RBD
FC
FP
HR1
HR2
TM
CT
0
200
400
600
800
1000
1200
amino acid position within S gene
B
Δ log R
0.07
0.06
0.05
0.04
0.03
0.02
0.01
0.00
P207S
P207L
S183T
M234I
T205I
A211V
N213Y
R203M
S235F
G204P
S202R
S194L
A220V
S187L
G212C
G215C
S202N
P199L
G212V
L230F
G214C
D3L
P13L
D3Q
P46S
D63G
P80R
Q9L
G18V
P6L
M1V
A12G
D63S
P67S
G179S
V270L
H300Y
D343G
S413R
Q418H
E378Q
T366I
D377Y
K373N
P368T
AA Change Density
ratio
growth-related
all
NTD
RNA binding
SR
dimerization
CTD
0
50
100
150
200
250
300
350
400
amino acid position within N gene

C

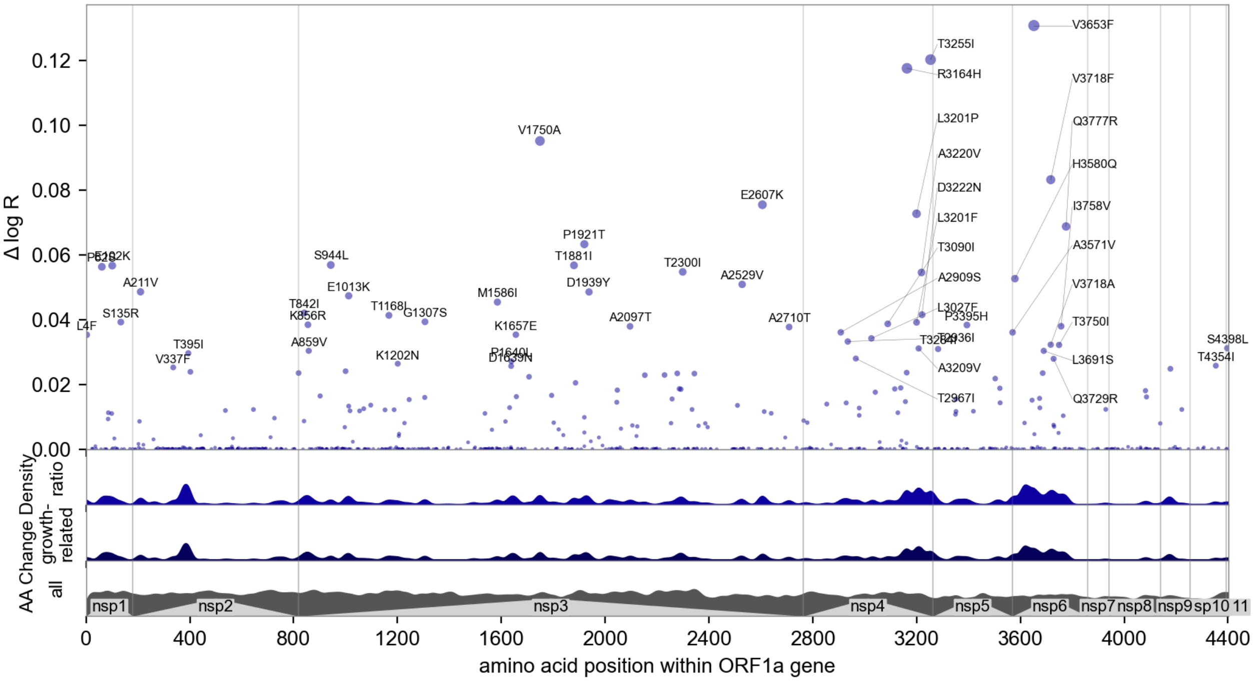

D

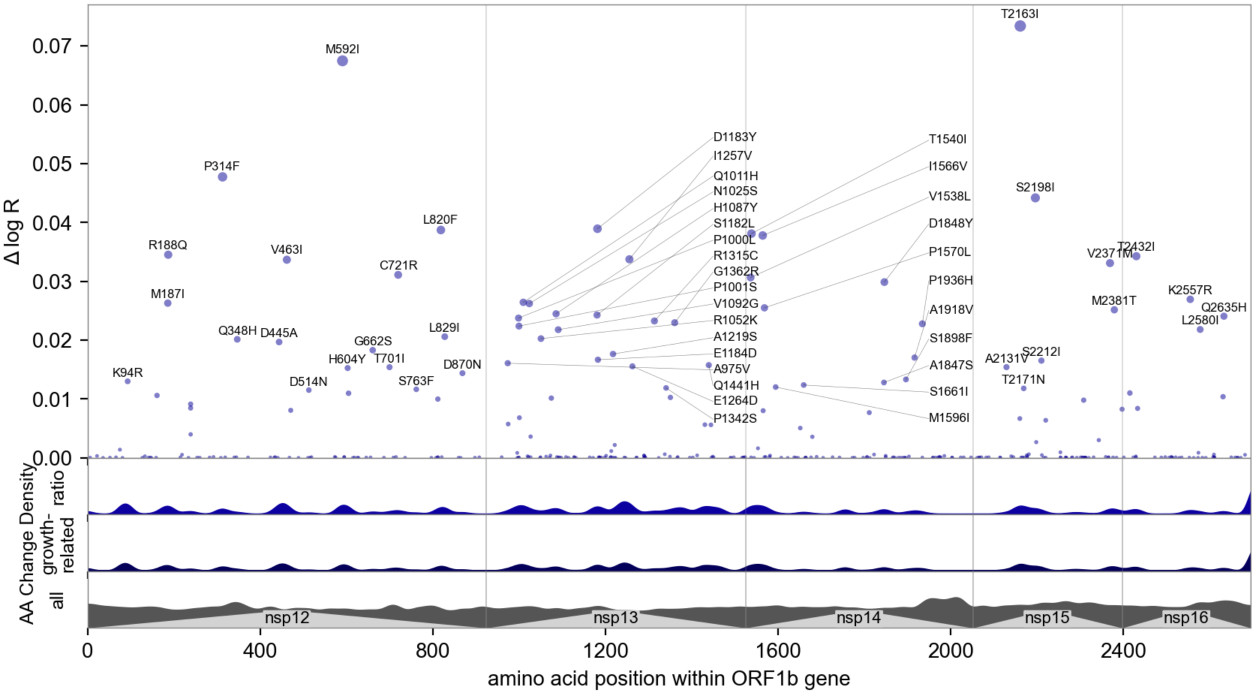

Figure S21. Manhattan plot details of the four most informative genes. See Figure 3 for a whole-genome view and Figure S12 for ranking by information. **A.** View of the 1237 amino acids of the S protein, annotated by structure *(53)*; many mutations occur in the N-terminal domain (NTD), receptor-binding domain (RBD), and furin cleavage (FC) site. Regions containing the fusion peptide (FP), heptad repeat (HR) 1 and 2, transmembrane domain (TM), and C-terminal domain (CTD) are annotated. **B.** View of the 419 amino acids of the nucleocapsid (N) protein domains, annotated by structure *(54)*; many mutations occur in the serine–arginine rich region (SR), identified by *(55)* as immunogenic. **C.** View of the ORF1a polyprotein, including 11 non-structural proteins (nsps). **D.** View of the ORF1b polyprotein, including nsp12-16; note the

amino acid positions do not account for 9 additional residues at the N-terminus of nsp12 (RNA polymerase) resulting from the -1 ribosomal frameshift.

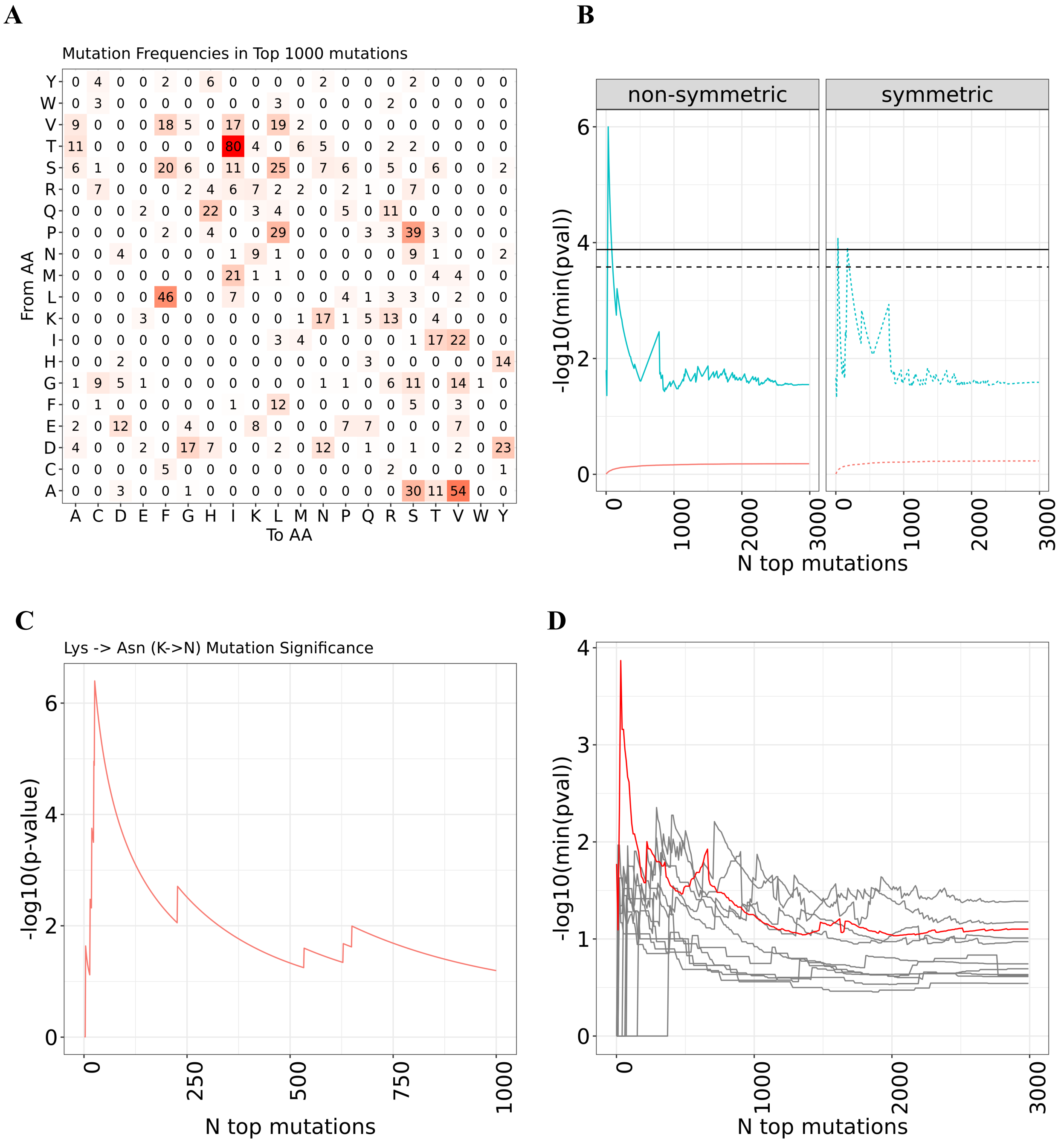

Figure S22: Enrichment analysis of amino acid changes among top-ranked mutations **A.** Mutation frequency in top 1000 most significant mutations (as ranked by posterior mean/stddev) **B.** Leading set enrichment analysis of most significant mutations predicted by the model for non-symmetrical (e.g. A->V) (left) and symmetrical (e.g. A<->V) (right) amino acid changes. The blue curve depicts the most significant p-value obtained for different top-N mutation cutoff values across all amino acid changes, while the red curve depicts the mean p-value. **C.** Further examination reveals that top mutations are enriched in K to N changes in the S gene. **D.** No other genes (gray) other than S (in red) show significant enrichment.

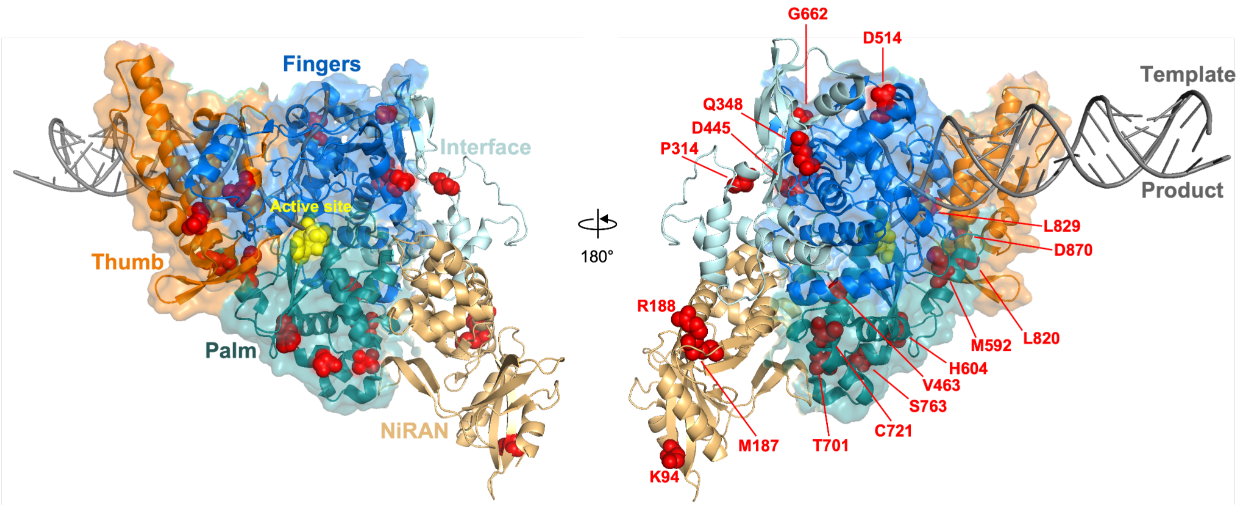

Figure S23. Top-ranked mutations in the viral RNA-dependent RNA polymerase (RdRP, nsp12, PDB: 7CYQ). Amino acid positions corresponding to top mutations are shown as red spheres. The catalytic site residues 750-SDD-752 are highlighted as yellow spheres. The coronavirus-specific domains (NiRAN, Interface) are shown as cartoon structures. The conserved RdRP domains (Fingers, Palm, Thumb) are shown as cartoon and surface filling structures.

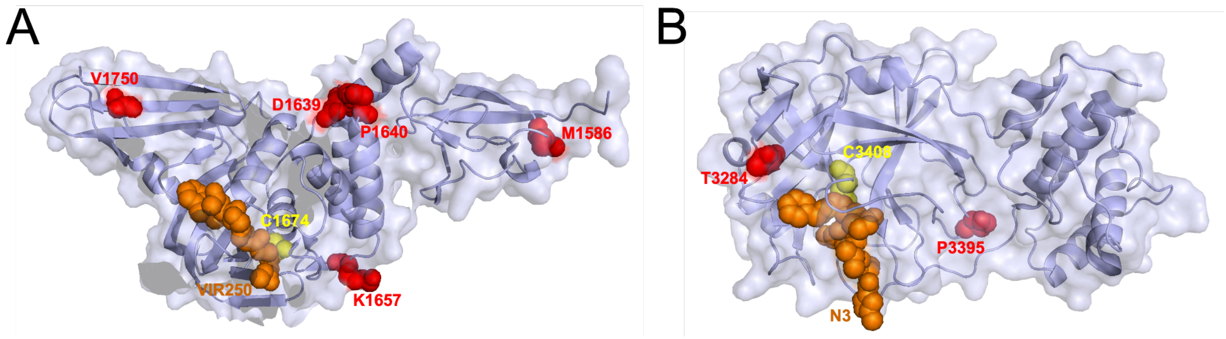

PLpro (nsp3, PDB: 6WUU)

M^{pro} (nsp5, PDB: 6LU7)

Figure S24. Top-ranked mutations in the two viral proteases, PLpro (A) and M^{pro} (B). Both protease structures are shown in light blue. Amino acid positions corresponding to top mutations are shown as red spheres. The catalytic cysteine residues for each are shown as yellow spheres. Two active-site inhibitors, VIR250 and N3, are shown as orange spheres.

A

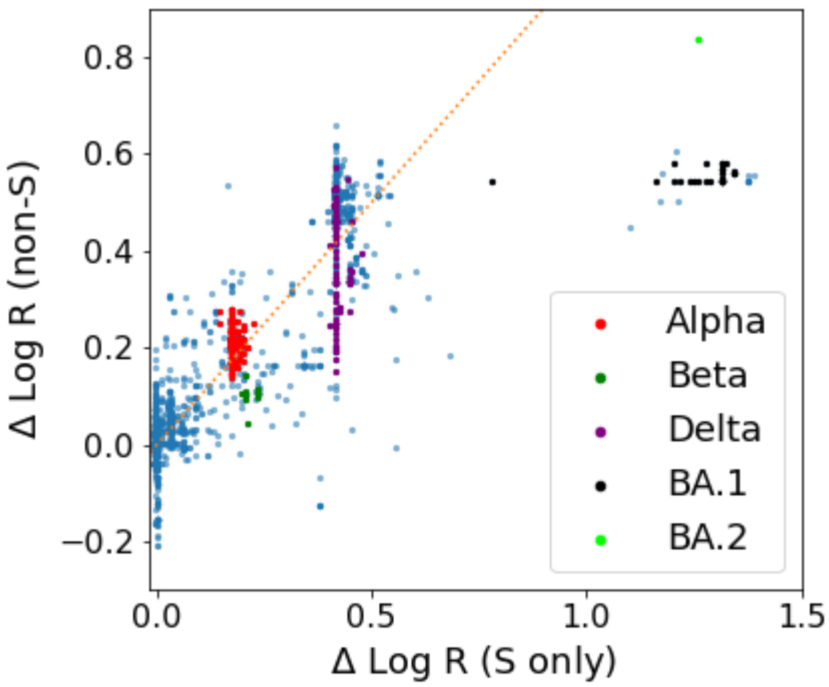

B

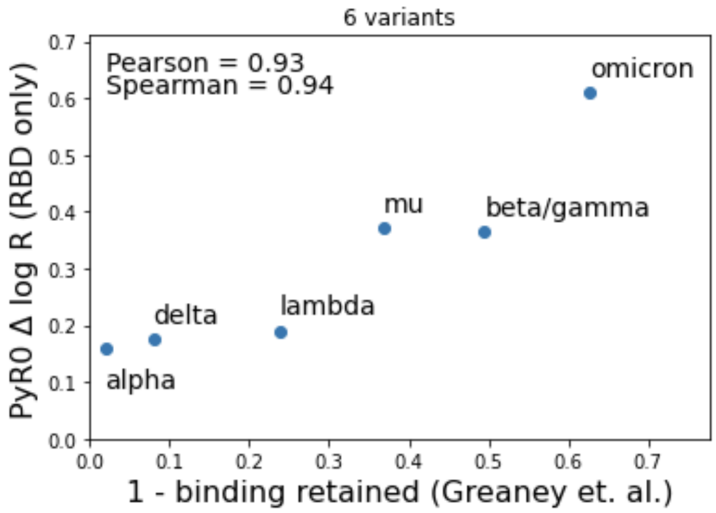

Figure S25. A. Estimated fitness contributions due to Spike and non-Spike mutations for the 3000 SARS-CoV-2 clusters in our analysis. The clusters corresponding to a few select PANGO lineages are colored. PyR_0 finds that the significant fitness heterogeneity exhibited by Alpha and Delta sublineages is largely driven by non-Spike mutations. **B.** Companion figure to Figure 3B. Mean fitness in RBD, aggregated across WHO variant designations, versus predicted antibody binding.

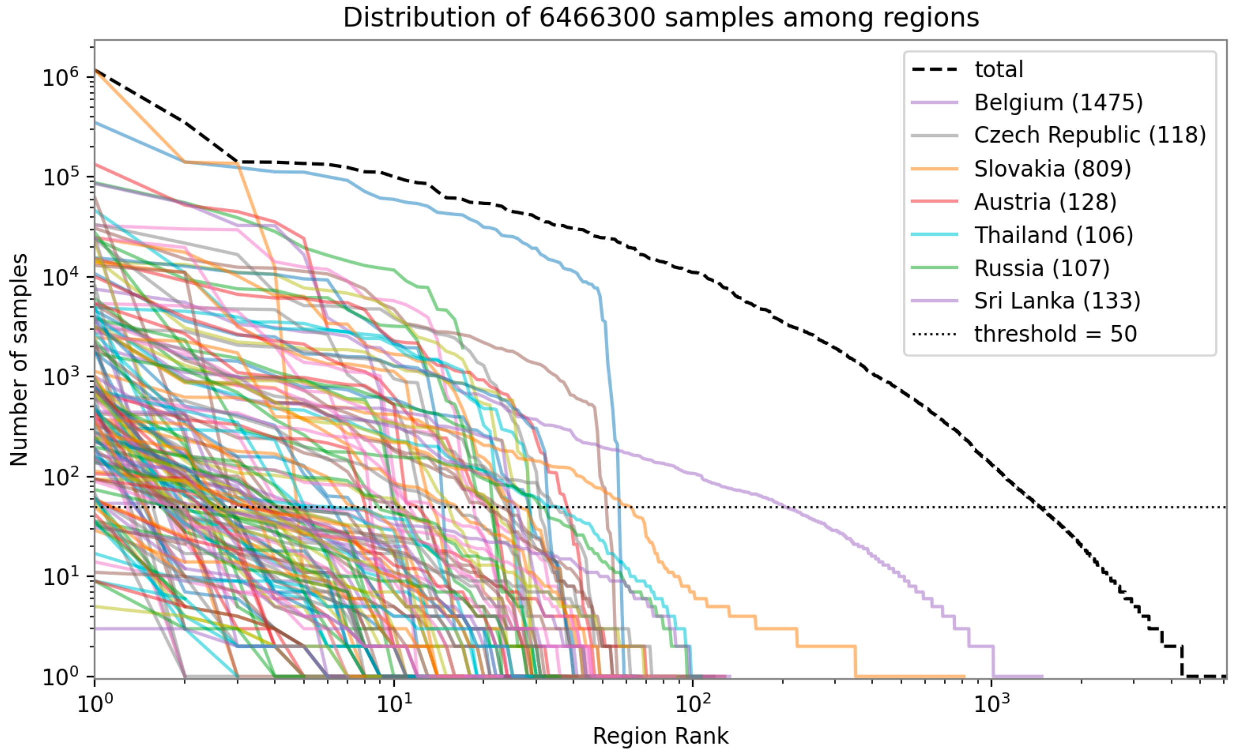

Figure S26. Distribution of samples among regions. Each 2nd level GISAID region (country) is plotted as a curve, with the sizes of 3rd level GISAID regions (usually provinces or states) plotted as points along the curve. The 3rd level is dominated by a few countries with many small regions (e.g. Belgium with 1475 regions), so we merge regions smaller than a threshold (50 samples) into their respective countries.

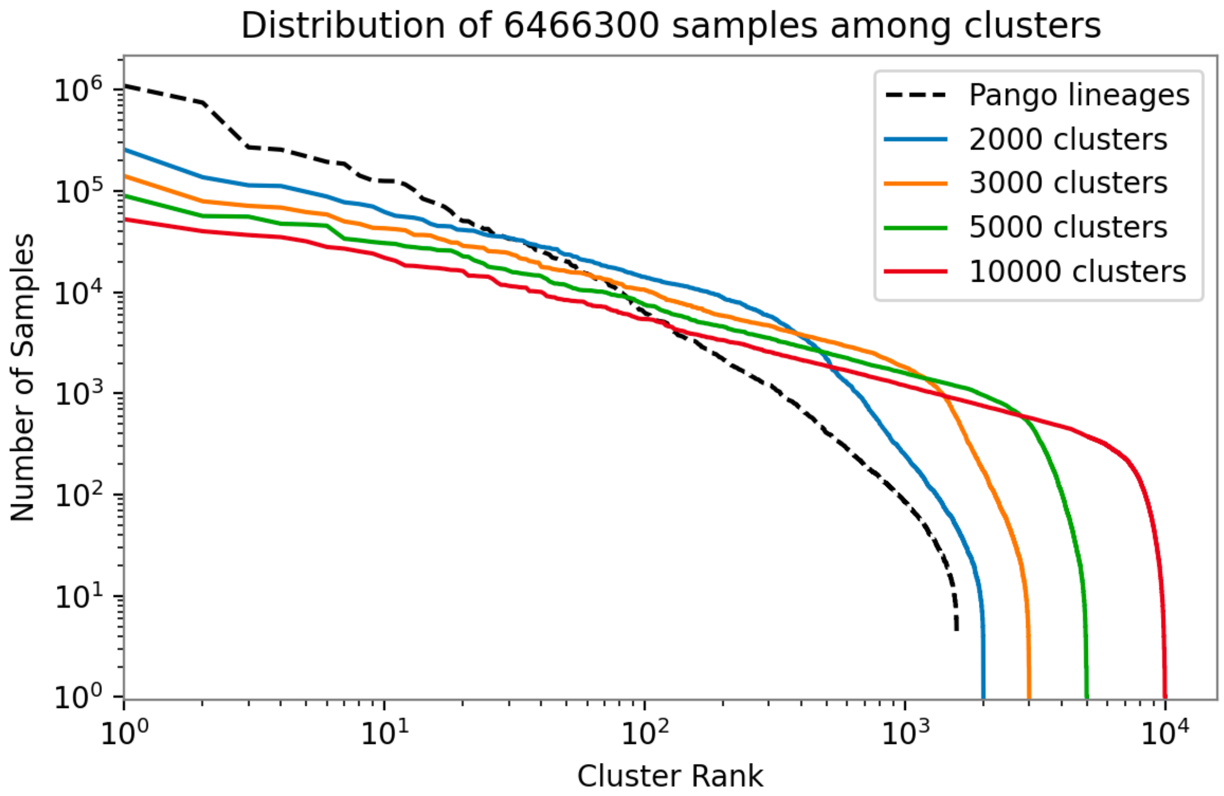

Figure S27. Distribution of samples among PANGO lineages and refined clusters. PANGO lineage sizes are heavy-tailed and appear heterogeneous, so we split into a larger number of clusters (colored). We chose a final clustering of 3000 clusters (orange), balancing between a smaller number of clusters (which improves statistical efficiency) and a larger number of clusters (which better represents lineage heterogeneity).

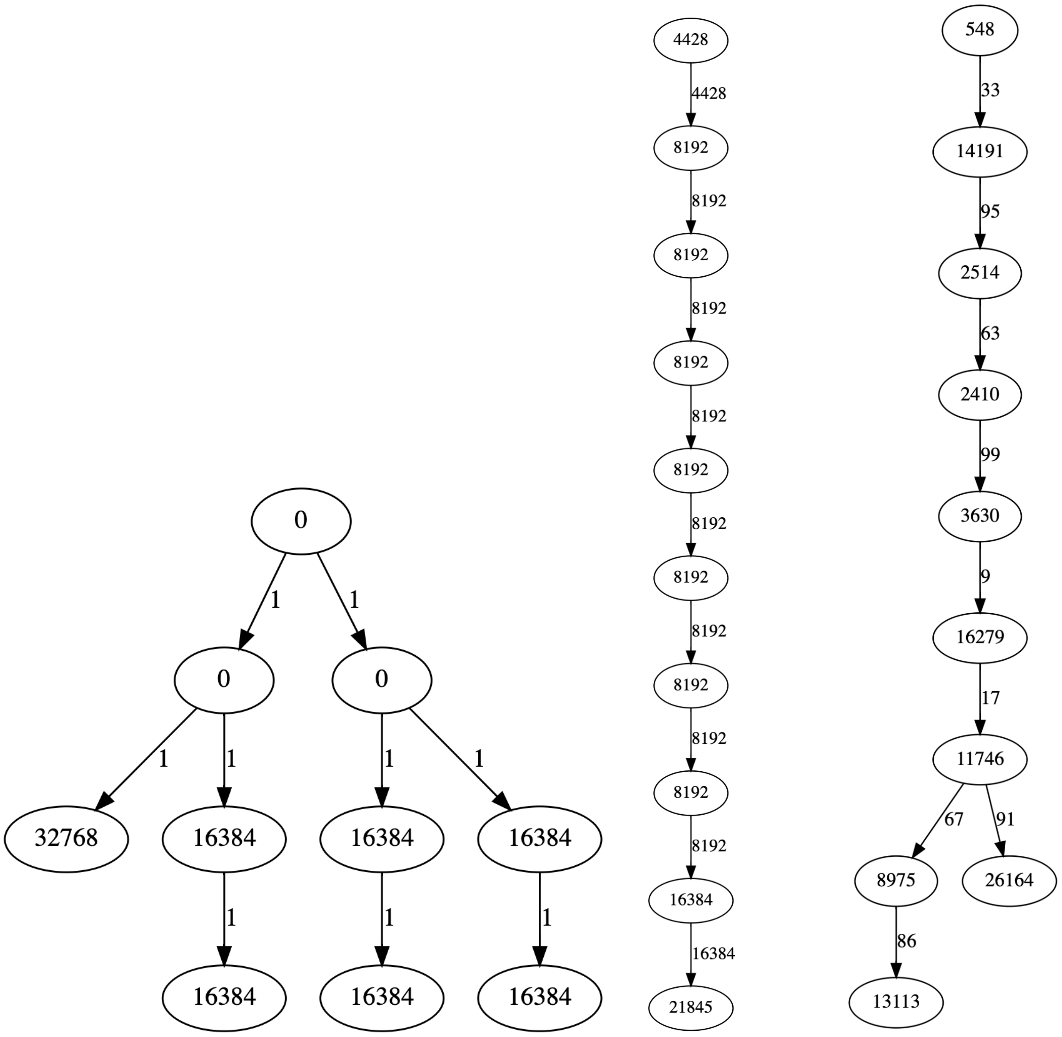

A **B** **C**

Figure S28. Example algorithmic clustering of three synthetic trees. Example synthetic mutation annotated trees are clustered into smaller trees with only 10 nodes. Nodes are annotated by the number of sequences represented by each cluster. Edges are annotated by the edit distance between clusters. (A) clusters a balanced binary tree of 262,143 nodes, (B) clusters a single linear chain of 20,001 nodes, (C) clusters a random binary tree with Geometric(½)-many children at each node and 200,000 nodes. In all examples the clustered trees are approximately balanced insofar as they exhibit narrow distributions of edge distances (in SNPs) and the cluster sizes (in genomes sampled).

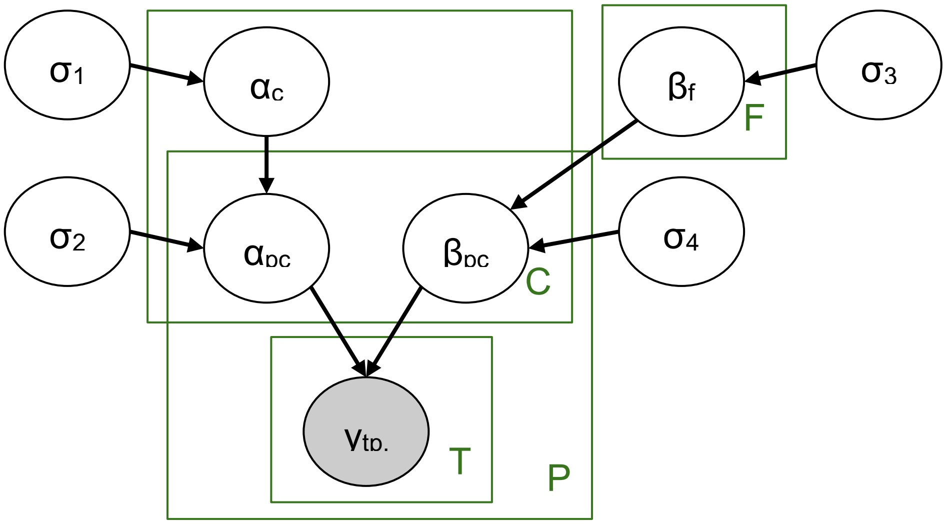

Figure S29. Probabilistic graphical model structure of the PyR_0 model. Variables σ are scale parameters of distributions. Variable β_f is the per-amino-acid-substitution fitness coefficient. Variables β_{pc} and α_{pc} are respectively the per-region per-cluster slope and intercepts parametrizing multivariate logistic growth curves. The mean parameter of β_{pc} is determined by β_f via matrix multiplication by the feature matrix X_{cf}. The mean parameter of α_{pc} is a per-cluster intercept α_c shared across regions. The multinomial observations are vectors $y_{tp.}$ each of whose entries y_{tpc} is the number of samples of cluster c in place p in time bucket t. Green boxes denote plates, i.e. conditionally independent replicas of random variables. Note the vector-valued observation $y_{tp.}$ is outside of the C plate because the multinomial distribution couples entries across the cluster coordinate c. Because the P x C plates are sparse (in most places most clusters never appear) the model omits α_{pc} and β_{pc} for pairs (p,c) with no observations in y.

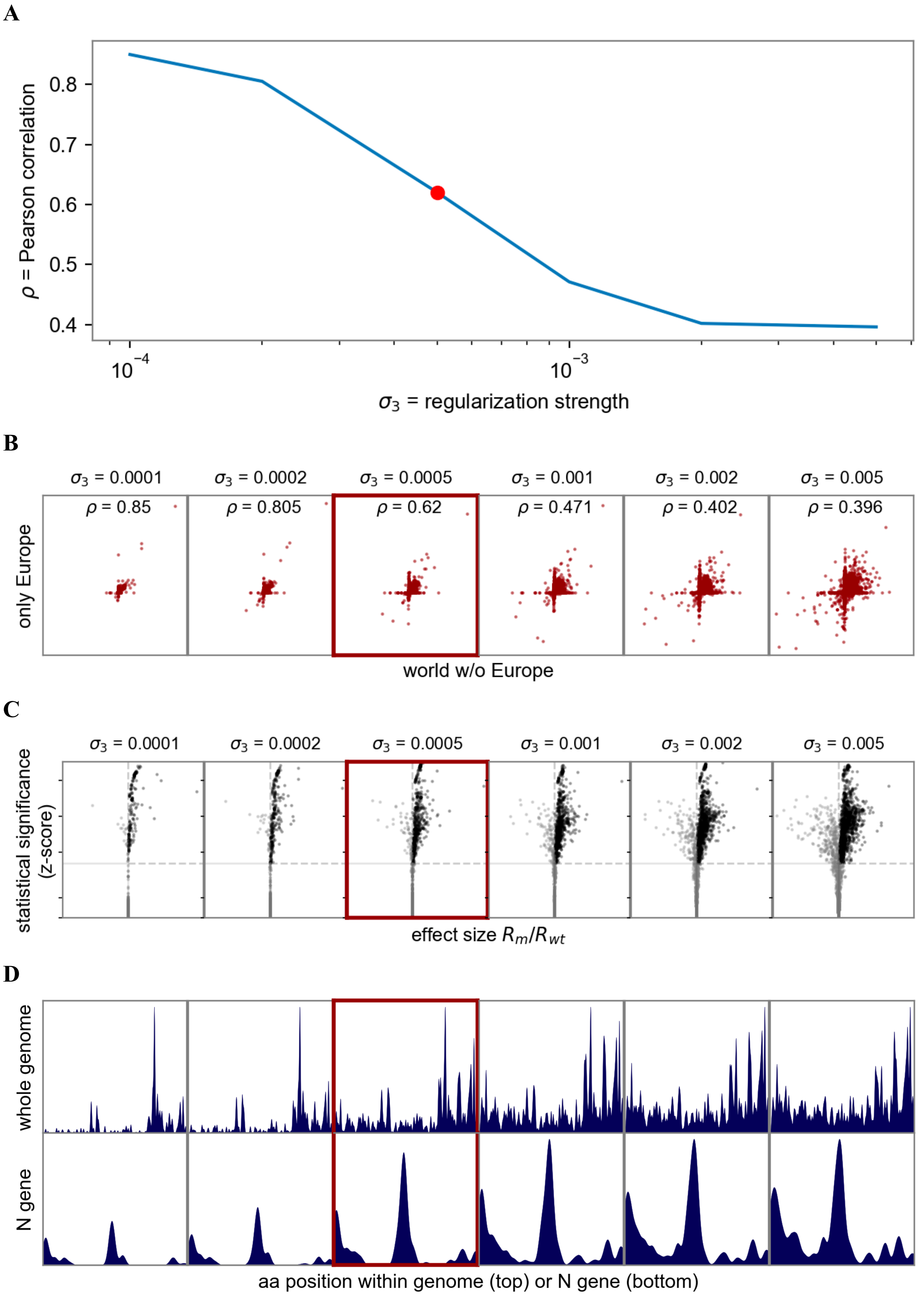
A
ρ = Pearson correlation
0.8
0.7
0.6
0.5
0.4
10^−4
10^−3
σ3 = regularization strength
B
σ3 = 0.0001
σ3 = 0.0002
σ3 = 0.0005
σ3 = 0.001
σ3 = 0.002
σ3 = 0.005
ρ = 0.85
ρ = 0.805
ρ = 0.62
ρ = 0.471
ρ = 0.402
ρ = 0.396
only Europe
world w/o Europe
C
σ3 = 0.0001
σ3 = 0.0002
σ3 = 0.0005
σ3 = 0.001
σ3 = 0.002
σ3 = 0.005
statistical significance (z-score)
effect size Rm/Rwt
D
whole genome
N gene
aa position within genome (top) or N gene (bottom)

E

σ_3=0.0001

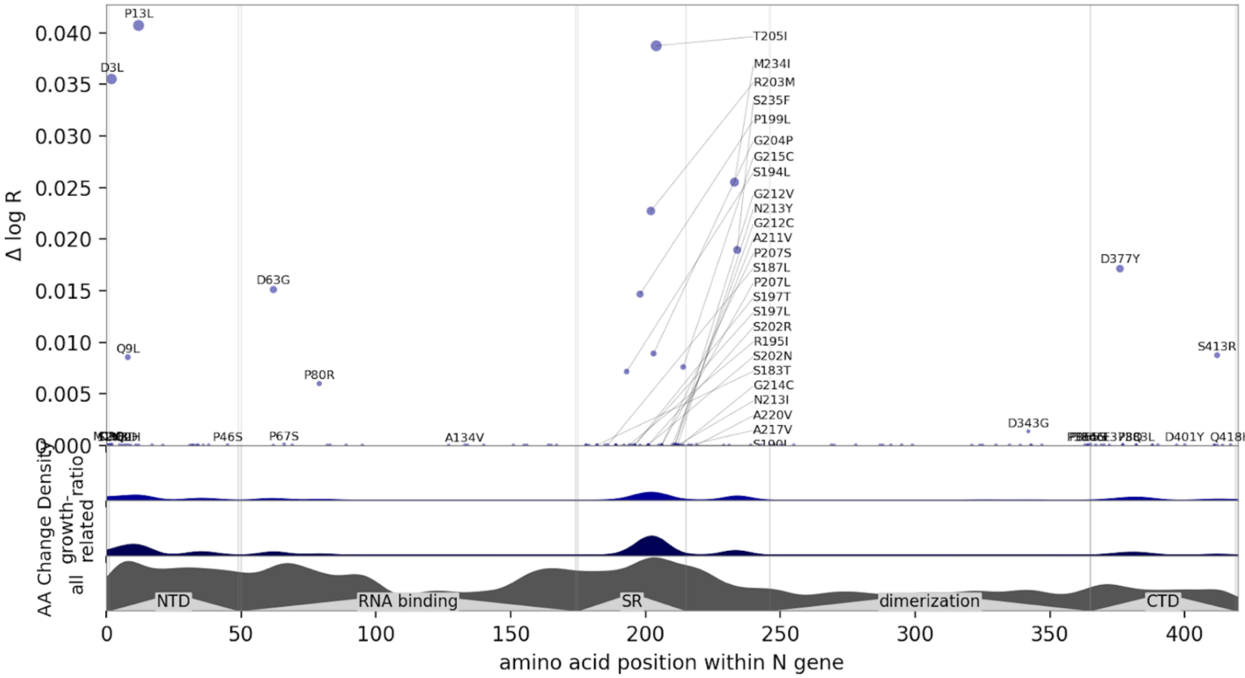

σ_3=0.0002

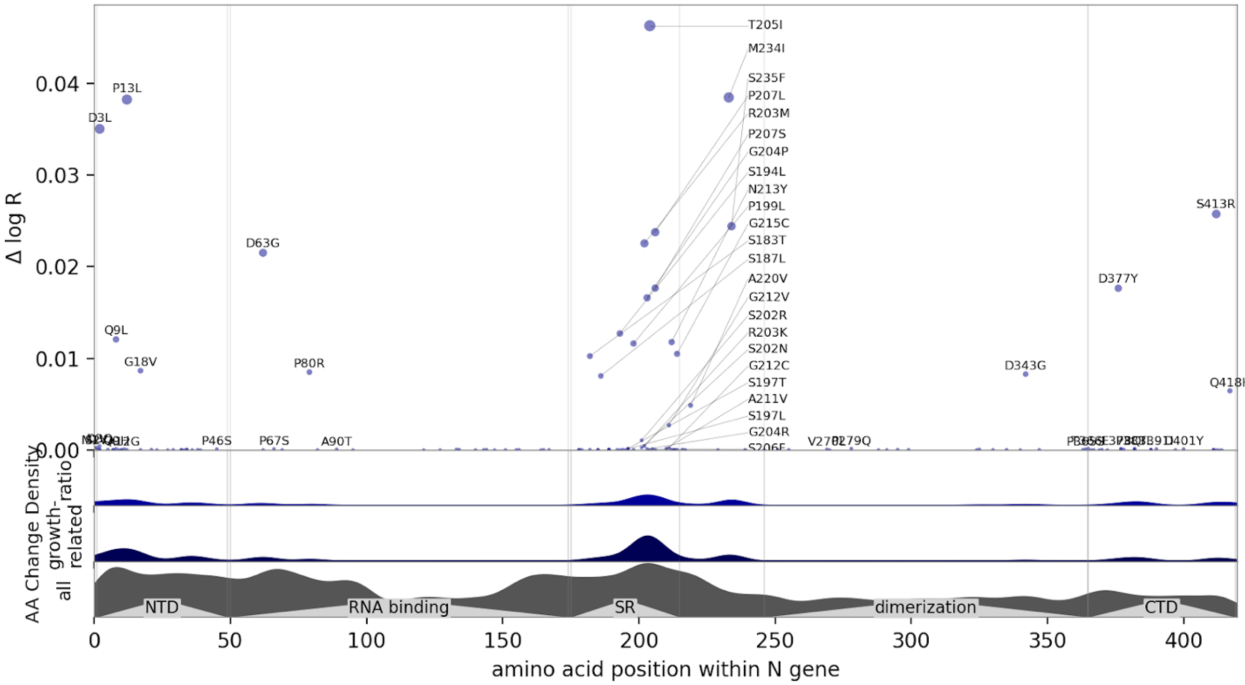

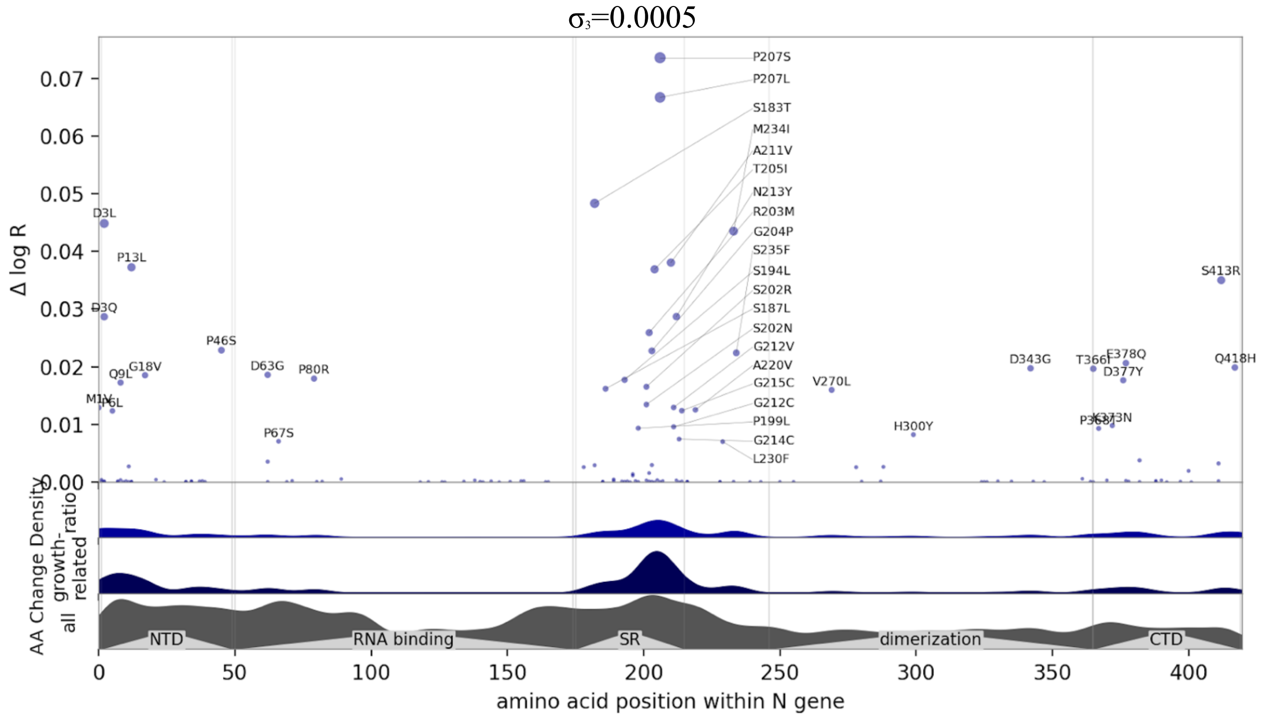

Figure S30. Validation experiment used to choose Laplace regularization strength σ_3. (A) Pearson correlation for the cross-validation analysis in panel B for six different values of σ_3. (B) Geographic cross-validation scatterplots similar to Fig. S19A but for different values of σ_3. (C) Volcano plots similar to Fig. S18 but for different values of σ_3. (D) Manhattan smoothed density plots of mutation–similar to the middle density plot of Fig. 2A. The chosen value σ_3=1/2000 is highlighted in red. (E) Manhattan plot for σ_3=0.0001 (top panel), σ_3=0.0002 (middle panel), σ_3=0.0005 (lower panel), with a magnified view of the window around position 200. Increasing values of σ_3 to σ_3=0.0005 (chosen model) resulted in a stronger signal in the linker region, shown by Syed et al.(22) to enhance packaging, and specifically recovered S202R, which the authors found had among the strongest effects in virus-like particles and was further validated in infectious virus.

A

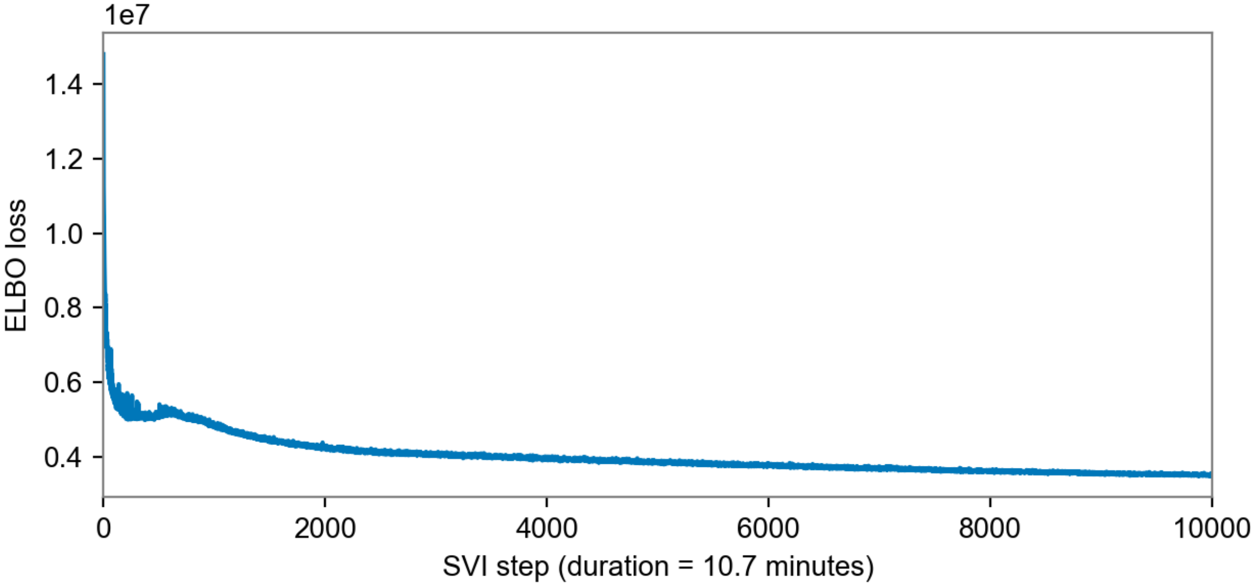

B

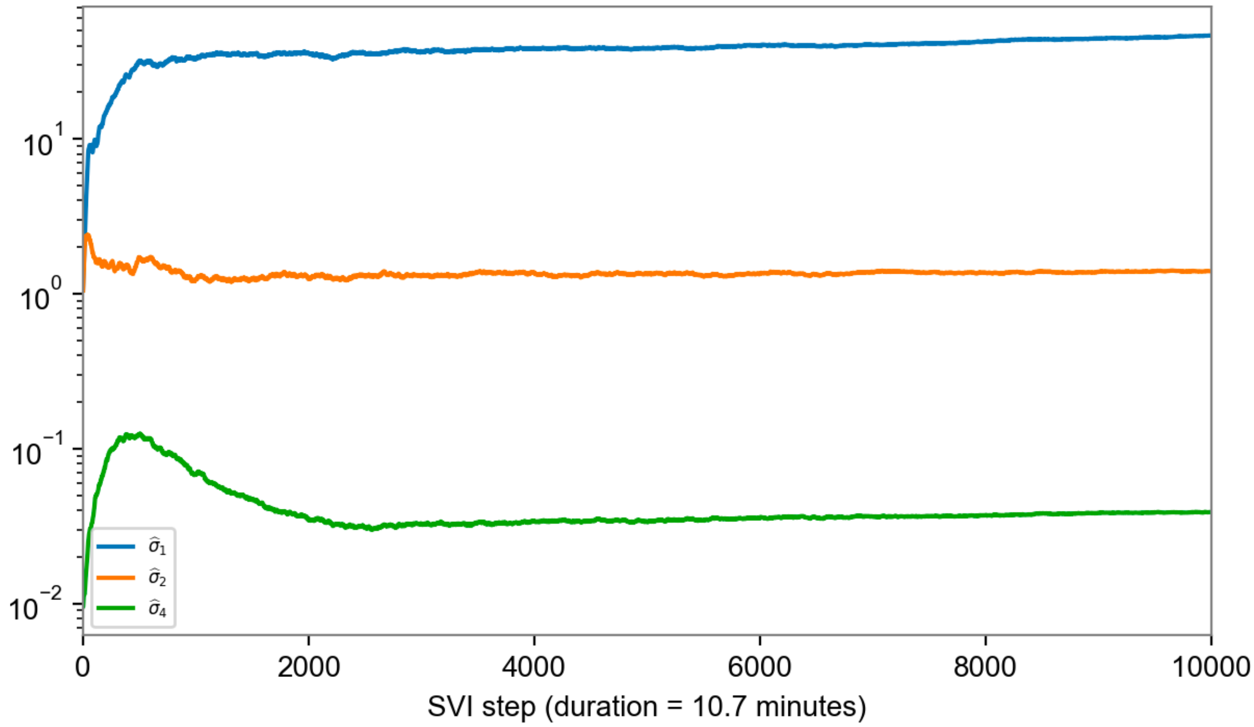

Figure S31. Convergence of variational inference algorithm. **A.** Convergence of ELBO loss. **B.** Convergence of posterior medians of scale parameters.

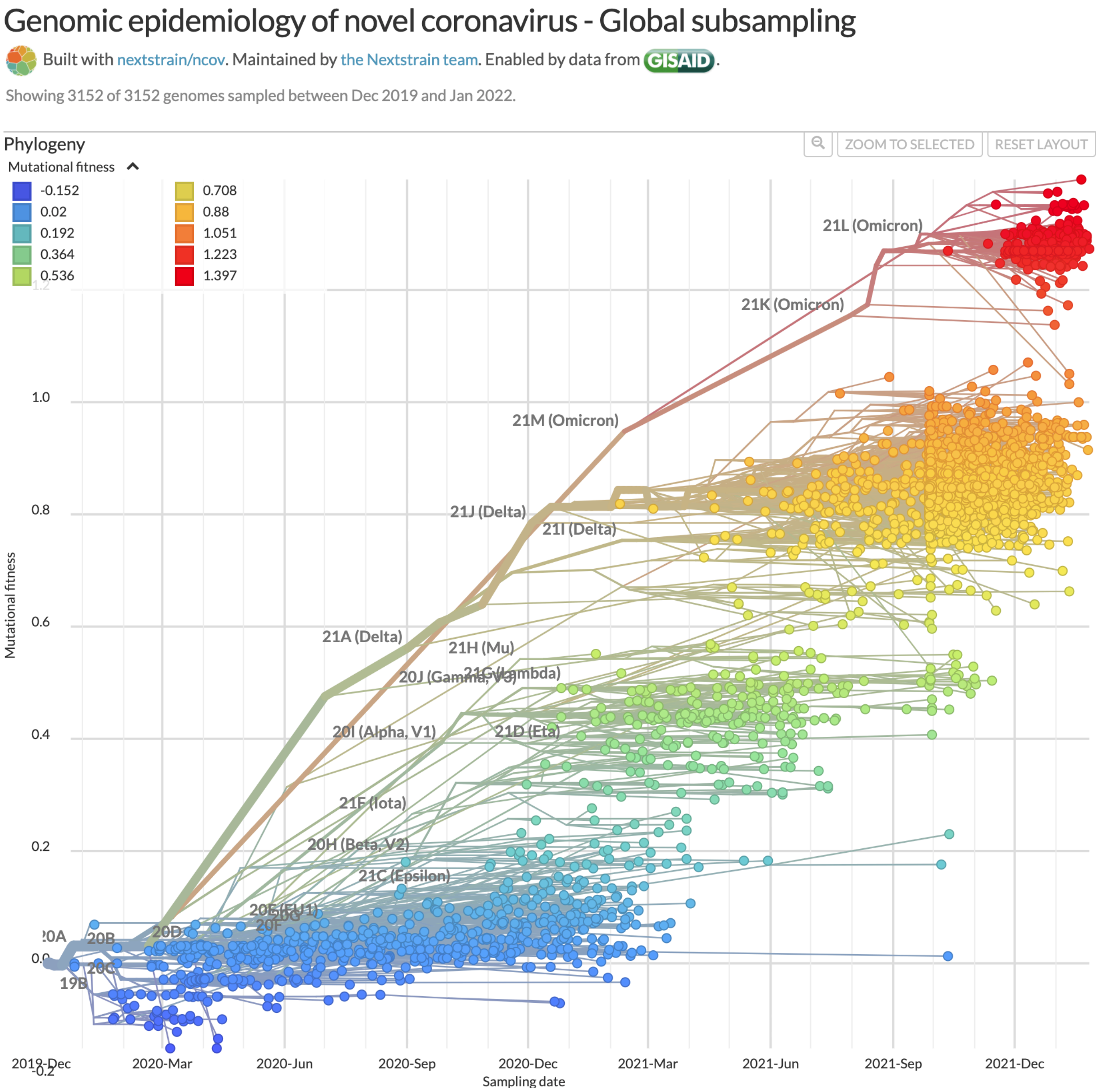

Figure S32: Screenshot of https://nextstrain.org displaying Nextstrain's subsampled phylogeny with color and y-axis (mutational fitness) determined by our model predicted Δ log R for each lineage (here using a slightly older version of our model). Although PyR$_0$ does not explicitly rely on phylogenetic information, fitness estimates vary smoothly across the phylogeny.

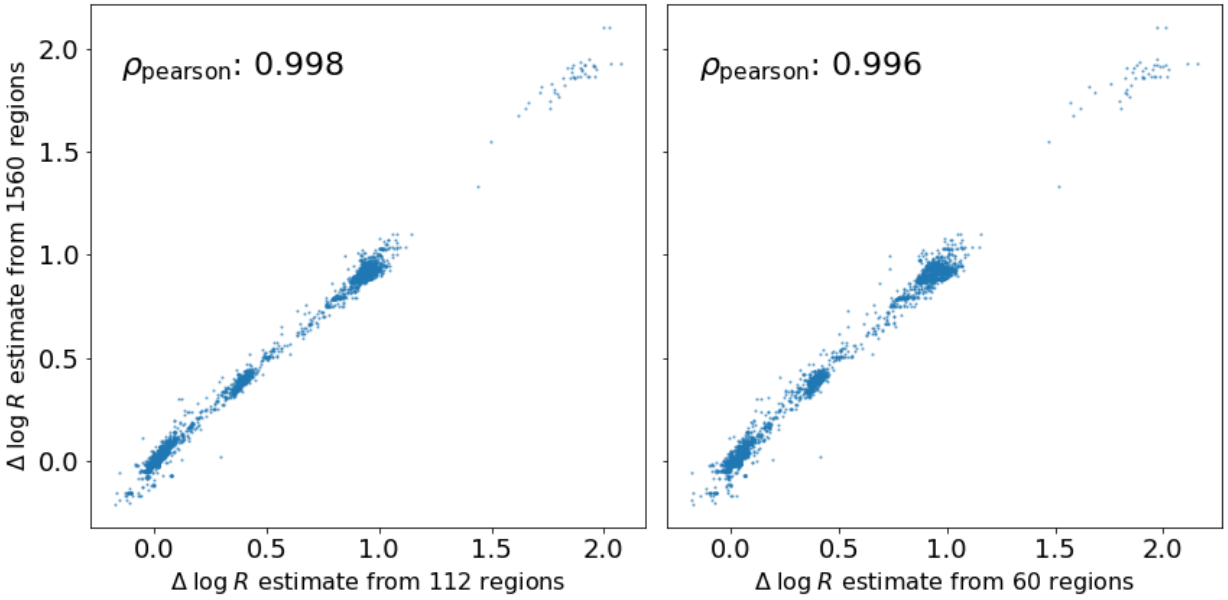

Figure S33. We show that PyR_0 lineage-level Δ log R estimates are largely driven by regions with the largest numbers of samples, as would be expected from a Bayesian hierarchical model. The vertical axis depicts Δ log R estimates based on all 1560 regions, while the horizontal axis on the left (respectively, right) depicts Δ log R estimates based on the 112 (60) regions with at least 10^4 (2×10^4) samples. Collectively these regions contain 80.7% (69.8%) of the total number of SARS-CoV-2 sequences in our full dataset.

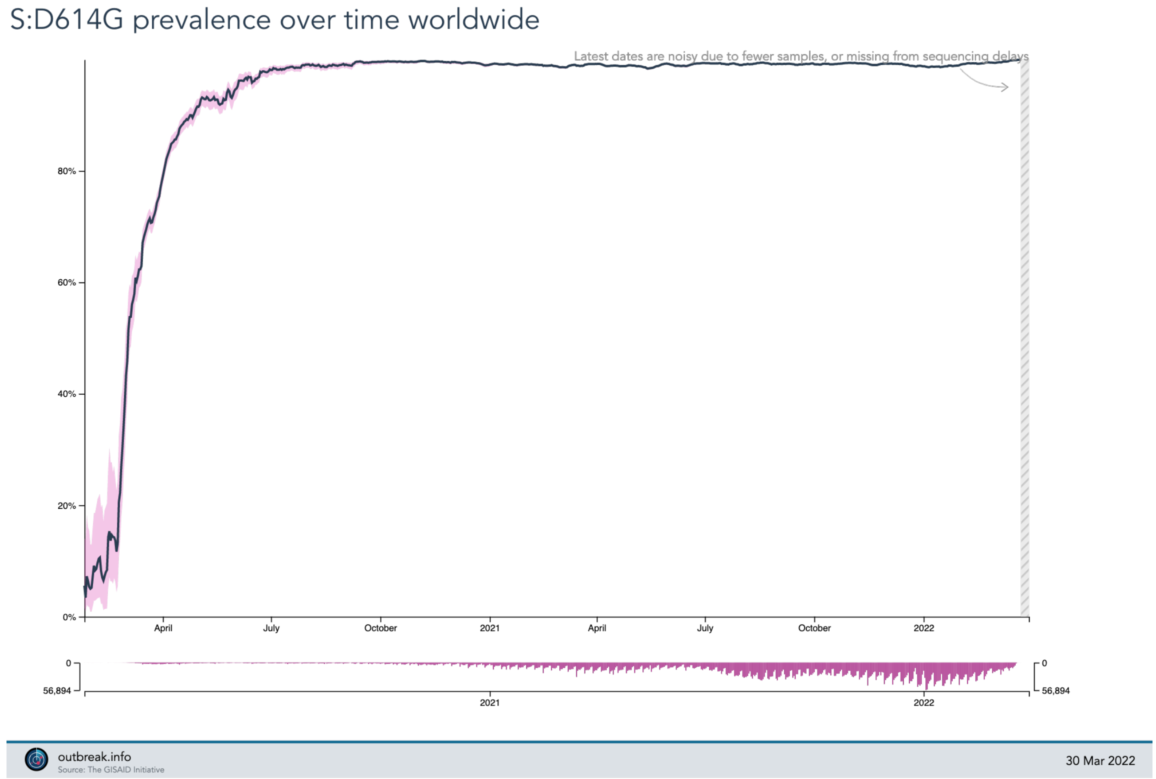

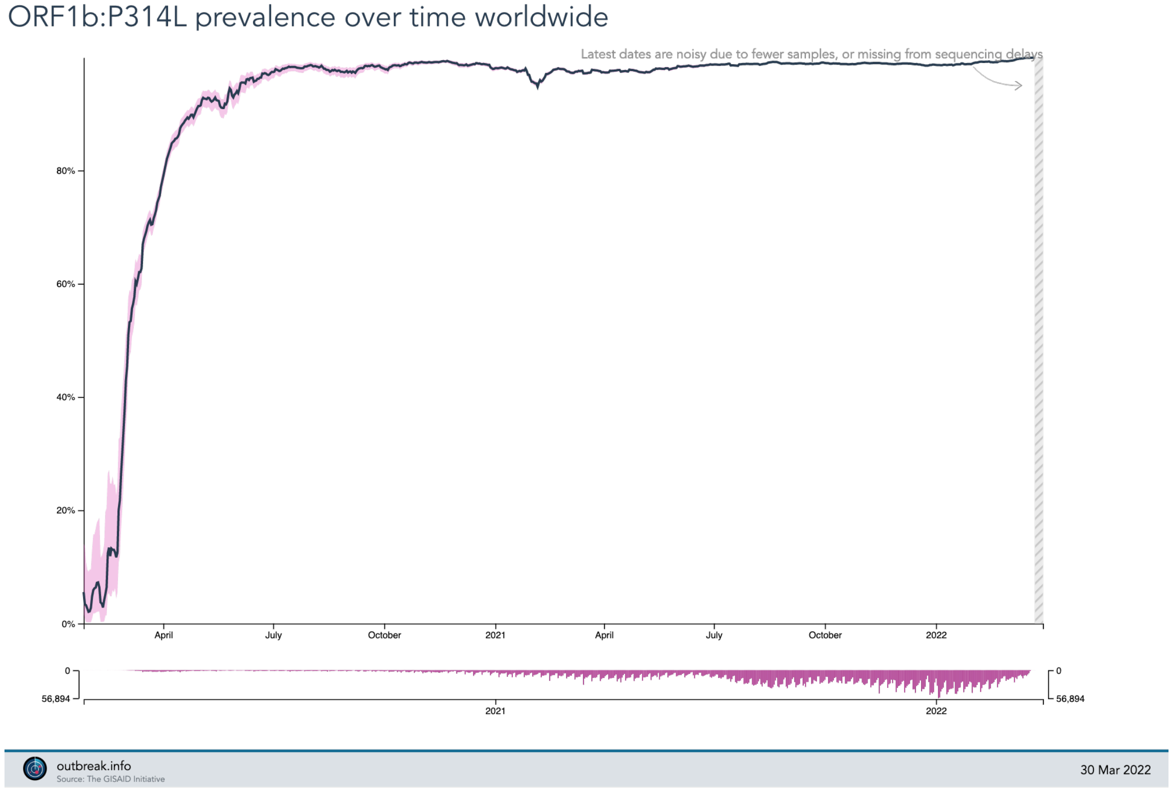

Figure S34. Prevalence of Spike D614G and ORF1b:P314L showing effective fixation beginning in July 2020. Data is from GISAID and plots are from Outbreak.info *(56)*.

Region	4-week forecast	8-week forecast
USA	82.9%	68.3%
France	75.6%	61.0%
England	75.6%	58.5%
Brazil	65.9%	51.2%
Australia	56.1%	39.0%
Russia	73.2%	68.3%

Table S1. Regional evaluation of forecasts. We evaluate the ability of PyR_0 to accurately forecast the dominant lineage 4- and 8-weeks into the future in six selected regions with a relatively large number of GISAID samples. Percentage accuracies are obtained by averaging over 45 training windows.

Spatial region	# of mutations	Extent of region (nt)	p-value	Lengthscale
Entire genome	2904	29394	0.000001	100
Entire genome	2904	29394	0.000001	500
S	415	3786	0.001910	50
N	220	1251	0.017627	50
ORF7a	75	360	0.024066	18
ORF3a	198	789	0.024307	39
ORF1a	1107	13182	0.029710	50
ORF7b	26	126	0.089589	6
ORF14	69	213	0.112527	11
ORF6	19	177	0.138634	9
ORF1b	552	8052	0.329416	50
E	17	195	0.455606	10
M	42	639	0.518497	32

Table S2. Spatial structure of the inferred amino acid coefficients β_f. We report one-sided p-values for the Moran I spatial autocorrelation statistic computed using a permutation test. We use a gaussian weighting function of the form $\exp(-\text{distance}^2/\text{lengthscale}^2)$, where distance is measured in units of nucleotides. We find that there is significant evidence for spatial structure in S, N, ORF7a, ORF3a, and ORF1a as well as across the SARS-CoV-2 genome as a whole.

Open Reading Frame (ORF)	Rank	Mutation	Fold Increase in Transmissibility	Number of Lineages
Nucleocapsid				
	11	P13L	1.04	26
	42	R203M	1.03	214
	47	D63G	1.02	209
	48	G215C	1.02	152
	57	D377Y	1.02	228
	70	D3L	1.04	13
	82	Q9L	1.02	8
	83	S413R	1.05	3
	91	S235F	1.02	15
ORF1b				
	21	I1566V	1.04	6
	43	P1000L	1.02	221
	53	G662S	1.02	212
	58	A1918V	1.02	152
	71	L829I	1.02	9
	72	T2163I	1.08	3
	75	I1257V	1.03	3
	78	H1087Y	1.02	5
	80	T1540I	1.04	6
Spike				
	1	H655Y	1.05	34
	2	T95I	1.05	31
	4	N764K	1.04	7
	6	S371L	1.04	4
	8	Q954H	1.04	6
	10	L981F	1.04	3
	12	G339D	1.04	5
	13	S375F	1.04	6
	14	S477N	1.04	48

Table S3. Table of the most statistically significant mutations in spike, ORF1b, and nucleocapsid. The top 9 mutations for each of the listed ORFs is shown. Mutations such as S:H655, S:T95I, and N:R203M have emerged independently in VoC lineages.

Rank	Gene	Substitution	R/R_A
1	ORF1b	P314L	1.18596
2	S	D614G	1.13656
3	S	P681H	1.05378
4	S	N501Y	1.04264
5	S	Y144-	1.02837
6	ORF8	L84S	1.26443
7	S	P681R	1.05085
8	ORF1a	D448-	1.35223
9	N	S235F	1.02428
10	ORF1a	G3676-	1.04243
11	ORF1a	S3675-	1.04178
12	ORF3a	S26L	1.03814
13	M	I82T	1.0369
14	ORF7a	V82A	1.03684
15	S	V70-	1.02043
16	N	D3L	1.01791
17	N	D377Y	1.04436
18	ORF3a	G251V	1.24599
19	ORF8	R52I	1.01815
20	S	D950N	1.0349

Table S4: Top-20 mutations from an earlier model (see methods) run on 2.1 million genomes downloaded from GISAID July 2021.

**PANGO Lineage**	**WHO Classification**
B.1.1.7	Alpha
B.1.351	Beta
B.1.617.2, AY.x	Delta
B.1.427, B.1.429	Epsilon
P.1	Gamma
P.2	Zeta
P.3	Theta
B.1.621	Mu
B.1.526	Iota
BA.1, BA.2, BA.3, B.1.1.529	Omicron

Table S5. Data dictionary mapping PANGO lineages to WHO classifications.

Data S1. (separate file strains.tsv)
Complete list of PANGO lineages with inferred relative fitness, ranked by fitness.
Mirrored at https://github.com/broadinstitute/pyro-cov/blob/v0.2/paper/strains.tsv

Data S2. (separate file mutations.tsv)
Complete list of amino acid changes with inferred effect size, ranked by z-score.
Mirrored at https://github.com/broadinstitute/pyro-cov/blob/v0.2/paper/mutations.tsv

Data S3. (separate file accession_ids.txt.xz)
Complete list of GISAID accession numbers of viral genomes used in this study.
Mirrored at https://github.com/broadinstitute/pyro-cov/blob/v0.2/paper/accession_ids.txt.xz

Data S4. (separate file strains_july_2021_model.tsv)
Complete list of PANGO lineages with inferred relative fitness, ranked by fitness, from 2.1 million genomes model fit in July 2021.

Data S5. (separate file mutations_july_2021_model.tsv)
Complete list of amino acid changes with inferred effect size, ranked by z-score, from 2.1 million genomes model fit in July 2021.

1. N. G. Davies, S. Abbott, R. C. Barnard, C. I. Jarvis, A. J. Kucharski, J. D. Munday, C. A. B. Pearson, T. W. Russell, D. C. Tully, A. D. Washburne, T. Wenseleers, A. Gimma, W. Waites, K. L. M. Wong, K. van Zandvoort, J. D. Silverman, K. Diaz-Ordaz, R. Keogh, R. M. Eggo, S. Funk, M. Jit, K. E. Atkins, W. J. Edmunds; CMMID COVID-19 Working Group; COVID-19 Genomics UK (COG-UK) Consortium, Estimated transmissibility and impact of SARS-CoV-2 lineage B.1.1.7 in England. *Science* **372**, eabg3055 (2021). doi:10.1126/science.abg3055 Medline

2. E. Volz, S. Mishra, M. Chand, J. C. Barrett, R. Johnson, L. Geidelberg, W. R. Hinsley, D. J. Laydon, G. Dabrera, Á. O'Toole, R. Amato, M. Ragonnet-Cronin, I. Harrison, B. Jackson, C. V. Ariani, O. Boyd, N. J. Loman, J. T. McCrone, S. Gonçalves, D. Jorgensen, R. Myers, V. Hill, D. K. Jackson, K. Gaythorpe, N. Groves, J. Sillitoe, D. P. Kwiatkowski, S. Flaxman, O. Ratmann, S. Bhatt, S. Hopkins, A. Gandy, A. Rambaut, N. M. Ferguson; COVID-19 Genomics UK (COG-UK) consortium, Assessing transmissibility of SARS-CoV-2 lineage B.1.1.7 in England. *Nature* **593**, 266–269 (2021). Medline

3. P. Stefanelli, F. Trentini, G. Guzzetta, V. Marziano, A. Mammone, P. Poletti, C. M. Grané, M. Manica, M. del Manso, X. Andrianou, Others, Co-circulation of SARS-CoV-2 variants B. 1.1. 7 and P. 1. *medRxiv* (2021) (available at https://www.medrxiv.org/content/10.1101/2021.04.06.21254923v1.abstract).

4. P. Stefanelli, F. Trentini, G. Guzzetta, V. Marziano, A. Mammone, M. Sane Schepisi, P. Poletti, C. Molina Grané, M. Manica, M. Del Manso, X. Andrianou, M. Ajelli, G. Rezza, S. Brusaferro, S. Merler; COVID-19 National Microbiology Surveillance Study Group, Co-circulation of SARS-CoV-2 Alpha and Gamma variants in Italy, February and March 2021. *Euro Surveill.* **27**, (2022). doi:10.2807/1560-7917.ES.2022.27.5.2100429 Medline

5. H. S. Vöhringer, T. Sanderson, M. Sinnott, N. De Maio, T. Nguyen, R. Goater, F. Schwach, I. Harrison, J. Hellewell, C. V. Ariani, S. Gonçalves, D. K. Jackson, I. Johnston, A. W. Jung, C. Saint, J. Sillitoe, M. Suciu, N. Goldman, J. Panovska-Griffiths, E. Birney, E. Volz, S. Funk, D. Kwiatkowski, M. Chand, I. Martincorena, J. C. Barrett, M. Gerstung; Wellcome Sanger Institute COVID-19 Surveillance Team; COVID-19 Genomics UK (COG-UK) Consortium*, Genomic reconstruction of the SARS-CoV-2 epidemic in England. *Nature* **600**, 506–511 (2021). Medline

6. B. Korber, W. M. Fischer, S. Gnanakaran, H. Yoon, J. Theiler, W. Abfalterer, N. Hengartner, E. E. Giorgi, T. Bhattacharya, B. Foley, K. M. Hastie, M. D. Parker, D. G. Partridge, C. M. Evans, T. M. Freeman, T. I. de Silva, C. McDanal, L. G. Perez, H. Tang, A. Moon-Walker, S. P. Whelan, C. C. LaBranche, E. O. Saphire, D. C. Montefiori, A. Angyal, R. L. Brown, L. Carrilero, L. R. Green, D. C. Groves, K. J. Johnson, A. J. Keeley, B. B. Lindsey, P. J. Parsons, M. Raza, S. Rowland-Jones, N. Smith, R. M. Tucker, D. Wang, M. D. Wyles; Sheffield COVID-19 Genomics Group, Tracking Changes in SARS-CoV-2 Spike: Evidence that D614G Increases Infectivity of the COVID-19 Virus. *Cell* **182**, 812–827.e19 (2020). doi:10.1016/j.cell.2020.06.043 Medline

7. L. Yurkovetskiy, X. Wang, K. E. Pascal, C. Tomkins-Tinch, T. P. Nyalile, Y. Wang, A. Baum, W. E. Diehl, A. Dauphin, C. Carbone, K. Veinotte, S. B. Egri, S. F. Schaffner, J. E. Lemieux, J. B. Munro, A. Rafique, A. Barve, P. C. Sabeti, C. A. Kyratsous, N. V. Dudkina, K. Shen, J. Luban, Structural and Functional Analysis of the D614G SARS-CoV-2 Spike Protein Variant. *Cell* **183**, 739–751.e8 (2020). doi:10.1016/j.cell.2020.09.032 Medline

8. X. Deng, M. A. Garcia-Knight, M. M. Khalid, V. Servellita, C. Wang, M. K. Morris, A. Sotomayor-González, D. R. Glasner, K. R. Reyes, A. S. Gliwa, N. P. Reddy, C. Sanchez San Martin, S. Federman, J. Cheng, J. Balcerek, J. Taylor, J. A. Streithorst, S. Miller, B. Sreekumar, P.-Y. Chen, U. Schulze-Gahmen, T. Y. Taha, J. M. Hayashi, C. R. Simoneau, G. R. Kumar, S. McMahon, P. V. Lidsky, Y. Xiao, P. Hemarajata, N. M. Green, A. Espinosa, C. Kath, M. Haw, J. Bell, J. K. Hacker, C. Hanson, D. A. Wadford, C. Anaya, D. Ferguson, P. A. Frankino, H. Shivram, L. F. Lareau, S. K. Wyman, M. Ott, R. Andino, C. Y. Chiu, Transmission, infectivity, and neutralization of a spike L452R SARS-CoV-2 variant. *Cell* **184**, 3426–3437.e8 (2021). doi:10.1016/j.cell.2021.04.025 Medline

9. T. N. Starr, A. J. Greaney, S. K. Hilton, D. Ellis, K. H. D. Crawford, A. S. Dingens, M. J. Navarro, J. E. Bowen, M. A. Tortorici, A. C. Walls, N. P. King, D. Veesler, J. D. Bloom, Deep Mutational Scanning of SARS-CoV-2 Receptor Binding Domain Reveals Constraints on Folding and ACE2 Binding. *Cell* **182**, 1295–1310.e20 (2020). doi:10.1016/j.cell.2020.08.012 Medline

10. B. Choi, M. C. Choudhary, J. Regan, J. A. Sparks, R. F. Padera, X. Qiu, I. H. Solomon, H.-H. Kuo, J. Boucau, K. Bowman, U. D. Adhikari, M. L. Winkler, A. A. Mueller, T. Y.-T. Hsu, M. Desjardins, L. R. Baden, B. T. Chan, B. D. Walker, M. Lichterfeld, M. Brigl, D. S. Kwon, S. Kanjilal, E. T. Richardson, A. H. Jonsson, G. Alter, A. K. Barczak, W. P. Hanage, X. G. Yu, G. D. Gaiha, M. S. Seaman, M. Cernadas, J. Z. Li, Persistence and Evolution of SARS-CoV-2 in an Immunocompromised Host. *N. Engl. J. Med.* **383**, 2291–2293 (2020). doi:10.1056/NEJMc2031364 Medline

11. A. J. Greaney, T. N. Starr, P. Gilchuk, S. J. Zost, E. Binshtein, A. N. Loes, S. K. Hilton, J. Huddleston, R. Eguia, K. H. D. Crawford, A. S. Dingens, R. S. Nargi, R. E. Sutton, N. Suryadevara, P. W. Rothlauf, Z. Liu, S. P. J. Whelan, R. H. Carnahan, J. E. Crowe Jr., J. D. Bloom, Complete Mapping of Mutations to the SARS-CoV-2 Spike Receptor-Binding Domain that Escape Antibody Recognition. *Cell Host Microbe* **29**, 44–57.e9 (2021). doi:10.1016/j.chom.2020.11.007 Medline

12. A. Rambaut, E. C. Holmes, Á. O'Toole, V. Hill, J. T. McCrone, C. Ruis, L. du Plessis, O. G. Pybus, A dynamic nomenclature proposal for SARS-CoV-2 lineages to assist genomic epidemiology. *Nat. Microbiol.* **5**, 1403–1407 (2020). doi:10.1038/s41564-020-0770-5 Medline

13. H. S. Vöhringer, T. Sanderson, M. Sinnott, N. De Maio, T. Nguyen, R. Goater, F. Schwach, I. Harrison, J. Hellewell, C. Ariani, S. Gonçalves, D. Jackson, I. Johnston, A. W. Jung, C. Saint, J. Sillitoe, M. Suciu, N. Goldman, E. Birney, S. Funk, E. Volz, D. Kwiatkowski, M. Chand, I. Martincorena, J. C. Barrett, M. Gerstung, The Wellcome Sanger Institute Covid-19 Surveillance Team, The COVID-19 Genomics UK (COG-UK) Consortium, Genomic reconstruction of the SARS-CoV-2 epidemic across England from September 2020 to May 2021*bioRxiv* (2021), doi:10.1101/2021.05.22.21257633.

14. F. Campbell, B. Archer, H. Laurenson-Schafer, Y. Jinnai, F. Konings, N. Batra, B. Pavlin, K. Vandemaele, M. D. Van Kerkhove, T. Jombart, O. Morgan, O. le Polain de Waroux, Increased transmissibility and global spread of SARS-CoV-2 variants of concern as at June 2021. *Euro Surveill.* **26**, (2021). doi:10.2807/1560-7917.ES.2021.26.24.2100509 Medline

15. S. Elbe, G. Buckland-Merrett, Data, disease and diplomacy: GISAID's innovative contribution to global health. *Glob. Chall.* **1**, 33–46 (2017). doi:10.1002/gch2.1018 Medline

16. E. Bingham, J. P. Chen, M. Jankowiak, F. Obermeyer, N. Pradhan, T. Karaletsos, R. Singh, P. Szerlip, P. Horsfall, N. D. Goodman, Pyro: Deep universal probabilistic programming. *J. Mach. Learn. Res.* **20**, 973–978 (2019).

17. F. Obermeyer, S. F. Schaffner, M. Jankowiak, N. Barkas, J. D. Pyle, D. J. Park, B. L. MacInnis, J. Luban, P. C. Sabeti, J. E. Lemieux, Analysis of 2.1 million SARS-CoV-2 genomes identifies mutations associated with transmissibility*medRxiv* (2021), doi:10.1101/2021.09.07.21263228.

18. Preliminary genomic characterisation of an emergent SARS-CoV-2 lineage in the UK defined by a novel set of spike mutations*Virological* (2020) (available at https://virological.org/t/preliminary-genomic-characterisation-of-an-emergent-sars-cov-2-lineage-in-the-uk-defined-by-a-novel-set-of-spike-mutations/563).

19. R. Viana, S. Moyo, D. G. Amoako, H. Tegally, C. Scheepers, C. L. Althaus, U. J. Anyaneji, P. A. Bester, M. F. Boni, M. Chand, W. T. Choga, R. Colquhoun, M. Davids, K. Deforche, D. Doolabh, L. du Plessis, S. Engelbrecht, J. Everatt, J. Giandhari, M. Giovanetti, D. Hardie, V. Hill, N.-Y. Hsiao, A. Iranzadeh, A. Ismail, C. Joseph, R. Joseph, L. Koopile, S. L. Kosakovsky Pond, M. U. G. Kraemer, L. Kuate-Lere, O. Laguda-Akingba, O. Lesetedi-Mafoko, R. J. Lessells, S. Lockman, A. G. Lucaci, A. Maharaj, B. Mahlangu, T. Maponga, K. Mahlakwane, Z. Makatini, G. Marais, D. Maruapula, K. Masupu, M. Matshaba, S. Mayaphi, N. Mbhele, M. B. Mbulawa, A. Mendes, K. Mlisana, A. Mnguni, T. Mohale, M. Moir, K. Moruisi, M. Mosepele, G. Motsatsi, M. S. Motswaledi, T. Mphoyakgosi, N. Msomi, P. N. Mwangi, Y. Naidoo, N. Ntuli, M. Nyaga, L. Olubayo, S. Pillay, B. Radibe, Y. Ramphal, U. Ramphal, J. E. San, L. Scott, R. Shapiro, L. Singh, P. Smith-Lawrence, W. Stevens, A. Strydom, K. Subramoney, N. Tebeila, D. Tshiabuila, J. Tsui, S. van Wyk, S. Weaver, C. K. Wibmer, E. Wilkinson, N. Wolter, A. E. Zarebski, B. Zuze, D. Goedhals, W. Preiser, F. Treurnicht, M. Venter, C. Williamson, O. G. Pybus, J. Bhiman, A. Glass, D. P. Martin, A. Rambaut, S. Gaseitsiwe, A. von Gottberg, T. de Oliveira, Rapid epidemic expansion of the SARS-CoV-2 Omicron variant in southern Africa. *Nature* **603**, 679–686 (2022). doi:10.1038/s41586-022-04411-y Medline

20. A. J. Greaney, T. N. Starr, J. D. Bloom, An antibody-escape estimator for mutations to the SARS-CoV-2 receptor-binding domain. *Virus Evol.* **8**, veac021 (2022). doi:10.1093/ve/veac021 Medline

21. J. Yu, A. Y. Collier, M. Rowe, F. Mardas, J. D. Ventura, H. Wan, J. Miller, O. Powers, B. Chung, M. Siamatu, N. P. Hachmann, N. Surve, F. Nampanya, A. Chandrashekar, D. H. Barouch, Neutralization of the SARS-CoV-2 omicron BA.1 and BA.2 variants. *N. Engl. J. Med.* **386**, 1579–1580 (2022). doi:10.1056/NEJMc2201849 Medline

22. A. M. Syed, T. Y. Taha, T. Tabata, I. P. Chen, A. Ciling, M. M. Khalid, B. Sreekumar, P.-Y. Chen, J. M. Hayashi, K. M. Soczek, M. Ott, J. A. Doudna, Rapid assessment of SARS-CoV-2-evolved variants using virus-like particles. *Science* **374**, 1626–1632 (2021). doi:10.1126/science.abl6184 Medline

23. L. Ferretti, A. Ledda, C. Wymant, L. Zhao, V. Ledda, L. Abeler-Dörner, M. Kendall, A. Nurtay, H.-Y. Cheng, T.-C. Ng, H.-H. Lin, R. Hinch, J. Masel, A. M. Kilpatrick, C. Fraser, The timing of COVID-19 transmission*bioRxiv* (2020), doi:10.1101/2020.09.04.20188516.

24. *broadinstitute/pyro-cov: v0.2.1* (2022; https://zenodo.org/record/6399987).

25. Y. Turakhia, B. Thornlow, A. S. Hinrichs, N. De Maio, L. Gozashti, R. Lanfear, D. Haussler, R. Corbett-Detig, Ultrafast Sample placement on Existing tRees (UShER) enables real-time phylogenetics for the SARS-CoV-2 pandemic. *Nat. Genet.* **53**, 809–816 (2021). doi:10.1038/s41588-021-00862-7 Medline

26. J. McBroome, B. Thornlow, A. S. Hinrichs, A. Kramer, N. De Maio, N. Goldman, D. Haussler, R. Corbett-Detig, Y. Turakhia, A daily-updated database and tools for comprehensive SARS-CoV-2 mutation-annotated trees. *Mol. Biol. Evol.* **38**, 5819–5824 (2021). doi:10.1093/molbev/msab264 Medline

27. S. Nersisyan, A. Zhiyanov, M. Shkurnikov, A. Tonevitsky, T-CoV: a comprehensive portal of HLA-peptide interactions affected by SARS-CoV-2 mutations*bioRxiv*, 2021.07.06.451227 (2021).

28. J. F. Crow, M. and Kimura, *An Introduction to Population Genetics Theory* (The Blackburn Press, 1970).

29. T. A. Hopf, C. P. I. Schärfe, J. P. G. L. M. Rodrigues, A. G. Green, O. Kohlbacher, C. Sander, A. M. J. J. Bonvin, D. S. Marks, Sequence co-evolution gives 3D contacts and structures of protein complexes. *eLife* **3**, e03430 (2014). doi:10.7554/eLife.03430 Medline

30. J. Frazer, P. Notin, M. Dias, A. Gomez, J. K. Min, K. Brock, Y. Gal, D. S. Marks, Disease variant prediction with deep generative models of evolutionary data. *Nature* **599**, 91–95 (2021). doi:10.1038/s41586-021-04043-8 Medline

31. A. Paszke, S. Gross, S. Chintala, G. Chanan, E. Yang, Z. DeVito, Z. Lin, A. Desmaison, L. Antiga, A. Lerer, Automatic differentiation in PyTorch (2017) (available at https://openreview.net/pdf?id=BJJsrmfCZ).

32. M. Gorinova, D. Moore, M. Hoffman, in *Proceedings of the 37th International Conference on Machine Learning*, Proceedings of Machine Learning Research. H. D. Iii, A. Singh, Eds. (PMLR, 2020), vol. 119, pp. 3648–3657.

33. R. M. Neal, Slice sampling. *Ann. Stat.* **31**, (2003). doi:10.1214/aos/1056562461

34. D. P. Kingma, J. Ba, Adam: A Method for Stochastic Optimization*arXiv [cs.LG]* (2014) (available at https://arxiv.org/abs/1412.6980).

35. L. Cappello, J. Kim, S. Liu, J. A. Palacios, Statistical Challenges in Tracking the Evolution of SARS-CoV-2*arXiv [stat.AP]* (2021) (available at https://arxiv.org/abs/2108.13362).

36. Y. Cao, J. Wang, F. Jian, T. Xiao, W. Song, A. Yisimayi, W. Huang, Q. Li, P. Wang, R. An, J. Wang, Y. Wang, X. Niu, S. Yang, H. Liang, H. Sun, T. Li, Y. Yu, Q. Cui, S. Liu, X. Yang, S. Du, Z. Zhang, X. Hao, F. Shao, R. Jin, X. Wang, J. Xiao, Y. Wang, X. S. Xie, Omicron escapes the majority of existing SARS-CoV-2 neutralizing antibodies. *Nature* **602**, 657–663 (2022). doi:10.1038/s41586-021-04385-3 Medline

37. D. Planas, N. Saunders, P. Maes, F. Guivel-Benhassine, C. Planchais, J. Buchrieser, W.-H. Bolland, F. Porrot, I. Staropoli, F. Lemoine, H. Péré, D. Veyer, J. Puech, J. Rodary, G. Baele, S. Dellicour, J. Raymenants, S. Gorissen, C. Geenen, B. Vanmechelen, T. Wawina-Bokalanga, J. Martí-Carreras, L. Cuypers, A. Sève, L. Hocqueloux, T. Prazuck, F. A. Rey, E. Simon-Loriere, T. Bruel, H. Mouquet, E. André, O. Schwartz, Considerable escape of SARS-CoV-2 Omicron to antibody neutralization. *Nature* **602**, 671–675 (2022). doi:10.1038/s41586-021-04389-z Medline

38. Y. Weisblum, F. Schmidt, F. Zhang, J. DaSilva, D. Poston, J. C. Lorenzi, F. Muecksch, M. Rutkowska, H.-H. Hoffmann, E. Michailidis, C. Gaebler, M. Agudelo, A. Cho, Z. Wang, A. Gazumyan, M. Cipolla, L. Luchsinger, C. D. Hillyer, M. Caskey, D. F. Robbiani, C. M. Rice, M. C. Nussenzweig, T. Hatziioannou, P. D. Bieniasz, Escape from neutralizing antibodies by SARS-CoV-2 spike protein variants. *eLife* **9**, e61312 (2020). doi:10.7554/eLife.61312 Medline

39. A. E. Lin, W. E. Diehl, Y. Cai, C. L. Finch, C. Akusobi, R. N. Kirchdoerfer, L. Bollinger, S. F. Schaffner, E. A. Brown, E. O. Saphire, K. G. Andersen, J. H. Kuhn, J. Luban, P. C. Sabeti, Reporter Assays for Ebola Virus Nucleoprotein Oligomerization, Virion-Like Particle Budding, and Minigenome Activity Reveal the Importance of Nucleoprotein Amino Acid Position 111. *Viruses* **12**, 105 (2020). doi:10.3390/v12010105 Medline

40. A. M. Syed, T. Y. Taha, M. M. Khalid, T. Tabata, I. P. Chen, B. Sreekumar, P.-Y. Chen, J. M. Hayashi, K. M. Soczek, M. Ott, J. A. Doudna, Rapid assessment of SARS-CoV-2 evolved variants using virus-like particles*bioRxiv*, 2021.08.05.455082 (2021).

41. M. M. Angelini, M. Akhlaghpour, B. W. Neuman, M. J. Buchmeier, Severe acute respiratory syndrome coronavirus nonstructural proteins 3, 4, and 6 induce double-membrane vesicles. *mBio* **4**, e00524-13 (2013). doi:10.1128/mBio.00524-13 Medline

42. R. L. Graham, A. C. Sims, S. M. Brockway, R. S. Baric, M. R. Denison, The nsp2 replicase proteins of murine hepatitis virus and severe acute respiratory syndrome coronavirus are dispensable for viral replication. *J. Virol.* **79**, 13399–13411 (2005). doi:10.1128/JVI.79.21.13399-13411.2005 Medline

43. I. Jungreis, R. Sealfon, M. Kellis, SARS-CoV-2 gene content and COVID-19 mutation impact by comparing 44 Sarbecovirus genomes. *Nat. Commun.* **12**, 2642 (2021). doi:10.1038/s41467-021-22905-7 Medline

44. M. R. Islam, M. N. Hoque, M. S. Rahman, A. S. M. R. U. Alam, M. Akther, J. A. Puspo, S. Akter, M. Sultana, K. A. Crandall, M. A. Hossain, Genome-wide analysis of SARS-CoV-2 virus strains circulating worldwide implicates heterogeneity. *Sci. Rep.* **10**, 14004 (2020). doi:10.1038/s41598-020-70812-6 Medline

45. C. T. Cornillez-Ty, L. Liao, J. R. Yates 3rd, P. Kuhn, M. J. Buchmeier, Severe acute respiratory syndrome coronavirus nonstructural protein 2 interacts with a host protein complex involved in mitochondrial biogenesis and intracellular signaling. *J. Virol.* **83**, 10314–10318 (2009). doi:10.1128/JVI.00842-09 Medline

46. M. Gupta, C. M. Azumaya, M. Moritz, S. Pourmal, A. Diallo, G. E. Merz, G. Jang, M. Bouhaddou, A. Fossati, A. F. Brilot, D. Diwanji, E. Hernandez, N. Herrera, H. T. Kratochvil, V. L. Lam, F. Li, Y. Li, H. C. Nguyen, C. Nowotny, T. W. Owens, J. K. Peters, A. N. Rizo, U. Schulze-Gahmen, A. M. Smith, I. D. Young, Z. Yu, D. Asarnow, C. Billesbølle, M. G. Campbell, J. Chen, K.-H. Chen, U. S. Chio, M. S. Dickinson, L. Doan, M. Jin, K. Kim, J. Li, Y.-L. Li, E. Linossi, Y. Liu, M. Lo, J. Lopez, K. E. Lopez, A. Mancino, F. R. Moss, M. D. Paul, K. I. Pawar, A. Pelin, T. H. Pospiech, C. Puchades, S. G. Remesh, M. Safari, K. Schaefer, M. Sun, M. C. Tabios, A. C. Thwin, E. W. Titus, R. Trenker, E. Tse, T. K. M. Tsui, F. Wang, K. Zhang, Y. Zhang, J. Zhao, F. Zhou, Y. Zhou, L. Zuliani-Alvarez, QCRG Structural Biology Consortium, D. A. Agard, Y. Cheng, J. S. Fraser, N. Jura, T. Kortemme, A. Manglik, D. R. Southworth, R. M. Stroud, D. L. Swaney, N. J. Krogan, A. Frost, O. S. Rosenberg, K. A. Verba, CryoEM and AI reveal a structure of SARS-CoV-2 Nsp2, a multifunctional protein involved in key host

processes. *bioRxiv* (2021), doi:10.1101/2021.05.10.443524.

47. Z. Jin, X. Du, Y. Xu, Y. Deng, M. Liu, Y. Zhao, B. Zhang, X. Li, L. Zhang, C. Peng, Y. Duan, J. Yu, L. Wang, K. Yang, F. Liu, R. Jiang, X. Yang, T. You, X. Liu, X. Yang, F. Bai, H. Liu, X. Liu, L. W. Guddat, W. Xu, G. Xiao, C. Qin, Z. Shi, H. Jiang, Z. Rao, H. Yang, Structure of M^{pro} from SARS-CoV-2 and discovery of its inhibitors. *Nature* **582**, 289–293 (2020). doi:10.1038/s41586-020-2223-y Medline

48. J. Osipiuk, S.-A. Azizi, S. Dvorkin, M. Endres, R. Jedrzejczak, K. A. Jones, S. Kang, R. S. Kathayat, Y. Kim, V. G. Lisnyak, S. L. Maki, V. Nicolaescu, C. A. Taylor, C. Tesar, Y.-A. Zhang, Z. Zhou, G. Randall, K. Michalska, S. A. Snyder, B. C. Dickinson, A. Joachimiak, Structure of papain-like protease from SARS-CoV-2 and its complexes with non-covalent inhibitors. *Nat. Commun.* **12**, 743 (2021). doi:10.1038/s41467-021-21060-3 Medline

49. H. S. Hillen, G. Kokic, L. Farnung, C. Dienemann, D. Tegunov, P. Cramer, Structure of replicating SARS-CoV-2 polymerase. *Nature* **584**, 154–156 (2020). doi:10.1038/s41586-020-2368-8 Medline

50. L. Yan, J. Ge, L. Zheng, Y. Zhang, Y. Gao, T. Wang, Y. Huang, Y. Yang, S. Gao, M. Li, Z. Liu, H. Wang, Y. Li, Y. Chen, L. W. Guddat, Q. Wang, Z. Rao, Z. Lou, Cryo-EM Structure of an Extended SARS-CoV-2 Replication and Transcription Complex Reveals an Intermediate State in Cap Synthesis. *Cell* **184**, 184–193.e10 (2021). doi:10.1016/j.cell.2020.11.016 Medline

51. J. Chen, B. Malone, E. Llewellyn, M. Grasso, P. M. M. Shelton, P. D. B. Olinares, K. Maruthi, E. T. Eng, H. Vatandaslar, B. T. Chait, T. M. Kapoor, S. A. Darst, E. A. Campbell, Structural Basis for Helicase-Polymerase Coupling in the SARS-CoV-2 Replication-Transcription Complex. *Cell* **182**, 1560–1573.e13 (2020). doi:10.1016/j.cell.2020.07.033 Medline

52. Y. Chen, H. Cai, J. Pan, N. Xiang, P. Tien, T. Ahola, D. Guo, Functional screen reveals SARS coronavirus nonstructural protein nsp14 as a novel cap N7 methyltransferase. *Proc. Natl. Acad. Sci. U.S.A.* **106**, 3484–3489 (2009). doi:10.1073/pnas.0808790106 Medline

53. Y. Huang, C. Yang, X.-F. Xu, W. Xu, S.-W. Liu, Structural and functional properties of SARS-CoV-2 spike protein: Potential antivirus drug development for COVID-19. *Acta Pharmacol. Sin.* **41**, 1141–1149 (2020). doi:10.1038/s41401-020-0485-4 Medline

54. J. Cubuk, J. J. Alston, J. J. Incicco, S. Singh, M. D. Stuchell-Brereton, M. D. Ward, M. I. Zimmerman, N. Vithani, D. Griffith, J. A. Wagoner, G. R. Bowman, K. B. Hall, A. Soranno, A. S. Holehouse, The SARS-CoV-2 nucleocapsid protein is dynamic, disordered, and phase separates with RNA. *Nat. Commun.* **12**, 1936 (2021). doi:10.1038/s41467-021-21953-3 Medline

55. Z. Chen, D. Pei, L. Jiang, Y. Song, J. Wang, H. Wang, D. Zhou, J. Zhai, Z. Du, B. Li, M. Qiu, Y. Han, Z. Guo, R. Yang, Antigenicity analysis of different regions of the severe acute respiratory syndrome coronavirus nucleocapsid protein. *Clin. Chem.* **50**, 988–995 (2004). doi:10.1373/clinchem.2004.031096 Medline

56. Center for Viral Systems Biology, A. A. Latif, J. L. Mullen, M. Alkuzweny, G. Tsueng, M. Cano, E. Haag, J. Zhou, M. Zeller, E. Hufbauer, N. Matteson, C. Wu, K. G. Andersen, A. I. Su, K. Gangavarapu, L. D. Hughes, Spike:D614G Mutation Report.
